# Identification of boron-deficiency-responsive microRNAs in *Citrus sinensis* roots by Illumina sequencing

**DOI:** 10.1186/1471-2229-14-123

**Published:** 2014-05-07

**Authors:** Yi-Bin Lu, Lin-Tong Yang, Yi-Ping Qi, Yan Li, Zhong Li, Yan-Bin Chen, Zeng-Rong Huang, Li-Song Chen

**Affiliations:** 1 College of Resources and Environmental Sciences, Fujian Agriculture and Forestry University, Fuzhou 350002, China; 2 Institute of Horticultural Plant Physiology, Biochemistry and Molecular Biology, Fujian Agriculture and Forestry University, Fuzhou 350002, China; 3 Institute of Materia Medica, Fujian Academy of Medical Sciences, Fuzhou 350001, China; 4 Fujian Key Laboratory for Plant Molecular and Cell Biology, Fujian Agriculture and Forestry University, Fuzhou 350002, China

**Keywords:** Boron-deficiency, Boron-tolerance, *Citrus sinensis*, Illumina sequencing, microRNA, Reactive oxygen species

## Abstract

**Background:**

Boron (B)-deficiency is a widespread problem in many crops, including *Citrus*. MicroRNAs (miRNAs) play important roles in nutrient deficiencies. However, little is known on B-deficiency-responsive miRNAs in plants. In this study, we first identified miRNAs and their expression pattern in B-deficient *Citrus sinensis* roots by Illumina sequencing in order to identify miRNAs that might be involved in the tolerance of plants to B-deficiency.

**Results:**

We isolated 52 (40 known and 12 novel) up-regulated and 82 (72 known and 10 novel) down-regulated miRNAs from B-deficient roots, demonstrating remarkable metabolic flexibility of roots, which might contribute to the tolerance of plants to B-deficiency. A model for the possible roles of miRNAs in the tolerance of roots to B-deficiency was proposed. miRNAs might regulate the adaptations of roots to B-deficiency through following several aspects: (*a*) inactivating reactive oxygen species (ROS) signaling and scavenging through up-regulating *miR474* and down-regulating *miR782* and *miR843*; (*b*) increasing lateral root number by lowering *miR5023* expression and maintaining a certain phenotype favorable for B-deficiency-tolerance by increasing *miR394* expression; (*c*) enhancing cell transport by decreasing the transcripts of *miR830*, *miR5266* and *miR3465*; (*d*) improving osmoprotection (miR474) and regulating other metabolic reactions (miR5023 and miR821). Other miRNAs such as *miR472* and *miR2118* in roots increased in response to B-deficiency, thus decreasing the expression of their target genes, which are involved in disease resistance, and hence, the disease resistance of roots.

**Conclusions:**

Our work demonstrates the possible roles of miRNAs and related mechanisms in the response of plant roots to B-deficiency.

## Background

Boron (B)-deficiency is a widespread problem in many agricultural crops, including *Citrus*. Over 132 crops are susceptible to B-deficiency, and low B availability in soils inhibits vegetative and reproductive growth in a large number of crops [1]. To cope with B-deficiency, plants have evolved a considerable degree of developmental plasticity, including adaptations *via* cascades of molecular networks. One of the most obvious features of the adaptations to B-deficiency is the changes in expression profiles of genes involved in a broad spectrum of biochemical, cellular and physiological processes, including B uptake and translocation, carbohydrate and energy metabolism, stress response, signaling and regulation, cell wall, protein process, nucleic acid metabolism, amino acid and fatty acid metabolism [2-5].

Small RNAs (sRNAs) have been identified as important post-transcriptional regulators of gene expression in plants. Based on the differences of biogenesis and function, endogenous sRNAs in plants can been divided into two classes, microRNAs (miRNAs) and small interfering RNAs (siRNAs). miRNAs, which are approx. 21-nucleotide (nt) in length and are generated from non-coding transcripts capable of forming imperfectly complementary hairpin structures by the RNase DICER-LIKE1 (DCL1) or DCL4, have been known to negatively regulate gene expression at the posttranscriptional level by specific binding and cleavage of their target mRNAs, or by repression of target mRNA translation [6]. Since the first identification of plant miRNAs in 2002 [7], increasing evidence shows that plant miRNAs play crucial roles in almost all biological and metabolic processes [8]. Therefore, miRNA-related research has become one of the hottest topics in plant biology.

In addition to their involvement in plant normal growth and development, miRNAs also regulate the adaptations of plants to biotic and abiotic stresses [8,9]. Evidence in *Arabidopsis thaliana*, tomato (*Solanum lycopersicum*), rapeseed (*Brassica napus*), rice (*Oryza sativa*), and common bean (*Phaselus vulgaris*) has demonstrated the important roles of miRNAs in phosphorus (P), nitrogen (N), sulfur (S) and cupper (Cu) deficiencies [10-13].

In *A. thaliana*, miR399 has been predicted to target three genes, which encode a phosphate transporter (PHT1;7), a ubiquitin-conjugating E2 enzyme, and a DEAD box helicase; however, only the E2 enzyme encoded by *UBC24* has been experimentally validated [14]. *miR399* is up-regulated in P-deficient roots and suppressed in P-sufficient roots and is negatively correlated with that of its target gene *UBC24*[15,16]. The inverse relationship of expression patterns between *UBC24* homologs and *miR399* under P-deficiency has been confirmed in common bean [17] and rice [18]. Transgenic *Arabidopsis* overexpressing *miR399* also had decreased level of *UBC24* transcripts [15]. In accordance with being inhibited by miR399s, *UBC24* down-regulates P uptake and root-to-shoot allocation. Phenotypes of both the *Arabidopsis* T-DNA knockout *ucb24* mutants and the *miR399*-overexpressing transgenic *Arabidopsis* plants resemble those of a previously reported *pho2* mutant, a P overaccumulator [19]. Therefore, miR399 plays important roles in maintaining P homeostasis by regulating *UBC24* transcript levels [20]. Following the first identification, more and more P-deficiency-responsive miRNAs are being identified in various plant species, including A*rabidopsis*[11,15,16,21], rapeseed [16], soybean (*Glycine max*) [21], white lupin (*Lupinus albus*) [22], *Medicago truncatula*[23], rice [15], switchgrass (*Panicum virgatum*) [24], common bean [13,17] and tomato [10]. So far, the majority of validated P-deficiency-responsive miRNA target genes are transcription factors, and other target genes mainly encode for abiotic/biotic stress-responsive proteins and enzymes related to protein modification/degradation [25]. The diverse functions of these target genes mean that a broad range of biological processes are coordinated in response to P-deficiency.

In *A. thaliana*, *miR395* is enhanced during sulfate-limitation, and its induction is controlled by a key transcription factor (SLIM1) in the S assimilation pathway [26]. Each plant miRNA regulates several genes, but usually the targets belong to the same gene family. However, miR395 targets members of the ATP sulfurylase (APS) gene family [14] and the sulfate transporter SULTR2;1 [26]. miR395 has been shown to mediate regulation of sulfate accumulation and allocation by targeting *APS* and *SULTR2;1*, respectively [12].

Recent work showed that in *Arabidopsis*, the expression of *miR397*, *miR398*, *miR408*, and *miR857* was induced by Cu-deficiency and negatively correlated with the accumulation of transcripts for Cu:zinc (Zn) superoxide dismutase (CSD1 and CSD2), COX5b-1 (a subunit of the mitochondrial cytochrome c oxidase), plantacyanin and laccases. It has been suggested that miRNA-mediated down-regulation is a general mechanism to regulate non-essential Cu proteins, thus allowing plants to save Cu for the most essential functions during Cu-starvation [27]. Also, miRNAs have been demonstrated to play important roles in response to N and iron (Fe) deficiencies [13,16,28]. Therefore, miRNAs may be involved in the adaptive responses of plant to B-deficiency. Recently, Ozhuner et al. [29] investigated B-toxicity-responsive miRNAs in barley roots and leaves and concluded that the signal transduction mechanism in leaves regulated by miR408 played an important role in barley B-tolerance. In addition, the expression level of *miR399* in barley roots and leaves was differentially regulated by B-toxicity. However, little information about B-deficiency-responsive plant miRNAs is available.

Identification of miRNAs is a key step for understanding their regulatory functions in plants. Plant miRNAs were discovered by both experimental and computational approaches. However, both the computational approach by searching for homologous sequences using EST or genomic sequences and the small-scale traditional sequencing approach are mostly limited to the identification of conserved miRNAs [30]. Recently developed high-throughput sequencing techniques (e.g. 454 technology and Illumina platform) have become powerful tools to uncover the large list of sRNA species in plants. These deep sequencing strategies may identify both known and novel miRNAs at unprecedented sensitivities and provide quantitative profiling of miRNA expression [11].

*Citrus* belong to evergreen subtropical fruit trees and are commercially grown in many countries. In 1936, Morris first described B-deficiency in field grown *Citrus* in South Africa [31]. In China, B-deficiency is frequently observed in *Citrus* orchards and is responsible for loss of productivity and poor fruit quality [32]. Although the effects of B-deficiency on *Citrus* growth, mineral nutrients, B uptake and distribution, CO_2_ assimilation, photosystem II photochemistry, photosynthetic enzymes, respiration, carbohydrate metabolism, antioxidant system and proteomics have been examined in some details [32-37], no data are available on B-deficiency-responsive miRNAs in *Citrus*. In this study, we reported the high-throughput sequencing (Illumina) analysis of sRNAs from roots of *Citrus sinensis* seedlings grown in B-sufficient (control) and -deficient nutrient solution with the objectives of identifying miRNAs that might be involved in the tolerance of plants to B-deficiency.

## Results

### Plant growth and B concentration in roots and leaves

As shown in Table 1, 0 μM B treatment decreased shoot and whole plant dry weight (DW), and B concentration in roots and leaves, increased the ratio of root DW to shoot DW, but did not affect root DW. B concentration in 0 μM B-treated leaves was much lower than the sufficiency range of 30 to 100 μg g^−1^ DW [38]. Based on these results, plants treated with 0 μM B are considered B-deficient, and those treated with 10 μM B are considered B-sufficient.

**Table 1:** Effects of B-deficiency on plant growth and B concentration in roots and leaves

***B treatments***	***Root DW (g plant***^***−1***^ ***)***	***Shoot DW (g plant***^***−1***^ ***)***	***Whole plant DW (g plant***^***−1***^ ***)***	***Root DW/ Shoot DW***	***B concentration (μg g***^***−1***^ ***DW)***	
					**Roots**	**Leaves**
Control	10.03 ± 1.05 a	29.01 ± 3.02 a	39.04 ± 3.89 a	0.35 ± 0.03 b	12.20 ± 1.15 a	32.54 ± 2.53 a
B-deficiency	9.39 ± 1.10 a	18.47 ± 1.33 b	27.86 ± 2.02 b	0.51 ± 0.06 a	8.26 ± 0.79 b	12.17 ± 0.47 b

Data are means ± SD from 4 to 7 replicates. Within a column, values followed by different letters are significantly different at *P* < 0.05 detected by the unpaired *t*-test.

### High-throughput sequencing and annotation of miRNAs in roots

Two libraries were constructed from *C. sinensis* roots subjected to 0 or 10 μM B for 15 weeks, respectively. These libraries were sequenced by Illumina sequencing, leading to the generation of 22,998,100 and 22,576,217 raw reads from libraries of control and B-deficiency, respectively (Table 2). After removal of adaptors, low quality tags and contaminants, the length distribution of clean reads was summarized in Additional file 1. Reads with length of 24 nt were at the most abundant, followed by the reads with length of 22 nt and 21 nt. B-deficiency resulted in fewer 22 nt and 24 nt reads and more 20 nt and 23 nt reads. Generally speaking, the length distribution of sRNAs was similar to previous reports in higher plants such as sweet orange (*Citrus sinensis*) [30], *Medicago truncatula*[39], *Arabidopsis*[40] and trifoliate orange (*Citrus trifoliata*) [41]. This suggests that the Illumina sequencing data of sRNA libraries is reliable.

**Table 2:** **Summary of sRNAs from control and B-deficient roots of** ***Citrus sinensis***

	**Controls**		**B-deficiency**	
	**Unique sRNAs**	**Total sRNAs**	**Unique sRNAs**	**Total sRNAs**
Raw reads		22998100		22576217
Clear reads	4505507 (100%)	21668361 (100%)	4704810 (100%)	21530509 (100%)
Mapped to genomic	2343700 (52.02%)	16586059 (76.55%)	2669645 (56.74%)	16889709 (78.45%)
Exon antisense	91162 (2.02%)	310775 (1.43%)	105747 (2.25%)	361376 (1.68%)
Exon sense	236699 (5.25%)	586775 (2.71%)	233380 (4.96%)	605450 (2.81%)
Intron antisense	50614 (1.12%)	185709 (0.86%)	60167 (1.28%)	201017 (0.93%)
Intron sense	92377 (2.05%)	577538 (2.67%)	107307 (2.28%)	691496 (3.21%)
miRNA	51603 (1.15%)	5003869 (23.09%)	41144 (0.87%)	5618205 (26.09%)
rRNA	168606 (3.74%)	3864145 (17.83%)	101906 (2.17%)	2525627 (11.73%)
snRNA	4057 (0.09%)	17352 (0.08%)	3651 (0.08%)	16305 (0.08%)
snoRNA	1630 (0.04%)	4804 (0.02%)	1952 (0.04%)	6788 (0.03%)
tRNA	32162 (0.71%)	1005187 (4.64%)	19256 (0.41%)	749792 (3.48%)
Unannotated sRNAs	3776597 (83.82%)	10112207 (46.67%)	4030300 (85.66%)	10754453 (49.95%)

As shown in Table 2, 16,586,059 clean reads (2,343,700 unique reads) from control and 16,889,709 clean reads (2,669,645 unique reads) from B-deficient roots were mapped to *C. clementina* genome using SOAP. Exon, intron, miRNA, rRNA, snRNA, snoRNA and tRNA reads were annotated, respectively. Reads used for prediction of novel miRNAs for control and B-deficient roots were 3,776,597 and 4,030,300, respectively.

### Identification of known miRNAs in roots

To identify the known miRNAs in the two libraries constructed from control and B-deficient roots, clear reads were aligned with known plant miRNAs from miRBase 18 (http://www.mirbase.org/). Only the perfectly matched sequences were considered. A total of 538 known miRNAs was identified in the two libraries (Additional file 2). To compare the abundance of miRNAs in different libraries, the count of reads was normalized to TPM. In control library, the most abundant miRNA identified was miR3954 (83,883.0865 TPM), followed by miR156 (32,511.4115 TPM) and miR166 (22,220.8316 TPM). However, miR156 abundance (22,980.3671 TPM) in B-deficient library ranked third after miR3954 (122,762.6342 TPM) and miR166 (42,066.0747 TPM) (Additional file 2). The known miRNAs with normalized read-count less that ten in the two libraries were not used for further analysis, because the use of low expressed miRNAs is apt to cause false results [39]. After removing these miRNAs, a total of 238 miRNAs were further analyzed (Additional file 3).

### Identification of novel miRNAs in roots

After removal of the rRNAs, snRNAs, snoRNAs, tRNAs and known miRNAs, the remained sequences that were not annotated were used to predict novel miRNAs using the Mireap (http://sourceforge.net/projects/mireap/). Based on the criteria for annotation of plant miRNAs [6,42], we identified a total of 108 novel miRNAs from control and B-deficient roots (Additional file 4). Similar to the known miRNAs, novel miRNAs with very low expression were excluded from the expression analysis [39], thus leading to 60 miRNAs that were used for further analysis (Additional file 5).

### Differentially expressed miRNAs between control and B-deficient roots

A miRNA was considered differentially expressed when the miRNA had both a fold-change of more that 1.5 and a *P*-value of less than 0.01. Based on the two criteria, 40 known and 12 novel miRNAs were up-regulated, and 72 known and 10 novel miRNAs were down-regulated in response to B-deficiency (Additional files 3 and 5).

### Validation of high-throughput sequencing results by real time quantitative reverse transcription PCR (qRT-PCR)

**Figure 1:**
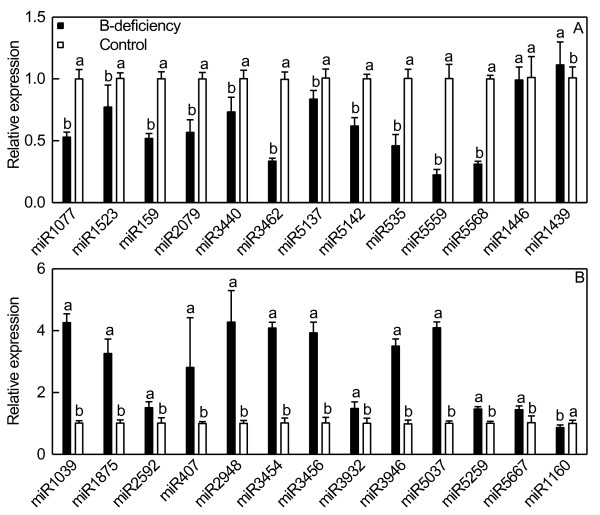
**Relative abundances of selected known miRNAs in B-deficient and control roots revealed by qRT-PCR.** Bars represent mean ± SD (*n* = 3). Significant differences were tested between control and B-deficient roots for the same miRNA. Different letters above the bars indicate a significant difference at *P* < 0.05. All the values were expressed relative to the control roots.

As only one mixed sample of B-deficient and control RNA was sequenced, it was necessary to measure the expression of a selection of miRNAs to validate that the changes observed were biologically consistent. qRT-PCR analysis showed that 23 of the 26 miRNAs tested were comparable in magnitude to the expression profiles obtained by the high-throughput sequencing (Figure 1). This technique was thus validated in 88.5% of cases.

### Prediction of targets for differentially expressed miRNAs

**Figure 2:**
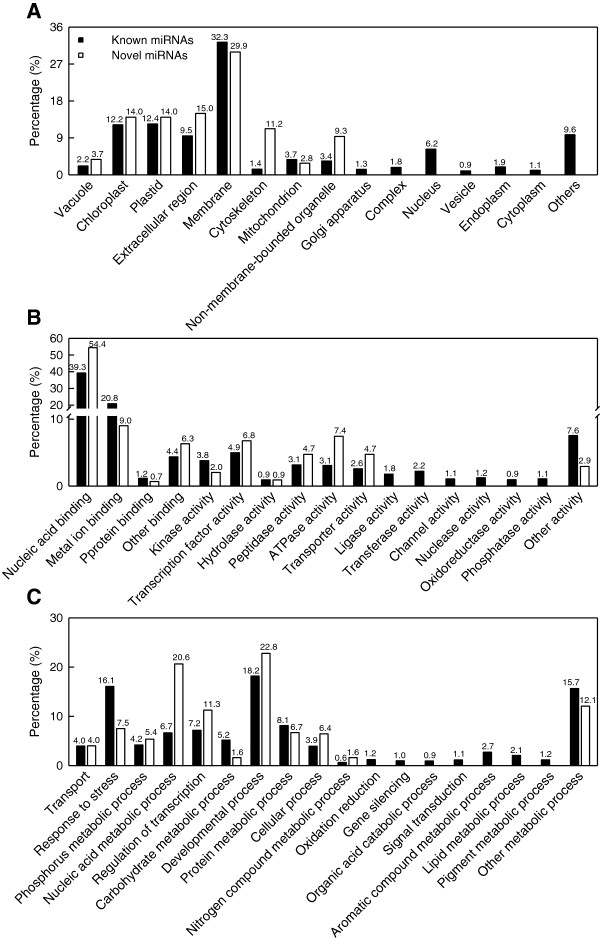
**GO of the predicted target genes for 122 (22) differentially expressed known (novel) miRNAs.** Categorization of miRNAs target genes was performed according to cellular component **(A)**, molecular function **(B)** and biological process **(C)**.

A total of 1228 (103) genes were predicted based on the 112 (22) differentially expressed known (novel) miRNAs in *C. sinensis* roots (Additional files 6 and 7). GO categories were assigned to all the predicted targets according to the cellular component, molecular function and biological process. Categories based on the cellular component showed that the potential targets for the 112 and 22 differentially expressed known and novel miRNAs were associated with 15 and 8 components, respectively, with the highest percentage of membrane (Figure 2A). Based on the molecular function, the targets for the known and novel miRNAs were classified into 17 and 11 categories, respectively, the highest percentage of two categories were nucleic acid binding and metal ion binding (Figure 2B). As shown in Figure 2C, the known and novel miRNAs targets were involved in 18 and 11 biological processes, respectively, the most two GO terms are developmental process and response to stress for known miRNAs and developmental process and nucleic acid metabolism process for novel miRNAs, respectively.

### qRT-PCR relative expression analysis of target genes

In plants, genes targeted by miRNAs are believed to be regulated mainly *via* endonucleolytic cleavage of mRNAs due to their near-perfect complementarity to their target genes, although evidence indicates the existence of widespread translational inhibition [43]. Twenty-nine genes targeted by 10 down-regulated and two up-regulated miRNAs were assayed by qRT-PCR (Table 3). Seventeen of the 29 target genes had the expected changes in mRNA levels, suggesting that miRNAs play a role in regulating gene expression under B-deficiency by cleaving mRNAs. However, the expression changes of 11 target genes displayed a positive correlation with their corresponding miRNAs. The remaining one target gene was not detected in control and B-deficient roots. Overall, there was no obvious pattern in the expression profiles of target genes in response to B-deficiency. For example, the relative expression levels of nine genes targeted by down-regulated *miR157* were validated by qRT-PCR. Four genes displayed decreased expression, while five had increased expression. The results were consistent with those reported in *Arabidopsis*[40].

**Table 3:** qRT-PCR relative expression of experimentally determined or predicted target genes of selected miRNAs

**miRNA**	**Fold change of miRNA**	**Accession**	**Homology**	**Target genes**	**Relative change of target genes**
miR157	−1.91565867**	**clementine0.9_008930m|PACid:19258970**	**At1g27370**	**SPL10**	3.4986**
		**clementine0.9_022829m|PACid:19252333**	**At3g57920**	**SPL15**	2.6067**
		**clementine0.9_008954m|PACid:19258969**	**At5g43270**	**SPL2**	1.4237**
		**clementine0.9_024862m|PACid:19271517**	**AT2G33810.1**	**SPL3**	1.3488**
		clementine0.9_013242m|PACid:19264658	At2g42200	SPL9	0.3869**
		clementine0.9_008011m|PACid:19259369	At1g69170	SPL6	0.4757**
		clementine0.9_016441m|PACid:19260022	At5g50570	SPL13, SPL13A	0.3484**
		clementine0.9_023574m|PACid:19252334	At1g53160	SPL4	0.5714**
		clementine0.9_002919m|PACid:19270659	AT5G45650.1	Subtilase family protein	0.6551**
miR158	−10.05808647**	clementine0.9_033221m|PACid:19268186	AT2G03210	Fucosyltransferase 2	0.5617**
		clementine0.9_001239m|PACid:19279142	AT3G07400	Lipase class 3 family protein	0.2308**
miR165	−1.72183503**	**clementine0.9_002294m|PACid:19282243**	**At5g60690**	**IFL1/REV**	1.8727**
		**clementine0.9_002262m|PACid:19273192**	**At4g32880**	**ATHB-8**	2.9758**
		**clementine0.9_002420m|PACid:19255038**	**At1g52150**	**ATHB-15**	3.1719**
miR2118	1.80829542	**clementine0.9_000380m|PACid:19257198**	**AT3G14460.1**	**LRR and NB-ARC domains-containing disease resistance protein**	0.3102**
		clementine0.9_001085m|PACid:19277494	AT5G17680.1	TIR-NBS-LRR domain protein	ND
miR472	1.57070884**	**clementine0.9_030591m|PACid:19255601**	**AT4G27190.1**	**Disease resistance protein (TIR-NBS-LRR class) family**	0.2364**
		**clementine0.9_002232m|PACid:19255072**	**AT5G63020.1**	**LRR and NB-ARC domains-containing disease resistance protein**	0.4072**
		**clementine0.9_001280m|PACid:19266747**	**AT1G12210.1**	**Disease resistance protein (CC-NBS-LRR class) family**	0.5369**
miR782	−10.76475548**	clementine0.9_012930m|PACid:19252541	AT2G19810.1	CCCC-type zinc finger family protein	0.3491**
miR830	−5.62148264**	clementine0.9_010529m|PACid:19254979	At1g52380	RanBP1 domain	0.7394**
		**clementine0.9_001127m|PACid:19282371**	**At3g45850**	**Kinesin motor-related**	2.1131**
miR843	−10.39121131**		**TC375153**	**Leucine rich repeat protein**	1.9240**
		clementine0.9_003787m|PACid:19284123	At5g13550	Sulphate transporter	0.7840**
miR5023	−10.93425838**	**clementine0.9_013684m|PACid:19283327**	**AT3G21640.1**	**FKBP-type peptidyl-prolyl cis-trans isomerase family protein**	1.0678*
		**clementine0.9_002391m|PACid:19286425**	**AT5G45160.1**	**Root hair defective 3 GTP-binding protein (RHD3)**	1.8507**
miR5266	−1.5614939**	**clementine0.9_008179m|PACid:19257317**	**AT4G13510.1**	**Ammonium transporter 1;1**	1.3396*
miR5562	−1.67229975**	clementine0.9_008349m|PACid:19251662	AT2G38290.1	Ammonium transporter 2	0.4023**
miR3465	−3.3743459**	**clementine0.9_001178m|PACid:19280124**	**AT3G57330.1**	**Autoinhibited Ca**^**2+**^**-ATPase 11**	1.5827**

Both fold change of miRNAs and relative change of target genes are the ratio of B-deficient roots to the controls. The value for relative change of target gene is an average of at least three biological replicates with three technical replicates; Target genes that had the expected changes in mRNA levels were marked in bold. ND: Not detected; *and **indicate a significant difference at *P* < 0.05 and *P* < 0.01, respectively.

### Root metabolites and enzymes

**Figure 3:**
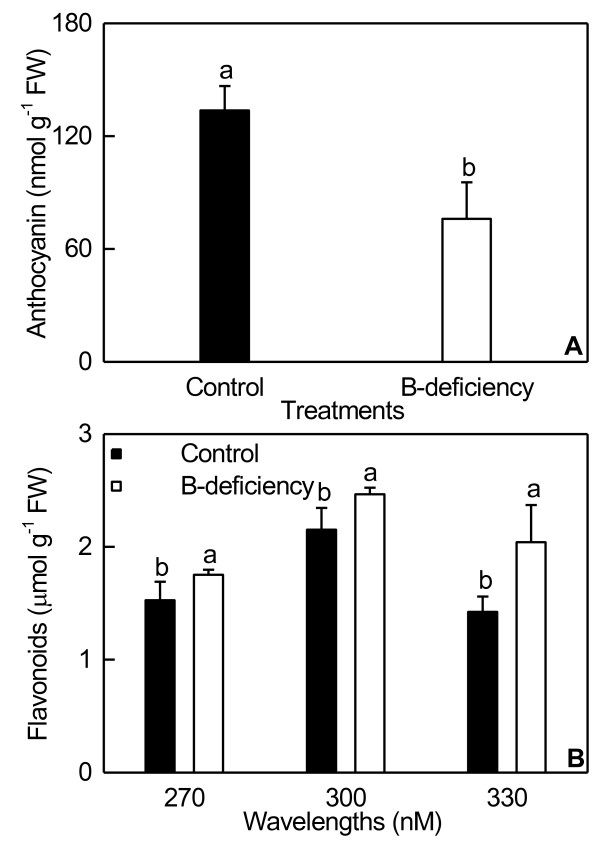
**Effects of B-deficiency on root concentrations of anthocyanin (A) and flavonoids (B).** Bars represent mean ± SD (*n* = 7). Significant differences was tested between B-deficient and control roots. Different letters above the bars indicate a significant difference at *P* < 0.05.

**Figure 4:**
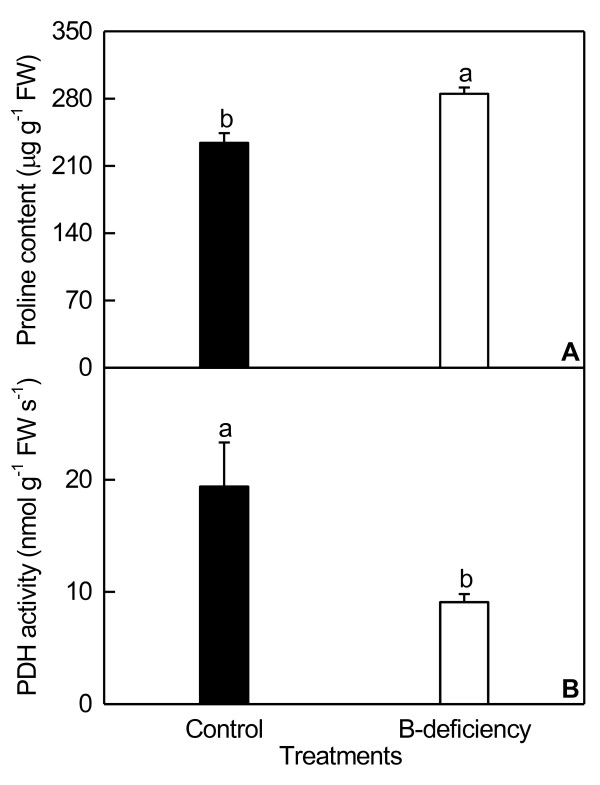
**Effects of B-deficiency on proline concentration (A) and proline dehydrogenase activity (B) in roots.** Bars represent mean ± SD (*n* = 4). Different letters above the bars indicate a significant difference at *P* < 0.05.

**Figure 5:**
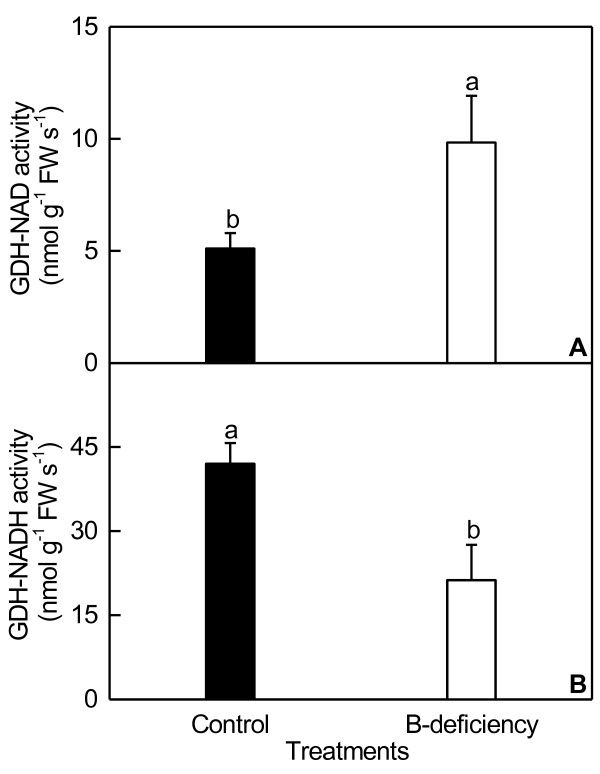
**Effects of B-deficiency on GDH-NAD activity (A) and GDH-NADH activity (B) in roots.** Bars represent mean ± SD (*n* = 4 or 6). Different letters above the bars indicate a significant difference at *P* < 0.05.

B-deficient roots displayed decreased concentration of anthocyanin and increased levels of flavonoids (Figure 3). B-deficiency increased root proline concentration, and decreased its proline dehydrogenase (PDH) activity (Figure 4). As shown in Figure 5, B-deficient roots had increased glutamate dehydrogenase (GDH)-NAD (deaminative) activity, but decreased GDH-NADH (aminative) activity.

## Discussion

As important post-transcriptional regulators, the expressions of many plant miRNAs are regulated by various biotic and abiotic stresses, including nutrient (S, Cu, P, Fe and N) deficiencies, which may contribute to the development of adaptive responses to deal with unfavorable growth conditions [8,9,11,13,16,26,44]. Although the genes responsible for tolerance of plants to B-deficiency has been examined in some detail [4,5], little information is available on the roles of miRNAs under B-deficiency. In this study, we identified 538 known miRNAs (Additional file 2) and 108 novel miRNAs (Additional file 4) from control and B-deficient roots. A 1.5-fold cut-off was set to determine up-regulated and down-regulated miRNAs in addition to a *P*-value of less than 0.01. Based on the two criteria, 52 (40 known and 10 novel) up-regulated miRNAs and 82 (70 known and 10 novel) down-regulated miRNAs were identified in B-deficient roots (Additional files 3 and 5), demonstrating that the expression profiles of miRNAs in B-deficient roots were greatly affected.

Our finding that root *miR474* was up-regulated under B-deficiency (Additional file 3) agrees with the previous results obtained on drought-stressed rice and maize (*Zea mays*) leaves [45] and salt-stressed maize roots [46]. Wei et al. [45] showed that under drought stress, the transcript of *miR474* and the concentration of proline were increased in maize, whereas its target gene *PDH* was down-regulated. They concluded that drought-induced increase in *miR474* expression might down-regulate *PDH*, thus increasing the accumulation of proline, an osmoprotectant. In addition to improving osmoprotection responses to drought stress, proline acts as a free radical scavenger to protect plants from oxidative damage. Hajiboland and Bastani [47] observed that proline concentration in tea roots and leaves increased under B-deficiency and drought stress, especially in roots, suggesting that the B-deficiency-induced accumulation of proline may be a strategy for tea plants to counteract the oxidative stress. Therefore, proline level in B-deficient roots might be enhanced due to decreased PDH activity resulting from enhanced expression level of *miR474*, thus improving the adaptation of plants to B-deficiency. As expected, B-deficient roots displayed decreased PDH activity and increased proline concentration (Figure 4).

We found that *miR157* in roots was down-regulated by B-deficiency (Additional file 3), as previously obtained on N- and P- deficient common bean roots. However, common bean root *miR157* was up-regulated by Fe-deficiency and manganese (Mn)-toxicity [13]. In plants, miR156 and miR157 have been grouped in one miRNA family because of their high degree of sequence similarity and their conserved targets, the squamosa promoter binding protein-like (SPL) proteins [48]. Gou et al. [49] observed a positive relationship of anthocyanin concentration and miR156 activity and an inverse relationship of flavonol (a subclasse of flavonoid) concentration and miR156 activity. As expected, B-deficient roots had lower anthocyanin concentration and higher flavonoid concentration (Figure 4) due to decreased expression of miR157. Gou et al. [49] suggested that at one of the miR156 targets, *SPL9*, negatively regulates anthocyanin accumulation by directly inhibiting expression of anthocyanin biosynthetic genes through disruption of the MYB-bHLH-WD40. However, *SPL9* in *C. sinensis* roots was down-regulated by B-deficiency (Table 3).

Our observation that root *miR158* was decreased under B-deficiency (Additional file 3) agrees with the previous report that *miR158* was down-regulated in P-deficient tomato roots [10] and N-deficient *Arabidopsis* seedlings [28]. However, Buhtz et al. [50] observed that *miR158* was up-regulated in the phloem of *B. napus* under Fe-deficiency. In *Arabidopsis* seedlings, *miR158* were up-regulated in response to salt stress [51]. Expression of *miR158a* in *Arabidopsis* roots increased under hypoxia [40]. miR158 is predicted to target three genes encoding a pentatricopeptide repeat containing protein of unknown function, fucosyltransferases (xyloglucan fucosyltransferases) and a lipase [52]. However, root expression levels of *fucosyltransferase 2* and *lipase class 3 family protein* decreased in response to B-deficiency (Table 3).

Root transcript of *miR5023* decreased in response to B-deficiency (Additional file 3). This means that its target genes: *FKBP-type peptidyl-prolyl cis-trans isomerase family protein* and *root hair defective 3 GTP-binding protein (RHD3)* (Additional file 6), might be up-regulated under B-deficiency. This is validated by our qRT-PCR data that the expression of the two genes in roots increased in response to B-deficiency (Table 3). FK506-binding proteins (FKBP), cyclosporins (CyPs) and parvulin (Pvn) are the three major classes of peptidyl prolyl cis-trans isomerases (PPIases), which are considered to assist chaperones by accelerating the slow rate-limiting isomerization steps. Among these, the best-studied class of PPIases is that of FKBPs by far. Increasing evidence shows that in addition to their role in protein folding, plant FKBPs are involved in abiotic stress response [53]. Nigam et al. [54] showed that yeast cells overexpressing *FKBP20* displayed enhanced tolerance to high temperatures. The up-regulation of *RHD3* in B-deficient roots agrees with the previous results obtained by Yang et al. [37] in B-deficient *C. sinensis* roots and by Redondo-Nieto et al. [4] in B-deficient *M. truncatula* root nodules. In *Arabidopsis*, RHD3 has been suggested to be required for cell wall biosynthesis and actin organization [55]. Xu et al. [56] observed that transgenic poplar plants overexpressing *PeRHD3* had less adventitious roots, more lateral roots, and longer and more root hairs. Thus, the number of lateral roots in B-deficient *C. sinensis* seedlings might increase due to increased expression level of *RHD3*. This agrees with the previous reports data that B-deficiency increased lateral root formation of plants [57]. These results imply that RHD3 may be involved in the tolerance of plants to B-deficiency.

Zhou et al. [58] reported that overexpression of *miR165* resulted in a drastic reduction in the transcript levels of its target genes [all five class III homeodomain leucine-zipper (HD-ZIP III) genes] in *Arabidopsis* seedlings. Hawker and Bowman [59] showed that *HD-ZIP III* genes played a role in promoting *Arabidopsis* lateral root formation. Therefore, B-deficient roots might have increased expression level of *HD-ZIP III* due to decreased abundance of *miR165* (Additional file 3), thus enhancing lateral root formation. Indeed, qRT-PCR analysis showed that the expression levels of three HD-ZIP III transcription factors (*ILF1/REV, ATHB-8 and ATHB-15*) increased in B-deficient roots (Table 3). This is also supported by the previous reports that B-deficiency increased the lateral root formation [57].

Recently, Xu et al. [30] observed an inverse relationship between the abundance of *miR1857* in the red-flesh mutant of sweet orange and the expression level of its target gene encoding lycopene β-cyclase, a key enzyme of the carotenoid biosynthesis pathway. In our study, root *miR1857* was was up-regulated under-B deficiency (Additional file 3), implying that carotenoid biosynthesis might be impaired in B-deficient roots due to decreased expression of lycopene β-cyclase gene.

Our finding that root *miR2118* was induced by B-deficiency (Additional file 3) agrees with the previous reported that the transcript of *miR2118* was enhanced in salt-stressed roots of *Vigna unguiculata*[60], NaCl, drought and ABA treated *P. vulgaris* seedlings [61], Fe-deficient common bean leaves [13], and drought-stressed *M. truncatula* shoots [62], and with our qRT-PCR data that the expression of one target gene encoding LRR and NB-ARC domains-containing disease resistance protein decreased in B-deficient roots (Table 3). However, P-deficiency and Mn-toxicity down-regulated the transcript of *miR2118* in common bean leaves [13]. Wong et al. [63] reported that the abundance of disease resistance protein (TIR-NBS-LRR class, a target gene of miR2118) mRNA in *Thellungiella salsuginea* leaves was down-regulated in response to drought and short-term salinity stress. However, the abundance of TIR-NBS-LRR class disease resistance protein in *Thellungiella halophila* leaves increased under long-term salinity stress [64]. Therefore, miR2118 might be involved in abiotic stresses as well as biotic stresses.

Evidence shows that the target genes of miR472 are involved in disease resistance [65]. We found that *miR472* was enhanced in B-deficient roots (Additional file 3), meaning that disease resistance protein gene might be down-regulated, thus decreasing the disease resistance of roots. As expected, the expression of three genes encoding disease resistance protein (TIR-NBS-LRR class) family, LRR and NB-ARC domains-containing disease resistance protein, and disease resistance protein (CC-NBS-LRR class) family decreased in B-deficient roots (Table 3). This agrees with the fact that B increases the disease resistance in plants [66].

We found that root *miR394* was up-regulated by B-deficiency (Additional file 3), as previously reported on P-starved tomato roots [10], NaCl-treated *Arabidopsis* seedlings [51], Fe-deficient *Malus xiaojinensis* roots [67], and N-deficient maize shoots (*miR394s*) [44]. Ni et al. [68] showed that overexpression of *miR394a* in *Arabidopsis* reduced the transcript of an *F-box gene* (At1g27340, also known as LEAF CURLING RESPONSIVENESS, LCR) containing a miR394 complementary target site. In *Arabidopsis*, a null mutation in *DOR* gene, which encodes a putative F-box protein, led to a substantial increase in drought tolerance as well as a hypersensitive ABA response of stomatal closing; conversely, the transgenic plants overexpressing *DOR* gene showed decreased drought tolerance [69]. Song et al. [70] reported that both *miR394* and *LCR* transcripts were regulated by salt and drought stresses and ABA treatment, concluding that the silencing of *LCR* mRNA by miR394 is essential to maintain a certain phenotype favorable for the adaptive response to abiotic stresses. Therefore, B-deficiency might down-regulate the accumulation of *LCR* mRNA in *C. sinensis* roots due to increased transcript of *miR394* (Additional file 3), thus improving the tolerance of plants to B-deficiency. However, Li et al. [71] observed that *miR394a* was up-regulated in response to drought stress but down-regulated in response to salinity stress in soybean roots. *miR394a,b,c* were up-regulated in roots, stems and leaves of *B. napus* by sulfate-deficiency and Cd stress except for the down-regulation of *miR394* in sulfate-deficient leaves [72]. In *Arabidopsis*, root *miR394b* and shoot *miR394a* and *miR394b* were initially up-regulated and then down-regulated under Fe-deficiency [73].

miR782 is predicted to target genes encoding maize protein disulfide isomerase (PDIL5-1) [74] and MYB transcription factor (MYBML2) [75]. Our result showed that root expression of *miR782* decreased in response to B-deficiency (Additional file 3), implying that the expression of *PDI* and *MYBML2* might be up-regulated in B-deficient roots. Protein disulfide isomerases (PDIs) are molecular chaperones that contain thioredoxin (TRX) domains and aid in the formation of proper disulfide bonds during protein folding. Chen et al. [76] showed that transgenic rice seedlings overexpressing a protein disulfide isomerase-like protein (PDIL) gene displayed enhanced tolerance to mercury (Hg), accompanied by lower levels of superoxide anion radicals, H_2_O_2_ and malondialdehyde (MDA), higher activities of superoxide dismutase (SOD) and peroxidase (POD), and increased concentrations of non-protein thiols and reduced glutathione (GSH). Plant MYB proteins are characterized by a highly conserved MYB DNA-binding domain. Plant MYB transcription factors are involved in regulatory networks controlling development, metabolism and responses to biotic and abiotic stresses. Rubio et al. [77] showed that a conserved MYB transcription factor was involved in phosphate starvation signaling in both vascular plants and in unicellular algae. Thus, the down-regulation of *miR782* in B-deficient roots might provide an adaptive strategy of plants to B-deficiency.

B-deficiency decreased the transcript of root *miR830* (Additional file 3). This agrees with the previous report that *miR830a* in *Arabidopsis* seedlings was down-regulated at low temperature (16°C) [78]. However, *miR830* was up-regulated in P-deficient roots and down-regulated in P-deficient stems and leaves of white lupin [22]. miR830 is predicted to target two genes encoding RanBP1 domain-containing protein and kinesin motor-related [79]. qRT-PCR analysis showed that B-deficient roots had increased expression of kinesin motor-related, but decreased expression of *RanBP1 domain-containing protein* (Table 3). Kinesins, a superfamily of microtubule motor proteins ubiquitous in all eukaryotic organisms, function in the unidirectional transport of vesicles and organelles, cytokinesis, signal transduction, and morphogenesis [80]. Therefore, the down-regulation of *miR830* in B-deficient roots might be advantageous to normal growth and development of plants under B-deficiency.

We found that the transcript of *miR843* decreased in B-deficient roots (Additional file 3), implying that its target genes: *leucine rich repeat protein (LRP)*, *plant homedomain (PHD)-finger*, *oligomeric golgi complex 7-like*[81] and *sulfate transporter*[82], might be up-regulated under B-deficiency. qRT-PCR analysis showed that B-deficient roots had enhanced expression of *LRP*, but decreased expression of *sulfate transporter* (Table 3). Park et al. [83] observed that heterologous expression of rice *LRP* (*OsLRP*) resulted in the activation of defense response and enhanced resistance to bacterial soft rot in Chinese cabbage. Thus, the higher expression of *LRP* might partially compensate for the decreased disease resistance in B-deficient plants [66]. Wei et al. [84] reported that transgenic *Arabidopsis* plants overexpressing the *GmPHD2* from soybean displayed enhanced salt tolerance through control of ROS signaling and scavenging. Liu et al. [85] observed that overexpression of *OsPHD1* enhanced the tolerance of transgenic rice plants to drought, high salt and cold stresses. Therefore, the down-regulation of *miR843* in B-deficient roots might be an adaptive response. Our observation that B-deficiency decreased the transcript of root *sulfate transporter* (Table 3) disagrees with the previous reports that B-deficiency increased the abundances of phosphate transporter 3;1 [37] in *C. sinensis* roots and of K^+^ channel in *B. napus* roots [86].

We observed that B-deficiency increased the transcript of *miR821* in roots (Additional file 3), which agrees with the previous data that *miR821* was expressed in roots of salt-stressed plants, and not expressed in healthy, non-stressed plants [87]. Down-regulation of its target gene, *putative enoyl-CoA hydratase/isomerase* by miR821 is indicative of the impact of the β-oxidation pathway of unsaturated fatty acids, which might lead to decrease in carbon flux in the form of acetyl-CoA. The acetyl-CoA can eventually enter the TCA cycle [88]. This agrees with our report that B-deficient *C. sinensis* roots displayed decreased root respiration [37]. In this study, the transcript of *GDH1*, a target gene of miR821 [89], might be down-regulated in B-deficient roots, thus decreasing the activity of root GDH. As expected, GDH-NADH (aminative) activity was down-regulated in B-deficient *C. sinensis* roots (Figure 5B). However, GDH-NAD (deaminative) activity in roots increased in response to B-deficiency (Figure 5A). Robinson et al. [90] reported that the primary role of GDH was the oxidation of glutamate under conditions where carbon is limited. Thus, B-deficiency-induced increase in GDH-NAD activity agrees with the previous study showing that B-deficiency decreased or did not affect root concentrations of non-structural carbohydrates [37]. Beato et al. [91] showed that *GDH* genes, *Ntgdh-NAD;A1* and *Ntgdh-NAD;B2*, were up-regulated in N-deficient tobacco roots, accompanied by decreased concentrations of glucose and fructose.

B-deficiency decreased the expression level of root *miR5266* (Additional file 3). As expected, the transcript of *ammonium transporter 1;1* targeted by miR5266 (Additional file 6) was enhanced in B-deficient roots (Table 3), hence facilitating the uptake of ammonium from external environments as well as the translocation of ammonium from roots to shoots [92]. This agrees with the previous reports that B-deficient tobacco roots had higher concentration of ammonium [2]. The higher uptake of ammonium might compensate for the reduced nitrate uptake by repressing root plasmalemma H^+^-ATPase (*PMA2*) gene expression [2]. However, the expression of *ammonium transporter 2* targeted by miR5562 (Additional file 6) decreased in B-deficient roots (Table 3).

The expression of autoinhibited Ca^2+^-ATPase 11 gene (a target gene of miR3465, Additional file 6), might be up-regulated in B-deficient roots due to decreased transcript of *miR3465* (Additional file 3). This agrees with our finding that B-deficiency increased the abundances of autoinhibited Ca^2+^-ATPase 11 in *C. sinensis* roots [37].

## Conclusions

**Figure 6:**
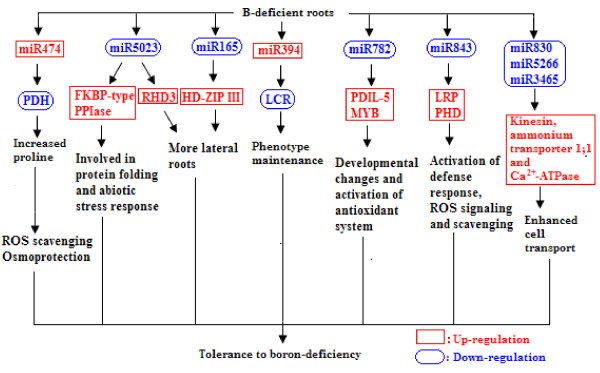
**A proposed model for the possible roles of miRNAs in the tolerance of *Citrus sinensis *** **roots to B-deficiency.**

We first identified miRNAs and their expression pattern in B-deficient *C. sinensis* roots by Illumina sequencing. A total of 538 known miRNAs and of 108 novel miRNAs was identified from control and B-deficient roots. In B-deficient roots, 52 (40 known and 12 novel) up-regulated and 82 (72 known and 10 novel) down-regulated miRNAs were isolated. This demonstrates remarkable metabolic flexibility of *C. sinensis* roots, which might contribute to the tolerance of roots to B-deficiency. A model for the possible roles of miRNAs in the tolerance of roots to B-deficiency was proposed through the integration of the present results and available data in the literature (Figure 6). miRNAs might regulate the adaptations of *C. sinensis* roots to B-deficiency through following several aspects: (*a*) activation of the defense response, ROS signaling and scavenging due to increased expression of *miR474* and decreased expression of *miR782* and *miR843*; (*b*) increasing the number of lateral roots (miR5023) and maintaining a certain phenotype favorable for the adaptive response to B-deficiency (miR394); (*c*) enhancing cell transport by decreasing the accumulation of *miR830*, *miR5266* and *miR3465*; (*c*) improving osmoprotection (miR474) and regulating other metabolic reactions (miR5023 and miR821). In addition, both *miR472* and *miR2118* expression increased in B-deficient *C. sinensis* roots, thus decreasing the expression of their target genes, which are involved in disease resistance, and hence, the disease resistance of roots. Therefore, the discovery and characterization of these B-deficiency-responsive miRNAs will help us to elucidate the molecular mechanisms involved in the tolerance of plants to B-deficiency. Although the absolute conditions without B created under pot conditions do not exist in field, because there is always certain level of B supply in field conditions even under highly B-deficient conditions, pot results should stand ture under field conditions, because typical B-deficient symptoms: corky split veins of *Citrus* leaves usually occur in the sand culture and in field conditions [33,38,93].

## Methods

### Plant culture and B treatments

Plant culture and B treatments were performed according to Yang et al. [37]. Briefly, 5-week-old seedlings of ‘Xuegan’ [*Citrus sinensis* (L.) Osbeck] were transplanted into 6 L pots containing fine river sand. Ten weeks after transplanting, each pot was supplied every other day with B-deficient (0 μM H_3_BO_3_) or -sufficient (10 μM H_3_BO_3_, control) nutrient solution for 15 weeks. There were 10 replications per B treatment with 2 pots in a completely randomized design. Plants grown in the absence of B first developed in the apex and in the actively growing leaves because B is phloem immobile in *Citrus*. B-deficient symptoms in mature leaves were characterized by enlargement, splitting and corking of leaf veins [33,34]. At the end of the experiment, approx. 5-mm-long root apices were frozen immediately in liquid N_2_ after being excised from the seedlings. Root samples were stored at −80°C until extraction.

### Plant DW, root and leaf B

At the end of the experiment, seven plants per treatment from different replications were harvested. The plants were divided into their separate parts (roots and shoots). The plant material was then dried at 80°C for 48 h and the DW measured. B concentration in roots and leaves was assayed by ICP emission spectrometry after microwave digestion with HNO_3_[37].

### Root flavonoids, anthocyanin, proline, proline dehydrogenase and glutamate dehydrogenase

Flavonoid and anthocyanin were assayed as described by Krizek et al. [94] and Wagner [95], respectively. Free proline and proline dehydrogenase (PDH) were assayed according to Bates et al. [96] and Veeranjaneyulu and Kumari [97], respectively. The amination (NADH) and deamination (NAD) reactions of glutamate dehydrogenase (GDH) were assayed according to Loyola-Vargas and de Jimenez [98].

### Isolation of sRNAs, library construction and high-throughput sequencing

About 0.1 g mixed frozen B-sufficient and -deficient roots from five replictations were used to extract RNA. Total RNA was extracted from frozen roots using TRIzol reagent (Invitrogen, Carlsbad, CA) following manufacturer’s instructions. Two sRNA libraries were constructed according to Wang et al. [62]. Briefly, sRNAs were isolated from the total RNA by size fractionation with 15% Tris-borate-EDTA urea polyacrylamide gel (TBU). Then the sRNAs were ligated with 5' and 3' adaptor by T4 RNA ligase after being dephosphorylated by alkaline phosphatase. The adaptor-ligated sRNAs were transcribed to single-stranded cDNA using Superscript II reverse transcriptase (Invitrogen). Thereafter, the single-stranded cDNA was used as templates for double-stranded synthesis by PCR amplification using the primer designed according to the adapter sequence. The obtained PCR products were sequenced on a Solexa sequencer (Illumina) at the Beijing Genomics Institute (BGI), Shenzhen, China.

### sRNA annotation and miRNA identification

The raw reads obtained from the Solexa sequencing were cleaned by removing adaptors, low quality tags as well as contaminant reads including those reads with 5´-primer contaminants, reads without 3´-primer, reads with poly A, reads without the insert tag, and reads with length less than 18 nt. We use software developed by the BGI to deal with the data from the Solexa sequencing. The clean reads were then used to analyze length distribution and common/specific sequences. Thereafter, the clear reads were mapped to *Citrus clementina* genome (JGI version 0.9, http://www.phytozome.org/clementine.php, 35976 sequences) using SOAP, only perfectly mapped sequences were retained and analyzed further. rRNAs, tRNAs, snRNAs and snoRNAs were removed from the sRNAs sequences through BLASTn search using NCBI Genebank database (http://www.ncbi.nlm.nih.gov/blast/Blast.cgi/) and Rfam (10.1) database (http://www.sanger.ac.uk/resources/databases/rfam.html) (*e* = 0.01). The remaining sequences were aligned with known plant miRNAs from miRBase 18 (http://www.mirbase.org/). Only the perfectly matched sequences were considered to be conserved miRNAs. Reads that were not annotated were used to predict novel miRNAs using a prediction software Mireap (http://sourceforge.net/projects/mireap/), which was developed by the BGI, by exploring the secondary structure, the Dicer cleavage site and the minimum free energy of the unannotated small RNA tags which could be mapped to genome. Parameters were set as follows: minimal miRNA sequence length (18), maximal miRNA sequence length (25), minimal miRNA reference sequence length (20), maximal miRNA reference sequence length (23), maximal copy number of miRNAs on reference (20), maximal free energy allowed for a miRNA precursor (−18 kcal/mol), maximal space between miRNA and miRNA* (300), minimal base pairs of miRNA and miRNA* (16), maximal bulge of miRNA and miRNA* (4), maximal asymmetry of miRNA/miRNA* duplex (4) and flank sequence length of miRNA precursor (20).

### Differential expression analysis of miRNAs under B-deficiency

Both the fold change between B-deficiency and -sufficiency (control) and the *P*-value were calculated from the normalized expression of transcript per million (TPM) [62]. Normalized expression was calculated by the following formula: Normalized expression = Actual miRNA count/Total count of clean reads*1,000,000. The fold change between B-deficiency and control was calculated as: Fold-change = log_2_ (B-deficiency/Control). The *p*-value was calculated by the following formula:

$$p\left(x|y\right)=\left(\frac{N_{2}}{N_{1}}\right){\frac{y\;\left(x+y\right)\underset{˙}{l}}{x\underset{˙}{l}y\underset{˙}{l}\left(1+\frac{N_{2}}{N_{1}}\right)\left(x+y+1\right)}}_{D\left(y\geq y_{\max}|x\right)=\sum_{y\geq y_{\max}}^{\infty }p\left(y|x\right)}^{C\left(y\leq y_{\min}|x\right)=\sum_{y=0}^{y\leq y_{\min}}p\left(y|x\right)}$$

A 1.5-fold cut-off was set to determine up-regulated and down-regulated miRNAs in addition to a *P*-value of less than 0.01.

### Target prediction of miRNAs

Target prediction of miRNAs was performed by RNAhybrid based on rules suggested by Allen et al. [14] and Schwab et al. [52].

### Functions of the potential targets of the differentially expressed miRNAs

To reveal the functions of the predicted target genes of the differentially expressed miRNAs, all targets were mapped to GO terms in the database (http://www.geneontology.org/), and calculated gene numbers for each term. The GO results were expressed as three categories: cellular component, molecular function, biological process [99].

### Validation of miRNA expression by real time quantitative reverse transcription PCR (qRT-PCR)

Total RNA was extracted from B-sufficient and -deficient roots as described above. About 2.0 μg total RNA was polyadenylated with ATP by poly(A) polymerase and reverse-transcribed with poly(T) adapter primer by PrimeScript® RTase at 42°C according to manufacturer’s instruction (Takara, Japan). Add enough RNA-free dH_2_O to bring to a final volume of each tube to 100 μL and pipet 1 μL aliquot to the next qRT-PCR. Twenty-six miRNAs were selected to perform qRT-PCR to validate the miRNA expression obtained from the high-throughput sequencing. miRNA special (forward) primers were designed according to the miRNA sequence but excluded the last two to five nucleotides at 3' end of the miRNA. A 5' extension of three to five nucleotides, which was chosen randomly and relatively GC-rich, was added to each forward primer to increase the melting temperature [100]. All the primers were assigned to Primer Software Version 5.0 (PREMIER Biosoft International, USA) to assess their quality. All the primers used were listed in Additional file 8. For qRT-PCR, 20 μL reaction solution contained 10 μL ready-to-use SYBR® Premix Ex TaqTM II (Takara, Japan), 0.8 μL 10 μM miRNA forward primer, 0.8 μL 10 μM Uni-miR qPCR primer, 2 μL cDNA template and 6.4 μL dH_2_O. qRT-PCR was performed with a Mastercycler Ep Realplex System (Eppendorf, Hamburg, Germany) using *actin* (AEK97331.1) as internal control. The cycling conditions were 60 s at 95°C, followed by 40 cycles of 95°C for 10 s, 60°C for 30 s. Samples for qRT-PCR were run in at least three biological replicates with three technical replicates. Relative miRNA expression was calculated using ddCt algorithm. For the normalization of miRNA expression, *actin* gene was used as an internal standard and the roots from control plants were used as reference sample, which was set to 1.

### qRT-PCR analysis of miRNA target gene expression

Total RNA was extracted from frozen B-sufficient and -deficient roots using TRIzol reagent (Invitrogen, Carlsbad, CA) following manufacturer’s instructions. The sequences of the F and R primers used were given in Additional file 9. qRT-PCR analysis of miRNA target gene expression was performed using a Mastercycler Ep Realplex System (Eppendorf, Hamburg, Germany) as previously described by Yang et al. [37].

### Experimental design and statistical analysis

There were 20 pot seedlings per treatment in a completely randomized design. Experiments were performed with 4–7 replicates. Differences among treatments were separated by the least significant difference (LSD) test at *P* < 0.05 level.

### Availability of supporting data

“The data set supporting the results of this article are available in the Gene Expression Omnibus repository under accession no GSE57016 (http://www.ncbi.nlm.nih.gov/geo/query/acc.cgi?acc=GSE57016)”. The mature miRNA and precursor sequences will be submitted to miRBase registry and assigned final names after final acceptance of the manuscript.

## Competing interests

The authors declare that they have no competing interests.

## Authors’ contributions

YBL carried out most of the experiments and drafted the manuscript. LTY participated in the design of the study and coordination. YPQ participated in the design of the study. YL directed the study. ZL and YBC carried out the cultivation of seedlings. ZRH performed the statistical analysis. LSC designed and directed the study and revised the manuscript. All authors have read and approved the final manuscript.

## Supplementary Material

Additional file 1 **Length distribution of small RNAs from control and B-deficient roots of *Citrus sinensis *** **seedlings.** Click here for file

Additional file 2 **List of known miRNAs in *Citrus sinensis *** **roots.** Click here for file

Additional file 3 **List of known miRNAs in *Citrus sinensis *** **roots after removing these miRNAs with normalized read-count less than 10 TPM in two miRNA libraries constructed from control and B-deficient roots.** Click here for file

Additional file 4 **List of novel miRNAs in *Citrus sinensis *** **roots.** Click here for file

Additional file 5 **List of novel miRNAs in *Citrus sinensis *** **roots after removing these miRNAs with normalized read-count less than 10 TPM in two miRNA libraries constructed from control and B-deficient roots.** Click here for file

Additional file 6 **List of target genes for parts of known miRNAs in *Citrus sinensis *** **roots.** Click here for file

Additional file 7 **List of target genes for parts of novel miRNAs in *Citrus sinensis *** **roots.** Click here for file

Additional file 8 Primer sequences for qRT-PCR expression analysis of miRNAs. Click here for file

Additional file 9 Specific primer pairs used for qRT-PCR expression analysis of selected miRNA target genes. Click here for file
