# Supplementary material for: Identification of boron-deficiency-responsive microRNAs in Citrus sinensis roots by Illumina sequencing
Source: BMC Plant Biol. 2014 May 7;14:123. doi: 10.1186/1471-2229-14-123 (PMC4041134; doi:10.1186/1471-2229-14-123)
Supplement: Additional file 1 — Length distribution of small RNAs from control and B-deficient roots of Citrus sinensis seedlings. [file 1471-2229-14-123-S1.doc]

**Additional file 1:** **Length distribution of small RNAs from control and B-deficient roots of *Citrus sinensis* seedlings.**
