# Supplementary material for: Identification of boron-deficiency-responsive microRNAs in Citrus sinensis roots by Illumina sequencing
Source: BMC Plant Biol. 2014 May 7;14:123. doi: 10.1186/1471-2229-14-123 (PMC4041134; doi:10.1186/1471-2229-14-123)
Supplement: Additional file 6 — List of target genes for parts of known miRNAs in Citrus sinensis roots. [file 1471-2229-14-123-S6.doc]

**Additional file 6: List of target genes for parts of known miRNAs in *Citrus sinensis*** roots

| *miRNA* | *Assession* | *Homology* | *Target genes* |
| --- | --- | --- | --- |
| miR1039 | clementine0.9_005207m|PACid:19252019  clementine0.9_000376m|PACid:19256951  clementine0.9_001108m|PACid:19269426  clementine0.9_001167m|PACid:19269832  clementine0.9_020605m|PACid:19275271  clementine0.9_000074m|PACid:19278562  clementine0.9_012851m|PACid:19278553  clementine0.9_001853m|PACid:19280618  clementine0.9_013970m|PACid:19287116 | AT4G31820.1  AT2G25320.1  AT5G53890.1  AT4G24200.1  AT5G62390.1  AT3G54280.1  AT1G64970.1  AT3G25500.1  AT3G56680.1 | Phototropic-responsive NPH3 family protein  TRAF-like family protein  Phytosylfokine-alpha receptor 2  Transcription elongation factor (TFIIS) family protein  BCL-2-associated athanogene 7  DNA binding; ATP binding; nucleic acid binding; binding; helicases; ATP binding; DNA binding; helicases  Gamma-tocopherol methyltransferase  Formin homology 1  Single-stranded nucleic acid binding R3H protein |
| miR1074 | clementine0.9_033036m|PACid:19260189  clementine0.9_029301m|PACid:19275693 | AT1G16790.1  AT4G23340.1 | Rbosomal protein-related  2-Oxoglutarate (2OG) and Fe(II)-dependent oxygenase superfamily protein |
| miR1105 | clementine0.9_001123m|PACid:19258394  clementine0.9_006354m|PACid:19265027  clementine0.9_000028m|PACid:19273862  clementine0.9_035383m|PACid:19278859  clementine0.9_027998m|PACid:19279965  clementine0.9_010713m|PACid:19283111 | AT5G15400.1  AT4G00550.1  AT2G26890.1  AT1G16380.1  AT4G17600.1  AT3G20500.1 | U-box domain-containing protein  Digalactosyl diacylglycerol deficient 2  DNAJ heat shock N-terminal domain-containing protein  Cation/hydrogen exchanger family protein  Chlorophyll A-B binding family protein  Purple acid phosphatase 18 |
| miR1132 | clementine0.9_006465m|PACid:19251938  clementine0.9_019208m|PACid:19256955  clementine0.9_019769m|PACid:19256956  clementine0.9_005892m|PACid:19257613  clementine0.9_023646m|PACid:19262238  clementine0.9_013738m|PACid:19265293  clementine0.9_027139m|PACid:19265435  clementine0.9_009204m|PACid:19265895  clementine0.9_032144m|PACid:19273385  clementine0.9_010999m|PACid:19274106  clementine0.9_009929m|PACid:19281229  clementine0.9_013939m|PACid:19284731  clementine0.9_005601m|PACid:19286001 | AT5G10650.2  AT4G32280.1  AT1G04250.1  AT3G22960.1  AT5G56260.1  AT3G30340.1  AT1G14450.1  AT3G48730.1  AT1G80000.1  AT4G13840.1  AT1G32440.1  AT5G49610.1  AT1G08350.2 | RING/U-box superfamily protein  Indole-3-acetic acid inducible 29  AUX/IAA transcriptional regulator family protein  Pyruvate kinase family protein  Ribonuclease E inhibitor RraA/Dimethylmenaquinone methyltransferase  Nodulin MtN21 /EamA-like transporter family protein  NADH dehydrogenase (ubiquinone)s  Glutamate-1-semialdehyde 2,1-aminomutase 2  CASC3/Barentsz eIF4AIII binding  HXXXD-type acyl-transferase family protein  Plastidial pyruvate kinase 3  F-box family protein  Endomembrane protein 70 protein family |
| miR1160 | clementine0.9_032551m|PACid:19254409  clementine0.9_000507m|PACid:19256480  clementine0.9_011117m|PACid:19266000  clementine0.9_020496m|PACid:19274665  clementine0.9_009770m|PACid:19278649  clementine0.9_033542m|PACid:19278761  clementine0.9_027659m|PACid:19279356  clementine0.9_019781m|PACid:19281915  clementine0.9_032996m|PACid:19282948 | AT1G09890.1  AT5G24710.1  AT4G29310.1  AT5G59470.1  AT1G09540.1  AT5G20150.1  AT5G06900.1  AT5G59380.1  AT2G21630.1 | Rhamnogalacturonate lyase family protein  Transducin/WD40 repeat-like superfamily protein  Protein of unknown function (DUF1005)  Mannose-P-dolichol utilization defect 1 protein  Myb domain protein 61  SPX domain gene 1  Cytochrome P450, family 93, subfamily D, polypeptide 1  Methyl-CPG-binding domain 6  Sec23/Sec24 protein transport family protein |
| miR1166 | clementine0.9_035572m|PACid:19256024  clementine0.9_028564m|PACid:19261657 | AT1G23740.1  AT3G15010.2 | Oxidoreductase, zinc-binding dehydrogenase family protein  RNA-binding (RRM/RBD/RNP motifs) family protein |
| miR1439 | clementine0.9_006314m|PACid:19252338  clementine0.9_017525m|PACid:19254197  clementine0.9_031969m|PACid:19255037  clementine0.9_006628m|PACid:19255590  clementine0.9_001099m|PACid:19255971  clementine0.9_034961m|PACid:19255906  clementine0.9_035152m|PACid:19255894  clementine0.9_003465m|PACid:19257850  clementine0.9_000672m|PACid:19259098  clementine0.9_019331m|PACid:19261025  clementine0.9_036030m|PACid:19261344  clementine0.9_004573m|PACid:19262431  clementine0.9_026007m|PACid:19261834  clementine0.9_000096m|PACid:19265346  clementine0.9_011884m|PACid:19264017  clementine0.9_010623m|PACid:19266768  clementine0.9_012900m|PACid:19266759  clementine0.9_000334m|PACid:19268181  clementine0.9_008186m|PACid:19269132  clementine0.9_032150m|PACid:19269596  clementine0.9_031173m|PACid:19270206  clementine0.9_003927m|PACid:19270372  clementine0.9_028590m|PACid:19270382  clementine0.9_015439m|PACid:19273718  clementine0.9_001794m|PACid:19272916  clementine0.9_006256m|PACid:19272697  clementine0.9_009353m|PACid:19274527  clementine0.9_013392m|PACid:19275807  clementine0.9_031646m|PACid:19277960  clementine0.9_011111m|PACid:19278477  clementine0.9_015060m|PACid:19278986  clementine0.9_008180m|PACid:19279098  clementine0.9_011895m|PACid:19279810  clementine0.9_011902m|PACid:19279811  clementine0.9_035167m|PACid:19279942  clementine0.9_004840m|PACid:19281048  clementine0.9_013818m|PACid:19281547  clementine0.9_006895m|PACid:19281645  clementine0.9_031156m|PACid:19281647  clementine0.9_001272m|PACid:19282096  clementine0.9_017772m|PACid:19282623  clementine0.9_028907m|PACid:19282242  clementine0.9_027986m|PACid:19282756  clementine0.9_000387m|PACid:19283443  clementine0.9_014436m|PACid:19283510  clementine0.9_022783m|PACid:19284746  clementine0.9_001891m|PACid:19286314  clementine0.9_015549m|PACid:19287065 | AT4G03390.1  AT1G54870.1  AT5G02700.1  AT5G48385.1  AT3G11010.1  AT2G15080.1  AT5G27060.1  AT5G23050.1  AT1G59820.1  AT1G06330.1  AT1G74170.1  AT5G55860.1  AT4G31170.1  AT4G00800.1  AT3G61590.2  AT2G38510.1  AT5G04110.1  AT3G14460.1  AT5G66680.1  AT4G21700.1  AT1G75130.1  AT1G61010.2  AT4G35790.2  AT5G19930.1  AT1G22620.1  AT1G77250.1  AT3G19270.1  AT4G33410.1  AT1G74190.1  AT2G38670.1  AT4G10000.2  AT2G30880.1  AT3G59360.2  AT3G59360.1  AT5G50990.1  AT1G71020.1  AT1G08450.1  AT2G30320.1  AT2G38470.1  AT5G22750.1  AT1G09760.1  AT5G57620.1  AT1G19260.1  AT3G52140.1  AT2G03060.2  AT2G21600.1  AT5G11720.1  AT5G37930.1 | STRUBBELIG-receptor family 3  NAD(P)-binding Rossmann-fold superfamily protein  F-box/RNI-like superfamily protein  FRIGIDA-like protein  Receptor like protein 34  Receptor like protein 19  Receptor like protein 53  Acyl-activating enzyme 17  Aminophospholipid ATPase 3  Heavy metal transport/detoxification superfamily protein  Receptor like protein 13  Plant protein of unknown function (DUF827)  Protein kinase superfamily protein  Transducin family protein / WD-40 repeat family protein  Galactose oxidase/kelch repeat superfamily protein  MATE efflux family protein  DNA GYRASE B3  LRR and NB-ARC domains-containing disease resistance protein  Dolichyl-diphosphooligosaccharide-protein glycosyltransferase 48kDa subunit family protein  Protein of unknown function (DUF2921)  Cytochrome P450, family 721, subfamily A, polypeptide 1  Cleavage and polyadenylation specificity factor 73-I  Phospholipase D delta  Protein of unknown function DUF92, transmembrane  Phosphoinositide phosphatase family protein  RING/FYVE/PHD-type zinc finger family protein  Cytochrome P450, family 707, subfamily A, polypeptide 4  SIGNAL PEPTIDE PEPTIDASE-LIKE 1  Receptor like protein 15  Phosphorylethanolamine cytidylyltransferase 1  Thioredoxin family protein  Pleckstrin homology (PH) domain-containing protein  UDP-galactose transporter 6  UDP-galactose transporter 6  Tetratricopeptide repeat (TPR)-like superfamily protein  ARM repeat superfamily protein  Calreticulin 3  Pseudouridine synthase family protein  WRKY DNA-binding protein 33  DNA/RNA helicase protein  U2 small nuclear ribonucleoprotein A  Myb domain protein 36  TTF-type zinc finger protein with HAT dimerisation domain  Tetratricopeptide repeat (TPR)-containing protein  AGAMOUS-like 30  Endoplasmatic reticulum retrieval protein 1B  Glycosyl hydrolases family 31 protein  Protein with RING/U-box and TRAF-like domains |
| miR1446 | clementine0.9_009789m|PACid:19251454  clementine0.9_014070m|PACid:19251304  clementine0.9_010916m|PACid:19252763  clementine0.9_000559m|PACid:19253748  clementine0.9_000808m|PACid:19253354  clementine0.9_005886m|PACid:19254086  clementine0.9_007940m|PACid:19254486  clementine0.9_008049m|PACid:19254311  clementine0.9_012380m|PACid:19253071  clementine0.9_016619m|PACid:19253304  clementine0.9_001520m|PACid:19255096  clementine0.9_001057m|PACid:19256298  clementine0.9_002803m|PACid:19256134  clementine0.9_001495m|PACid:19258364  clementine0.9_022364m|PACid:19258749  clementine0.9_000569m|PACid:19258946  clementine0.9_008857m|PACid:19259453  clementine0.9_031455m|PACid:19259065  clementine0.9_028686m|PACid:19262485  clementine0.9_000196m|PACid:19265021  clementine0.9_000254m|PACid:19263791  clementine0.9_000450m|PACid:19265308  clementine0.9_029313m|PACid:19265371  clementine0.9_005739m|PACid:19266195  clementine0.9_019579m|PACid:19266319  clementine0.9_001508m|PACid:19267417  clementine0.9_004412m|PACid:19267097  clementine0.9_009982m|PACid:19266595  clementine0.9_023656m|PACid:19266734  clementine0.9_035211m|PACid:19268576  clementine0.9_002714m|PACid:19269902  clementine0.9_020450m|PACid:19270860  clementine0.9_034356m|PACid:19271149  clementine0.9_002007m|PACid:19277439  clementine0.9_006001m|PACid:19276494  clementine0.9_007912m|PACid:19277337  clementine0.9_032808m|PACid:19278951  clementine0.9_007920m|PACid:19279021  clementine0.9_031010m|PACid:19279343  clementine0.9_033863m|PACid:19279776  clementine0.9_012307m|PACid:19280476  clementine0.9_004243m|PACid:19280580  clementine0.9_028125m|PACid:19282910  clementine0.9_003203m|PACid:19286100  clementine0.9_035063m|PACid:19286006  clementine0.9_010246m|PACid:19287049  clementine0.9_011766m|PACid:19286808  clementine0.9_015366m|PACid:19286991 | AT5G65720.1  AT2G22780.1  AT5G38200.1  AT5G27240.1  AT1G27750.1  AT2G32910.1  AT5G42020.1  AT1G22360.1  AT3G02630.1  AT2G42730.1  AT1G79830.1  AT3G27870.1  AT2G27100.1  AT5G15450.1  AT1G08770.1  AT3G04340.1  AT1G14920.1  AT1G67230.1  AT5G36110.1  AT3G62900.1  AT4G00060.1  AT2G48160.1  AT4G10250.1  AT5G09880.1  AT1G76010.1  AT5G01030.2  AT1G26620.1  AT4G22580.1  AT1G12440.2  AT3G47570.1  AT1G45160.2  AT1G55790.1  AT1G78580.1  AT5G47040.1  AT2G46500.1  AT1G14920.1  AT1G33770.1  AT3G59110.1  AT1G56150.1  AT5G19610.1  AT1G61680.1  AT1G06290.1  AT5G27740.1  AT1G11660.1  AT4G19650.1  AT3G56900.1  AT1G77180.2  AT1G71080.1 | Nitrogen fixation S (NIFS)-like 1  Peroxisomal NAD-malate dehydrogenase 1  Class I glutamine amidotransferase-like superfamily protein  DNAJ heat shock N-terminal domain-containing protein  Nucleic acid binding  DCD (Development and Cell Death) domain protein  Heat shock protein 70 (Hsp 70) family protein  UDP-glucosyl transferase 85A2  Plant stearoyl-acyl-carrier-protein desaturase family protein  F-box family protein  Golgin candidate 5  ATPase E1-E2 type family protein / haloacid dehalogenase-like hydrolase family protein  C2H2 zinc-finger protein SERRATE (SE)  Casein lytic proteinase B3  Prenylated RAB acceptor 1.E  FtsH extracellular protease family  GRAS family transcription factor family protein  Little nuclei1  cytochrome P450, family 716, subfamily A, polypeptide 1  CW-type Zinc Finger  Nucleotidyltransferase family protein  Tudor/PWWP/MBT domain-containing protein  HSP20-like chaperones superfamily protein  Splicing factor, CC1-like  Alba DNA/RNA-binding protein  Protein of unknown function (DUF3527)  Plant protein of unknown function (DUF863)  Exostosin family protein  A20/AN1-like zinc finger family protein  Leucine-rich repeat protein kinase family protein  Protein kinase superfamily protein  Domain of unknown function (DUF2431)  Trehalose-6-phosphate synthase  Lon protease 2  Phosphoinositide 4-kinase gamma 4  GRAS family transcription factor family protein  Protein kinase superfamily protein  Protein kinase superfamily protein  SAUR-like auxin-responsive protein family  GNOM-like 2  Terpene synthase 14  Acyl-CoA oxidase 3  ATPase family associated with various cellular activities (AAA)  Heat shock protein 70 (Hsp 70) family protein  Mitochondrial transcription termination factor family protein  Transducin/WD40 repeat-like superfamily protein  Chromatin protein family  RNA polymerase II transcription elongation factor |
| miR1448 | clementine0.9_000636m|PACid:19254755  clementine0.9_007971m|PACid:19253613  clementine0.9_035618m|PACid:19255262  clementine0.9_000530m|PACid:19256469  clementine0.9_013371m|PACid:19259714  clementine0.9_000447m|PACid:19267583  clementine0.9_008191m|PACid:19272780  clementine0.9_026415m|PACid:19278691  clementine0.9_030249m|PACid:19278775 | AT4G27220.1  AT1G56570.1  AT5G63020.1  AT3G14460.1  AT1G26520.1  AT3G14470.1  AT1G35670.1  AT1G12290.1  AT1G12280.1 | NB-ARC domain-containing disease resistance protein  Tetratricopeptide repeat (TPR)-like superfamily protein  Disease resistance protein (CC-NBS-LRR class) family  LRR and NB-ARC domains-containing disease resistance protein  Cobalamin biosynthesis CobW-like protein  NB-ARC domain-containing disease resistance protein  Calcium-dependent protein kinase 2  Disease resistance protein (CC-NBS-LRR class) family  LRR and NB-ARC domains-containing disease resistance protein |
| miR1511 | clementine0.9_009820m|PACid:19262426  clementine0.9_010543m|PACid:19251759  clementine0.9_000362m|PACid:19254873  clementine0.9_005360m|PACid:19260067  clementine0.9_017002m|PACid:19260007  clementine0.9_020825m|PACid:19262370  clementine0.9_002560m|PACid:19264079  clementine0.9_012226m|PACid:19264235  clementine0.9_024643m|PACid:19267538  clementine0.9_007078m|PACid:19272129  clementine0.9_007573m|PACid:19271806  clementine0.9_019660m|PACid:19273245  clementine0.9_019985m|PACid:19273552  clementine0.9_003582m|PACid:19276983  clementine0.9_023956m|PACid:19280797  clementine0.9_011625m|PACid:19283400  clementine0.9_008803m|PACid:19285008 | AT2G30490.1  AT2G19260.1  AT5G56890.1  AT3G26600.1  AT1G10710.2  AT4G26470.1  AT2G20320.1  AT5G27740.1  AT4G33050.4  AT1G49960.1  AT4G34131.1  AT5G11640.1  AT3G13580.2  AT4G23990.1  AT1G19910.1  AT1G14180.1  AT4G36250.1 | Cinnamate-4-hydroxylase  RING/FYVE/PHD zinc finger superfamily protein  Protein kinase superfamily protein  Armadillo repeat only 4  Poor homologous synapsis 1  Calcium-binding EF-hand family protein  DENN (AEX-3) domain-containing protein  ATPase family associate d with various cellular activities (AAA)  Calmodulin-binding family protein  Xanthine/uracil permease family protein  UDP-glucosyl transferase 73B3  Thioredoxin superfamily protein  Ribosomal protein L30/L7 family protein  Cellulose synthase like G3  ATPase, F0/V0 complex, subunit C protein  RING/U-box superfamily protein  Aldehyde dehydrogenase 3F1 |
| miR1523 | clementine0.9_013353m|PACid:19252451  clementine0.9_005176m|PACid:19252976  clementine0.9_024962m|PACid:19253042  clementine0.9_020478m|PACid:19256143  clementine0.9_033308m|PACid:19255986  clementine0.9_001290m|PACid:19257400  clementine0.9_000065m|PACid:19259170  clementine0.9_015468m|PACid:19259994  clementine0.9_017632m|PACid:19263026  clementine0.9_028903m|PACid:19262045  clementine0.9_009243m|PACid:19265635  clementine0.9_000014m|PACid:19267492  clementine0.9_006817m|PACid:19268167  clementine0.9_036038m|PACid:19268134  clementine0.9_000034m|PACid:19272011  clementine0.9_003975m|PACid:19273632  clementine0.9_005249m|PACid:19273633  clementine0.9_006020m|PACid:19273555  clementine0.9_009336m|PACid:19273087  clementine0.9_002381m|PACid:19275635  clementine0.9_001778m|PACid:19277596  clementine0.9_018848m|PACid:19279278  clementine0.9_007883m|PACid:19279546  clementine0.9_008678m|PACid:19279547  clementine0.9_012740m|PACid:19281131  clementine0.9_029914m|PACid:19281977  clementine0.9_009207m|PACid:19283826  clementine0.9_008811m|PACid:19284732  clementine0.9_018364m|PACid:19285318  clementine0.9_001103m|PACid:19286592  clementine0.9_006018m|PACid:19287071 | AT1G06620.1  AT5G48380.1  AT3G49760.1  AT3G28050.1  AT4G28010.1  AT1G68940.1  AT3G01460.1  AT5G47000.1  AT4G30993.2  AT2G17030.1  AT3G13620.1  AT5G24740.1  AT2G03220.1  AT4G13810.1  AT1G50030.1  AT4G32850.9  AT4G32850.5  AT2G25850.4  AT1G43190.1  AT5G51430.1  AT1G17680.1  AT2G42940.1  AT4G13710.2  AT1G04680.1  AT1G06620.1  AT1G76390.2  AT5G01240.1  AT2G18570.1  AT2G46780.1  AT5G04480.1  AT2G41140.1 | 2-Oxoglutarate (2OG) and Fe(II)-dependent oxygenase superfamily protein  BAK1-interacting receptor-like kinase 1  Basic leucine-zipper 5  Nodulin MtN21 /EamA-like transporter family protein  Tetratricopeptide repeat (TPR)-like superfamily protein  Armadillo/beta-catenin-like repeat family protein  Methyl-CPG-binding domain 9  Peroxidase superfamily protein  Calcineurin-like metallo-phosphoesterase superfamily protein  F-box family protein with a domain of unknown function (DUF295)  Amino acid permease family protein  Protein of unknown function (DUF1162)  Fucosyltransferase 1  Receptor like protein 47  Target of rapamycin  Nuclear poly(a) polymerase  Nuclear poly(a) polymerase  Poly(A) polymerase 2  Polypyrimidine tract-binding protein 3  Conserved oligomeric Golgi complex component-related / COG complex component-related  Tetratricopeptide repeat (TPR)-containing protein  Predicted AT-hook DNA-binding family protein  Pectin lyase-like superfamily protein  Pectin lyase-like superfamily protein  2-Oxoglutarate (2OG) and Fe(II)-dependent oxygenase superfamily protein  ARM repeat superfamily protein  Like AUXIN RESISTANT 1  UDP-Glycosyltransferase superfamily protein  RNA-binding (RRM/RBD/RNP motifs) family protein  UDP-Glycosyltransferase superfamily protein  CDPK-related kinase 1 |
| miR157 | clementine0.9_022829m|PACid:19252333  clementine0.9_023574m|PACid:19252334  clementine0.9_008011m|PACid:19259369  clementine0.9_008959m|PACid:19258968  clementine0.9_008954m|PACid:19258969  clementine0.9_016441m|PACid:19260022  clementine0.9_012563m|PACid:19264657  clementine0.9_002919m|PACid:19270659  clementine0.9_024862m|PACid:19271517 | AT1G53160.2  AT1G53160.1  AT1G69170.1  AT5G43270.1  AT5G43270.2  AT5G50570.1  AT2G42200.1  AT5G45650.1  AT2G33810.1 | Squamosa promoter binding protein-like 4  Squamosa promoter binding protein-like 4  Squamosa promoter-binding protein-like (SBP domain) transcription factor family protein  Squamosa promoter binding protein-like 2  Squamosa promoter binding protein-like 2  Squamosa promoter-binding protein-like (SBP domain) transcription factor family protein  Squamosa promoter binding protein-like 9  Subtilase family protein  Squamosa promoter binding protein-like 3 |
| miR158 | clementine0.9_003267m|PACid:19252391  clementine0.9_010359m|PACid:19256106  clementine0.9_031647m|PACid:19256610  clementine0.9_034573m|PACid:19256451  clementine0.9_003549m|PACid:19260547  clementine0.9_007051m|PACid:19260889  clementine0.9_005473m|PACid:19263371  clementine0.9_008444m|PACid:19265797  clementine0.9_016766m|PACid:19266772  clementine0.9_028365m|PACid:19269373  clementine0.9_000068m|PACid:19272975  clementine0.9_010398m|PACid:19274995  clementine0.9_029429m|PACid:19275671  clementine0.9_012376m|PACid:19276938  clementine0.9_012527m|PACid:19279015  clementine0.9_000036m|PACid:19279439  clementine0.9_007734m|PACid:19279748  clementine0.9_001517m|PACid:19280274  clementine0.9_035912m|PACid:19281238  clementine0.9_006854m|PACid:19281668  clementine0.9_002134m|PACid:19282380  clementine0.9_007666m|PACid:19284165  clementine0.9_033926m|PACid:19286241 | AT4G03560.1  AT2G20570.1  AT3G14470.1  AT3G14460.1  AT4G15560.1  AT5G02320.1  AT2G38840.1  AT3G13560.3  AT3G51680.1  AT5G27100.1  AT1G21580.1  AT5G51760.1  AT1G30760.1  AT1G63930.1  AT5G50890.1  AT4G15180.1  AT3G18830.1  AT3G14470.1  AT4G39340.1  AT2G30300.1  AT1G07990.1  AT5G05260.1  AT1G34575.1 | Two-pore channel 1  GBF\'s pro-rich region-interacting factor 1  NB-ARC domain-containing disease resistance protein  LRR and NB-ARC domains-containing disease resistance protein  Deoxyxylulose-5-phosphate synthase  Myb domain protein 3r-5  Guanylate-binding family protein  O-Glycosyl hydrolases family 17 protein  NAD(P)-binding Rossmann-fold superfamily protein  glutamate receptor 2.1  Zinc finger C-x8-C-x5-C-x3-H type family protein  Protein phosphatase 2C family protein  FAD-binding Berberine family protein  From the Czech \'roh\' meaning \'corner\'  Alpha/beta-Hydrolases superfamily protein  SET domain protein 2  polyol/monosaccharide transporter 5  NB-ARC domain-containing disease resistance protein  Protein of unknown function (DUF1278)  Major facilitator superfamily protein  SIT4 phosphatase-associated family protein  cytochrome p450 79a2  FAD-binding Berberine family protein |
| miR163 | clementine0.9_009522m|PACid:19264899  clementine0.9_001610m|PACid:19261259  clementine0.9_034528m|PACid:19281322 | AT4G38040.1  AT3G14470.1  AT3G50950.2 | Exostosin family protein  NB-ARC domain-containing disease resistance protein  HOPZ-ACTIVATED RESISTANCE 1 |
| miR165 | clementine0.9_002420m|PACid:19255038  clementine0.9_017141m|PACid:19255649  clementine0.9_011723m|PACid:19260003  clementine0.9_014167m|PACid:19260476  clementine0.9_002566m|PACid:19265474  clementine0.9_005884m|PACid:19266903  clementine0.9_002262m|PACid:19273192  clementine0.9_013350m|PACid:19273712  clementine0.9_015439m|PACid:19273718  clementine0.9_029466m|PACid:19276201  clementine0.9_012449m|PACid:19280648  clementine0.9_034047m|PACid:19281654  clementine0.9_002294m|PACid:19282243  clementine0.9_003594m|PACid:19285627  clementine0.9_002251m|PACid:19286208  cclementine0.9_012397m|PACid:19285950 | AT1G52150.1  AT4G17800.1  AT1G67340.1  AT1G48210.1  AT3G14460.1  AT3G11460.1  AT4G32880.1  AT5G25830.1  AT5G19930.1  AT2G02040.1  AT2G43750.1  AT1G06950.1  AT5G60690.1  AT1G21270.1  AT2G34710.1  AT1G31050.1 | Homeobox-leucine zipper family protein / lipid-binding START domain-containing protein  Predicted AT-hook DNA-binding family protein  HCP-like superfamily protein with MYND-type zinc finger  Protein kinase superfamily protein  LRR and NB-ARC domains-containing disease resistance protein  Pentatricopeptide repeat (PPR) superfamily protein  Homeobox gene 8  GATA transcription factor 12  Protein of unknown function DUF92, transmembrane  Peptide transporter 2  O-acetylserine (thiol) lyase B  Translocon at the inner envelope membrane of chloroplasts 110  Homeobox-leucine zipper family protein / lipid-binding START domain-containing protein  Wall-associated kinase 2  Homeobox-leucine zipper family protein / lipid-binding START domain-containing protein  Basic helix-loop-helix (bHLH) DNA-binding superfamily protein |
| miR1847 | clementine0.9_003600m|PACid:19254954  clementine0.9_034910m|PACid:19255824  clementine0.9_005938m|PACid:19262762 | AT5G18500.1  AT4G03500.1  AT1G69730.1 | Protein kinase superfamily protein  Ankyrin repeat family protein  Wall-associated kinase family protein |
| miR1850 | clementine0.9_014925m|PACid:19259019  clementine0.9_013721m|PACid:19251983  clementine0.9_007731m|PACid:19275225  clementine0.9_003652m|PACid:19286433 | AT1G69010.1  AT5G10770.1  AT5G24120.1  AT5G13480.1 | BES1-interacting Myc-like protein 2  Eukaryotic aspartyl protease family protein  Sigma factor E  Transducin/WD40 repeat-like superfamily protein |
| miR1857 | clementine0.9_033548m|PACid:19252561  clementine0.9_004836m|PACid:19252207  clementine0.9_003671m|PACid:19252922  clementine0.9_034336m|PACid:19253105  clementine0.9_001965m|PACid:19255011  clementine0.9_025617m|PACid:19255484  clementine0.9_003873m|PACid:19258115  clementine0.9_004054m|PACid:19262333  clementine0.9_000575m|PACid:19263695  clementine0.9_002329m|PACid:19264813  clementine0.9_021072m|PACid:19263624  clementine0.9_032501m|PACid:19263659  clementine0.9_005361m|PACid:19265639  clementine0.9_007454m|PACid:19266249  clementine0.9_001579m|PACid:19266719  clementine0.9_004063m|PACid:19270453  clementine0.9_003484m|PACid:19270980  clementine0.9_030694m|PACid:19273135  clementine0.9_012450m|PACid:19274708  clementine0.9_013002m|PACid:19276139  clementine0.9_013338m|PACid:19278338  clementine0.9_034607m|PACid:19280062  clementine0.9_016638m|PACid:19282561  clementine0.9_019735m|PACid:19283512  clementine0.9_011470m|PACid:19283859  clementine0.9_025557m|PACid:19283768  clementine0.9_000523m|PACid:19286072  clementine0.9_005350m|PACid:19286013 | AT5G56730.1  AT4G03260.2  AT2G01210.1  AT1G16570.1  AT1G17260.1  AT2G05310.1  AT1G73920.1  AT5G55600.3  AT2G45540.1  AT3G02130.1  AT2G29420.1  AT5G49830.1  AT1G55490.2  AT4G31940.1  AT5G62670.1  AT3G12780.1  AT1G62310.1  AT1G77410.1  AT1G54150.1  AT2G30910.1  AT3G20650.1  AT1G49760.1  AT1G07310.1  AT5G13180.1  AT2G40810.2  AT5G05960.1  AT3G54460.1  AT3G11920.1 | Insulinase (Peptidase family M16) protein  Outer arm dynein light chain 1 protein  Leucine-rich repeat protein kinase family protein  UDP-Glycosyltransferase superfamily protein  Autoinhibited H(+)-ATPase isoform 10  Protein kinase superfamily protein  Alpha/beta-Hydrolases superfamily protein  Agenet domain-containing protein / bromo-adjacent homology (BAH) domain-containing protein  WD-40 repeat family protein / beige-related  Receptor-like protein kinase 2  Glutathione S-transferase tau 7  Exocyst complex component 84B  Chaperonin 60 beta  Cytochrome P450, family 82, subfamily C, polypeptide 4  H(+)-ATPase 11  Phosphoglycerate kinase 1  Transcription factor jumonji (jmjC) domain-containing protein  Beta-galactosidase 16  E3 Ubiquitin ligase family protein  Actin-related protein C1A  mRNA capping enzyme family protein  poly(A) binding protein 8  Calcium-dependent lipid-binding (CaLB domain) family protein  NAC domain containing protein 83  Homolog of yeast autophagy 18C  Bifunctional inhibitor/lipid-transfer protein/seed storage 2S albumin superfamily protein  SNF2 domain-containing protein / helicase domain-containing protein / F-box family protein  Glutaredoxin-related |
| miR1886 | clementine0.9_001067m|PACid:19251344  clementine0.9_003240m|PACid:19251546  clementine0.9_003403m|PACid:19251385  clementine0.9_005176m|PACid:19252976  clementine0.9_025304m|PACid:19258598  clementine0.9_000193m|PACid:19258770  clementine0.9_002530m|PACid:19262401  clementine0.9_016437m|PACid:19263338  clementine0.9_021762m|PACid:19264475  clementine0.9_015012m|PACid:19265838  clementine0.9_020383m|PACid:19270575  clementine0.9_013232m|PACid:19270822  clementine0.9_005811m|PACid:19274712  clementine0.9_003438m|PACid:19275006  clementine0.9_009213m|PACid:19274982  clementine0.9_018548m|PACid:19275907  clementine0.9_000769m|PACid:19277608  clementine0.9_024515m|PACid:19283018  clementine0.9_001615m|PACid:19286213  clementine0.9_006152m|PACid:19286541 | AT2G22610.2  AT4G38050.1  AT3G49740.1  AT5G48380.1  AT3G28917.1  AT2G13680.1  AT4G30020.1  AT4G02730.1  AT4G00850.1  AT3G20810.1  AT3G17880.1  AT4G18340.1  AT3G13810.1  AT2G41900.1  AT1G56190.1  AT1G56170.1  AT5G44510.1  AT4G27150.1  AT3G06880.2  AT3G26810.1 | Di-glucose binding protein with Kinesin motor domain  Xanthine/uracil permease family protein  Tetratricopeptide repeat (TPR)-like superfamily protein  BAK1-interacting receptor-like kinase 1  Mini zinc finger 2  Callose synthase 5  PA-domain containing subtilase family protein  Transducin/WD40 repeat-like superfamily protein  GRF1-interacting factor 3  2-Oxoglutarate (2OG) and Fe(II)-dependent oxygenase superfamily protein  Tetraticopeptide domain-containing thioredoxin  Glycosyl hydrolase superfamily protein  Indeterminate(ID)-domain 11  CCCH-type zinc finger protein with ARM repeat domain  Phosphoglycerate kinase family protein  Nuclear factor Y, subunit C2  Target of AVRB operation1  Seed storage albumin 2  Transducin/WD40 repeat-like superfamily protein  Auxin signaling F-box 2 |
| miR1917 | clementine0.9_007036m|PACid:19258061  clementine0.9_008603m|PACid:19251411  clementine0.9_025517m|PACid:19257894  clementine0.9_026226m|PACid:19258446  clementine0.9_008898m|PACid:19259029  clementine0.9_014091m|PACid:19262256  clementine0.9_014735m|PACid:19261937  clementine0.9_003674m|PACid:19263580  clementine0.9_002869m|PACid:19271391  clementine0.9_009726m|PACid:19272189  clementine0.9_029477m|PACid:19274924  clementine0.9_014573m|PACid:19276975  clementine0.9_003953m|PACid:19280389  clementine0.9_031364m|PACid:19285347 | AT1G18580.1  AT5G67610.1  AT1G63245.1  AT5G40080.1  AT1G70580.1  AT4G33110.2  AT5G25220.1  AT2G45340.1  AT1G03370.1  AT3G02100.1  AT3G13790.2  AT4G10960.1  AT2G32850.2  AT1G18000.1 | Galacturonosyltransferase 11  Uncharacterized conserved protein (DUF2215)  CLAVATA3/ESR-RELATED 14  Mitochondrial ribosomal protein L27  Alanine-2-oxoglutarate aminotransferase 2  S-adenosyl-L-methionine-dependent methyltransferases superfamily protein  KNOTTED1-like homeobox gene 3  Leucine-rich repeat protein kinase family protein  C2 calcium/lipid-binding and GRAM domain containing protein  UDP-Glycosyltransferase superfamily protein  Glycosyl hydrolases family 32 protein  UDP-D-glucose/UDP-D-galactose 4-epimerase 5  Protein kinase superfamily protein  Major facilitator superfamily protein |
| miR2099 | clementine0.9_000991m|PACid:19257686  clementine0.9_012483m|PACid:19268085  clementine0.9_028512m|PACid:19254985  clementine0.9_001844m|PACid:19268279  clementine0.9_003363m|PACid:19270059  clementine0.9_030086m|PACid:19273562  clementine0.9_003047m|PACid:19274927  clementine0.9_022520m|PACid:19279434  clementine0.9_007504m|PACid:19286378 | AT5G43630.1  AT3G51870.1  AT3G14470.1  AT3G14840.2  AT5G35560.1  AT5G44700.1  AT5G67090.1  AT2G32280.1  AT1G30370.1 | Zinc knuckle (CCHC-type) family protein  Mitochondrial substrate carrier family protein  NB-ARC domain-containing disease resistance protein  Leucine-rich repeat transmembrane protein kinase  DENN (AEX-3) domain-containing protein  Leucine-rich repeat transmembrane protein kinase  Subtilisin-like serine endopeptidase family protein  Protein of unknown function (DUF1218)  Alpha/beta-Hydrolases superfamily protein |
| miR2118 | clementine0.9_001514m|PACid:19254677  clementine0.9_000380m|PACid:19257198  clementine0.9_029582m|PACid:19258436  clementine0.9_033355m|PACid:19267329  clementine0.9_001085m|PACid:19277494  clementine0.9_034528m|PACid:19281322  clementine0.9_035456m|PACid:19286183 | AT3G14470.1  AT3G14460.1  AT3G02100.1  AT3G50950.1  AT5G17680.1  AT3G50950.2  AT1G30450.1 | NB-ARC domain-containing disease resistance protein  LRR and NB-ARC domains-containing disease resistance protein  UDP-Glycosyltransferase superfamily protein  HOPZ-ACTIVATED RESISTANCE 1  Disease resistance protein (TIR-NBS-LRR class), putative  HOPZ-ACTIVATED RESISTANCE 1  Cation-chloride co-transporter 1 |
| miR2594 | clementine0.9_003037m|PACid:19254064  clementine0.9_002330m|PACid:19252928  clementine0.9_009679m|PACid:19256893  clementine0.9_000305m|PACid:19258109  clementine0.9_012953m|PACid:19258530  clementine0.9_000261m|PACid:19259401  clementine0.9_025833m|PACid:19261575  clementine0.9_003033m|PACid:19262352  clementine0.9_003145m|PACid:19262353  clementine0.9_025413m|PACid:19263422  clementine0.9_030392m|PACid:19268841  clementine0.9_034437m|PACid:19269568  clementine0.9_012911m|PACid:19271541  clementine0.9_000581m|PACid:19273537  clementine0.9_000070m|PACid:19273066  clementine0.9_032428m|PACid:19273060  clementine0.9_002573m|PACid:19276605  clementine0.9_020736m|PACid:19276961  clementine0.9_022346m|PACid:19279330  clementine0.9_019080m|PACid:19279490  clementine0.9_011031m|PACid:19281030  clementine0.9_019617m|PACid:19281824  clementine0.9_002321m|PACid:19284694  clementine0.9_015109m|PACid:19285300  clementine0.9_020339m|PACid:19286361  clementine0.9_010246m|PACid:19287049 | AT5G67360.1  AT1G49540.1  AT5G24318.1  AT5G61140.2  AT5G15120.1  AT1G24300.1  AT2G44860.2  AT5G35180.2  AT5G49300.1  AT5G01990.1  AT4G04960.1  AT3G16530.1  AT5G46250.1  AT2G26610.1  AT1G77460.1  AT1G11040.1  AT4G24690.1  AT4G11150.1  AT4G22250.1  AT4G36130.1  AT1G15110.1  AT1G07890.7  AT3G51070.1  AT3G53970.1  AT2G34770.1  AT3G56900.1 | Subtilase family protein  Elongator protein 2  O-Glycosyl hydrolases family 17 protein  U5 small nuclear ribonucleoprotein helicase  Protein of unknown function (DUF1637)  GYF domain-containing protein  Ribosomal protein L24e family protein  Protein of unknown function (DUF1336)  GATA transcription factor 16  Auxin efflux carrier family protein  Concanavalin A-like lectin protein kinase family protein  Legume lectin family protein  RNA-binding protein  Transducin family protein / WD-40 repeat family protein  Armadillo/beta-catenin-like repeat ; C2 calcium/lipid-binding domain (CaLB) protein  HSP40/DnaJ peptide-binding protein  Ubiquitin-associated (UBA)/TS-N domain-containing protein / Octicosapeptide/Phox/Bemp1 (PB1) domain-containing protein  Vacuolar ATP synthase subunit E1  RING/U-box superfamily protein  Ribosomal protein L2 family  Phosphatidyl serine synthase family protein  Ascorbate peroxidase 1  S-adenosyl-L-methionine-dependent methyltransferases superfamily protein  Proteasome inhibitor-related  Fatty acid hydroxylase 1  Transducin/WD40 repeat-like superfamily protein |
| miR2612 | clementine0.9_018113m|PACid:19261697  clementine0.9_015655m|PACid:19251228  clementine0.9_015958m|PACid:19264376  clementine0.9_028255m|PACid:19264439  clementine0.9_035362m|PACid:19267448  clementine0.9_033538m|PACid:19269005  clementine0.9_028487m|PACid:19269303  clementine0.9_033379m|PACid:19269308  clementine0.9_030268m|PACid:19278220  clementine0.9_028193m|PACid:19282883  clementine0.9_008544m|PACid:19284891 | AT5G09230.7  AT5G67270.1  AT4G02810.1  AT3G24840.1  AT1G09950.1  AT4G34490.1  AT3G62020.1  AT1G02335.1  AT4G34880.1  AT2G44540.1  AT4G35880.1 | Sirtuin 2  End binding protein 1C  Protein of unknown function (DUF3049)  Sec14p-like phosphatidylinositol transfer family protein  RESPONSE TO ABA AND SALT 1  Cyclase associated protein 1  Germin-like protein 10  Germin-like protein subfamily 2 member 2 precursor  Amidase family protein  Glycosyl hydrolase 9B9  Eukaryotic aspartyl protease family protein |
| miR2622 | clementine0.9_021714m|PACid:19255696  clementine0.9_003084m|PACid:19253726  clementine0.9_003925m|PACid:19256802  clementine0.9_006077m|PACid:19258150  clementine0.9_009110m|PACid:19257803  clementine0.9_016181m|PACid:19259813  clementine0.9_007891m|PACid:19263282  clementine0.9_025280m|PACid:19263147  clementine0.9_012924m|PACid:19266627  clementine0.9_028773m|PACid:19275120  clementine0.9_029427m|PACid:19279406  clementine0.9_000890m|PACid:19286610 | AT4G35860.1  AT3G05090.2  AT2G30950.1  AT3G18440.1  AT2G45120.1  AT1G13635.2  AT5G57740.1  AT1G15740.1  AT5G06610.1  AT2G44940.1  AT5G06900.1  AT2G24030.1 | GTP-binding 2  Transducin/WD40 repeat-like superfamily protein  FtsH extracellular protease family  Aluminum-activated malate transporter 9  C2H2-like zinc finger protein  DNA glycosylase superfamily protein  XB3 ortholog 2 in Arabidopsis thaliana  Leucine-rich repeat family protein  Protein of unknown function (DUF620)  Integrase-type DNA-binding superfamily protein  Cytochrome P450, family 93, subfamily D, polypeptide 1  Zinc ion binding; nucleic acid binding |
| miR2646 | clementine0.9_029589m|PACid:19256464  clementine0.9_012343m|PACid:19253093  clementine0.9_008221m|PACid:19259725  clementine0.9_006667m|PACid:19261340  clementine0.9_008780m|PACid:19267741  clementine0.9_025900m|PACid:19267069  clementine0.9_033046m|PACid:19266564  clementine0.9_008044m|PACid:19270508  clementine0.9_001846m|PACid:19271815  clementine0.9_003415m|PACid:19272408  clementine0.9_004968m|PACid:19277340  clementine0.9_008162m|PACid:19276757  clementine0.9_009296m|PACid:19277414  clementine0.9_012551m|PACid:19277416  clementine0.9_017740m|PACid:19276813  clementine0.9_021823m|PACid:19277024  clementine0.9_007076m|PACid:19279192 | AT4G02600.1  AT1G77860.1  AT1G14280.1  AT5G04780.1  AT5G53840.1  AT3G51660.1  AT1G20670.1  AT5G03430.1  AT4G02400.1  AT5G54260.1  AT1G33170.1  AT1G17720.2  AT4G16845.1  AT5G51230.1  AT4G35785.3  AT1G64450.1  AT2G42920.1 | Seven transmembrane MLO family protein  Rhomboid-related intramembrane serine protease family protein  Phytochrome kinase substrate 2  Pentatricopeptide repeat (PPR) superfamily protein  F-box/RNI-like/FBD-like domains-containing protein  Tautomerase/MIF superfamily protein  DNA-binding bromodomain-containing protein  Phosphoadenosine phosphosulfate (PAPS) reductase family protein  U3 ribonucleoprotein (Utp) family protein  DNA repair and meiosis protein (Mre11)  S-adenosyl-L-methionine-dependent methyltransferases superfamily protein  Protein phosphatase 2A, regulatory subunit PR55  VEFS-Box of polycomb protein  VEFS-Box of polycomb protein  RNA-binding (RRM/RBD/RNP motifs) family protein  Glycine-rich protein family  Pentatricopeptide repeat (PPR-like) superfamily protein |
| miR2664 | clementine0.9_015697m|PACid:19278825 | AT1G06890.2 | Nodulin MtN21 /EamA-like transporter family protein |
| miR2921 | clementine0.9_033769m|PACid:19274292 | AT3G18200.1 | Nodulin MtN21 /EamA-like transporter family protein |
| miR2922 | clementine0.9_021069m|PACid:19255407  clementine0.9_019408m|PACid:19252403  clementine0.9_022120m|PACid:19254553  clementine0.9_008849m|PACid:19258435  clementine0.9_022003m|PACid:19261666  clementine0.9_019367m|PACid:19271708  clementine0.9_001414m|PACid:19273223  clementine0.9_001924m|PACid:19274652  clementine0.9_034915m|PACid:19275368  clementine0.9_002445m|PACid:19276764  clementine0.9_015034m|PACid:19284116  clementine0.9_001488m|PACid:19286369 | AT5G03080.1  AT4G28400.1  AT1G58280.1  AT2G32010.2  AT3G49010.3  AT2G15290.1  AT2G25800.1  AT5G56890.1  AT3G13770.1  AT4G32180.3  AT4G08790.1  AT4G04885.1 | Phosphatidic acid phosphatase (PAP2) family protein  Protein phosphatase 2C family protein  Phosphoglycerate mutase family protein  CVP2 like 1  Breast basic conserved 1  Translocon at inner membrane of chloroplasts 21  Protein of unknown function (DUF810)  Protein kinase superfamily protein  Pentatricopeptide repeat (PPR) superfamily protein  Pantothenate kinase 2  Nitrilase/cyanide hydratase and apolipoprotein N-acyltransferase family protein  PCF11P-similar protein 4 |
| miR2948 | clementine0.9_002037m|PACid:19273948  clementine0.9_010327m|PACid:19263057  clementine0.9_021416m|PACid:19266256 | AT2G26650.1  AT4G26750.1  AT1G49220.1 | K^+^ transporter 1  Hydroxyproline-rich glycoprotein family protein  RING/U-box superfamily protein |
| miR3440 | clementine0.9_000782m|PACid:19256272  clementine0.9_004798m|PACid:19254074  clementine0.9_006351m|PACid:19253721  clementine0.9_000336m|PACid:19259509  clementine0.9_005825m|PACid:19259336  clementine0.9_002752m|PACid:19263309  clementine0.9_007085m|PACid:19262962  clementine0.9_000097m|PACid:19265711  clementine0.9_005654m|PACid:19267199  clementine0.9_018415m|PACid:19270364  clementine0.9_033934m|PACid:19271135  clementine0.9_005058m|PACid:19271853  clementine0.9_000099m|PACid:19279014  clementine0.9_002333m|PACid:19279360  clementine0.9_006782m|PACid:19279322  clementine0.9_000190m|PACid:19282108  clementine0.9_013614m|PACid:19283484  clementine0.9_011927m|PACid:19285106  clementine0.9_007015m|PACid:19286202  clementine0.9_014908m|PACid:19286824 | AT5G14950.1  AT5G49980.1  AT2G18750.2  AT3G13080.1  AT1G67710.1  AT5G33280.1  AT2G26200.1  AT5G13000.1  AT3G53960.1  AT4G02610.1  AT1G09890.1  AT2G17250.1  AT1G06490.1  AT1G29370.1  AT1G06520.1  AT5G22760.1  AT3G51970.1  AT4G36680.1  AT1G30700.1  AT1G66520.1 | Golgi alpha-mannosidase II  Auxin F-box protein 5  Calmodulin-binding protein  Multidrug resistance-associated protein 3  Response regulator 11  Voltage-gated chloride channel family protein  S-adenosyl-L-methionine-dependent methyltransferases superfamily protein  Glucan synthase-like 12  Major facilitator superfamily protein  Aldolase-type TIM barrel family protein  Rhamnogalacturonate lyase family protein  CCAAT-binding factor  Glucan synthase-like 7  Kinase-related protein of unknown function (DUF1296)  Glycerol-3-phosphate acyltransferase 1  PHD finger family protein  Acyl-CoA sterol acyl transferase 1  Tetratricopeptide repeat (TPR)-like superfamily protein  FAD-binding Berberine family protein  Formyltransferase, putative |
| miR3443 | clementine0.9_005560m|PACid:19251361  clementine0.9_017919m|PACid:19259025  clementine0.9_013624m|PACid:19261898  clementine0.9_032408m|PACid:19261865  clementine0.9_034883m|PACid:19261859  clementine0.9_003710m|PACid:19264383  clementine0.9_027419m|PACid:19270559  clementine0.9_005595m|PACid:19270646  clementine0.9_025662m|PACid:19270614  clementine0.9_002054m|PACid:19272566  clementine0.9_016112m|PACid:19273758  clementine0.9_008154m|PACid:19275823  clementine0.9_011410m|PACid:19277140  clementine0.9_003134m|PACid:19279318  clementine0.9_000384m|PACid:19280461  clementine0.9_009899m|PACid:19282327 | AT5G67530.1  AT1G67330.1  AT5G12040.1  AT2G13600.1  AT2G19090.1  AT2G47160.1  AT3G17980.1  AT5G47800.1  AT1G30135.1  AT3G59420.1  AT5G25560.1  AT5G24060.2  AT1G74910.1  AT3G21620.1  AT1G64570.1  AT2G29090.1 | Plant U-box 49  Protein of unknown function (DUF579)  Nitrilase/cyanide hydratase and apolipoprotein N-acyltransferase family protein  Pentatricopeptide repeat (PPR) superfamily protein  Protein of unknown function (DUF630 and DUF632)  HCO3- transporter family  Calcium-dependent lipid-binding (CaLB domain) family protein  Phototropic-responsive NPH3 family protein  Jasmonate-zim-domain protein 8  crinkly4  CHY-type/CTCHY-type/RING-type Zinc finger protein  Pentatricopeptide repeat (PPR) superfamily protein  ADP-glucose pyrophosphorylase family protein  ERD (early-responsive to dehydration stress) family protein  Homeodomain-like superfamily protein  Cytochrome P450, family 707, subfamily A, polypeptide 2 |
| miR3454 | clementine0.9_000757m|PACid:19258828  clementine0.9_021137m|PACid:19263618  clementine0.9_002497m|PACid:19272356  clementine0.9_001794m|PACid:19272916  clementine0.9_007102m|PACid:19277446  clementine0.9_013888m|PACid:19280498  clementine0.9_003516m|PACid:19280893  clementine0.9_004361m|PACid:19282517  clementine0.9_004887m|PACid:19282518 | AT5G04930.1  AT4G01940.1  AT4G21380.1  AT1G22620.1  AT4G16820.1  AT3G07130.1  AT2G01690.1  AT1G07705.2  AT5G59710.1 | Aminophospholipid ATPase 1  NFU domain protein 1  Receptor kinase 3  Phosphoinositide phosphatase family protein  Alpha/beta-Hydrolases superfamily protein  Purple acid phosphatase 15  ARM repeat superfamily protein  NOT2 / NOT3 / NOT5 family  VIRE2 interacting protein 2 |
| miR3455 | clementine0.9_014940m|PACid:19251439  clementine0.9_009313m|PACid:19251586  clementine0.9_007074m|PACid:19251974  clementine0.9_014229m|PACid:19252321  clementine0.9_020821m|PACid:19252189  clementine0.9_010970m|PACid:19255219  clementine0.9_002010m|PACid:19255980  clementine0.9_003929m|PACid:19256039  clementine0.9_034224m|PACid:19256612  clementine0.9_009992m|PACid:19258722  clementine0.9_004688m|PACid:19259394  clementine0.9_025119m|PACid:19261070  clementine0.9_003050m|PACid:19262840  clementine0.9_010675m|PACid:19263245  clementine0.9_000791m|PACid:19264154  clementine0.9_006354m|PACid:19265027  clementine0.9_017603m|PACid:19263922  clementine0.9_030334m|PACid:19265687  clementine0.9_004177m|PACid:19268680  clementine0.9_005565m|PACid:19268612  clementine0.9_008519m|PACid:19269215  clementine0.9_016443m|PACid:19273895  clementine0.9_006944m|PACid:19272981  clementine0.9_020117m|PACid:19272814  clementine0.9_001526m|PACid:19274067  clementine0.9_032619m|PACid:19274321  clementine0.9_008784m|PACid:19274511  clementine0.9_004339m|PACid:19275777  clementine0.9_012874m|PACid:19275802  clementine0.9_006893m|PACid:19276185  clementine0.9_001583m|PACid:19280278  clementine0.9_005259m|PACid:19280397  clementine0.9_035478m|PACid:19281859  clementine0.9_008875m|PACid:19284395  clementine0.9_011898m|PACid:19283898  clementine0.9_000130m|PACid:19284981  clementine0.9_002953m|PACid:19285157 | AT4G33950.1  AT3G16520.3  AT2G24820.1  AT5G50960.1  AT4G27670.1  AT1G53750.1  AT4G08850.1  AT1G35710.1  AT3G14470.1  AT3G26040.1  AT2G03890.1  AT1G48160.1  AT2G07050.1  AT3G13050.1  AT1G02730.1  AT4G00550.1  AT1G16860.1  AT4G08850.2  AT3G30300.1  AT4G19170.1  AT5G65170.1  AT1G11020.1  AT4G09160.1  AT1G21090.1  AT1G53310.1  AT3G23250.1  AT4G37330.1  AT1G21270.1  AT4G11070.1  AT1G09970.2  AT2G34930.1  AT2G44430.1  AT1G59780.1  AT4G00830.1  AT2G39760.1  AT4G35800.1  AT3G54350.2 | Protein kinase superfamily protein  UDP-glucosyl transferase 88A1  Translocon at the inner envelope membrane of chloroplasts 55-II  Nucleotide binding protein 35  Heat shock protein 21  Regulatory particle triple-A 1A  Leucine-rich repeat receptor-like protein kinase family protein  Protein kinase family protein with leucine-rich repeat domain  NB-ARC domain-containing disease resistance protein  HXXXD-type acyl-transferase family protein  Phosphoinositide 4-kinase gamma 7  Signal recognition particle 19 kDa protein, putative / SRP19, putative  Cycloartenol synthase 1  Major facilitator superfamily protein  Cellulose synthase-like D5  Digalactosyl diacylglycerol deficient 2  Ubiquitin-specific protease family C19-related protein  Leucine-rich repeat receptor-like protein kinase family protein  O-fucosyltransferase family protein  Nine-cis-epoxycarotenoid dioxygenase 4  VQ motif-containing protein  RING/FYVE/PHD zinc finger superfamily protein  SEC14 cytosolic factor family protein / phosphoglyceride transfer family protein  Cupredoxin superfamily protein  Phosphoenolpyruvate carboxylase 1  Myb domain protein 15  Cytochrome P450, family 81, subfamily D, polypeptide 4  Wall-associated kinase 2  WRKY family transcription factor  Leucine-rich receptor-like protein kinase family protein  Disease resistance family protein / LRR family protein  DNA-binding bromodomain-containing protein  NB-ARC domain-containing disease resistance protein  RNA-binding (RRM/RBD/RNP motifs) family protein  BTB/POZ/MATH-domains containing protein  RNA polymerase II large subunit  Forkhead-associated (FHA) domain-containing protein |
| miR3456 | clementine0.9_000254m|PACid:19263791  clementine0.9_034860m|PACid:19255200  clementine0.9_030488m|PACid:19258473 | AT4G00060.1  AT1G12210.1  AT3G28880.1 | Nucleotidyltransferase family protein  RPS5-like 1  Ankyrin repeat family protein |
| miR3462 | clementine0.9_008885m|PACid:19251571  clementine0.9_003477m|PACid:19255314  clementine0.9_024859m|PACid:19257680  clementine0.9_000976m|PACid:19261150  clementine0.9_020674m|PACid:19260491  clementine0.9_020867m|PACid:19260442  clementine0.9_016266m|PACid:19264202  clementine0.9_036085m|PACid:19267325  clementine0.9_018087m|PACid:19268719  clementine0.9_018341m|PACid:19270827  clementine0.9_000255m|PACid:19274184  clementine0.9_007478m|PACid:19274855  clementine0.9_006539m|PACid:19275810  clementine0.9_012624m|PACid:19279151  clementine0.9_014696m|PACid:19279165  clementine0.9_016033m|PACid:19280919  clementine0.9_002013m|PACid:19281689  clementine0.9_014895m|PACid:19283678  clementine0.9_002629m|PACid:19283953  clementine0.9_015416m|PACid:19284416  clementine0.9_024102m|PACid:19285098  clementine0.9_005399m|PACid:19286152 | AT5G10550.1  AT2G13440.1  AT2G31200.1  AT3G16785.1  AT5G02140.1  AT3G53570.4  AT2G47490.1  AT5G06740.1  AT5G58890.1  AT1G29280.1  AT4G26090.1  AT4G02630.1  AT4G31180.2  AT3G25210.1  AT1G30540.1  AT1G71080.1  AT2G45910.1  AT1G60690.1  AT5G05680.1  AT2G40620.1  AT1G75440.1  AT4G21120.1 | Global transcription factor group E2  Glucose-inhibited division family A protein  Actin depolymerizing factor 6  Phospholipase D P1  Pathogenesis-related thaumatin superfamily protein  FUS3-complementing gene 1  NAD+ transporter 1  Concanavalin A-like lectin protein kinase family protein  AGAMOUS-like 82  WRKY DNA-binding protein 65  NB-ARC domain-containing disease resistance protein  Protein kinase superfamily protein  Class II aminoacyl-tRNA and biotin synthetases superfamily protein  Tetratricopeptide repeat (TPR)-like superfamily protein  Actin-like ATPase superfamily protein  RNA polymerase II transcription elongation factor  U-box domain-containing protein kinase family protein  NAD(P)-linked oxidoreductase superfamily protein  nuclear pore complex protein-related  Basic-leucine zipper (bZIP) transcription factor family protein  Ubiquitin-conjugating enzyme 16  Amino acid transporter 1 |
| miR3465 | clementine0.9_001178m|PACid:19280124 | AT3G57330.1 | Autoinhibited Ca^2+^-ATPase 11 |
| miR3513 | clementine0.9_034509m|PACid:19266470  clementine0.9_004896m|PACid:19282895 | AT4G13810.1  AT1G02205.2 | Receptor like protein 47  Fatty acid hydroxylase superfamily |
| miR3932 | clementine0.9_005580m|PACid:19251585  clementine0.9_006668m|PACid:19251541  clementine0.9_002624m|PACid:19251323  clementine0.9_011190m|PACid:19251542  clementine0.9_021452m|PACid:19251291  clementine0.9_033848m|PACid:19251600  clementine0.9_033510m|PACid:19252534  clementine0.9_009997m|PACid:19253878  clementine0.9_015732m|PACid:19253591  clementine0.9_019993m|PACid:19253979  clementine0.9_000968m|PACid:19255006  clementine0.9_003983m|PACid:19255745  clementine0.9_011852m|PACid:19256201  clementine0.9_012053m|PACid:19255783  clementine0.9_000989m|PACid:19257072  clementine0.9_029468m|PACid:19256992  clementine0.9_018770m|PACid:19258502  clementine0.9_000877m|PACid:19258745  clementine0.9_004695m|PACid:19259579  clementine0.9_006566m|PACid:19258957  clementine0.9_023683m|PACid:19260175  clementine0.9_007938m|PACid:19261018  clementine0.9_010624m|PACid:19260720  clementine0.9_001288m|PACid:19261493  clementine0.9_002472m|PACid:19262899  clementine0.9_009333m|PACid:19262946  clementine0.9_024184m|PACid:19262557  clementine0.9_029903m|PACid:19262955  clementine0.9_002389m|PACid:19264755  clementine0.9_002839m|PACid:19263980  clementine0.9_002836m|PACid:19263981  clementine0.9_003366m|PACid:19264216  clementine0.9_008965m|PACid:19263708  clementine0.9_014342m|PACid:19263804  clementine0.9_034440m|PACid:19263597  clementine0.9_003706m|PACid:19265776  clementine0.9_022005m|PACid:19266044  clementine0.9_032197m|PACid:19266269  clementine0.9_012010m|PACid:19267421  clementine0.9_018471m|PACid:19268020  clementine0.9_034436m|PACid:19267953  clementine0.9_036009m|PACid:19266605  clementine0.9_004564m|PACid:19268387  clementine0.9_026484m|PACid:19268963  clementine0.9_030873m|PACid:19269369  clementine0.9_014152m|PACid:19270117 | AT1G72160.1  AT2G22730.1  AT5G65500.1  AT5G64500.1  AT2G22540.1  AT3G49690.1  AT3G14470.1  AT4G24550.2  AT1G07840.2  AT1G44830.1  AT3G47570.1  AT5G04590.1  AT2G32560.1  AT2G13360.2  AT1G80490.1  AT1G52570.1  AT3G29010.1  AT5G64070.1  AT1G70370.1  AT1G23400.1  AT1G69180.1  AT5G02410.1  AT5G02820.1  AT3G47570.1  AT4G29950.1  AT4G30610.1  AT1G13950.1  AT3G26320.1  AT3G62240.1  AT3G03770.2  AT3G03770.1  AT4G28600.1  AT4G00830.1  AT3G61740.1  AT1G04150.1  AT3G13460.1  AT3G22320.1  AT5G45480.1  AT4G11970.2  AT2G47140.1  AT5G03730.2  AT5G01550.1  AT1G17230.1  AT1G75250.2  AT3G46710.1  AT2G39970.1 | Sec14p-like phosphatidylinositol transfer family protein  Major facilitator superfamily protein  U-box domain-containing protein kinase family protein  Major facilitator superfamily protein  K-box region and MADS-box transcription factor family protein  Myb domain protein 84  NB-ARC domain-containing disease resistance protein  Clathrin adaptor complexes medium subunit family protein  Sas10/Utp3/C1D family  Integrase-type DNA-binding superfamily protein  Leucine-rich repeat protein kinase family protein  Sulfite reductase  F-box family protein  Alanine:glyoxylate aminotransferase  TOPLESS-related 1  Phospholipase D alpha 2  Biotin/lipoate A/B protein ligase family  Phosphatidylinositol 4-OH kinase beta1  Polygalacturonase 2  RNA-binding CRS1 / YhbY (CRM) domain-containing protein  Plant-specific transcription factor YABBY family protein  DIE2/ALG10 family  Spo11/DNA topoisomerase VI, subunit A protein  Leucine-rich repeat protein kinase family protein  Ypt/Rab-GAP domain of gyp1p superfamily protein  Alpha/beta-Hydrolases superfamily protein  Eukaryotic elongation factor 5A-1  Cytochrome P450, family 71, subfamily B, polypeptide 36  RING/U-box superfamily protein  Leucine-rich repeat protein kinase family protein  Leucine-rich repeat protein kinase family protein  No pollen germination related 2  RNA-binding (RRM/RBD/RNP motifs) family protein  SET domain protein 14  C2 calcium/lipid-binding plant phosphoribosyltransferase family protein  Evolutionarily conserved C-terminal region 2  Eukaryotic rpb5 RNA polymerase subunit family protein  Protein of unknown function (DUF594)  YTH family protein  NAD(P)-binding Rossmann-fold superfamily protein  Protein kinase superfamily protein  Lectin receptor kinase a4.1  Leucine-rich receptor-like protein kinase family protein  RAD-like 6  NB-ARC domain-containing disease resistance protein  Mitochondrial substrate carrier family protein |
| miR394 | clementine0.9_031415m|PACid:19276919  clementine0.9_008838m|PACid:19253029  clementine0.9_010509m|PACid:19278280  clementine0.9_012184m|PACid:19281346  clementine0.9_000058m|PACid:19281445 | AT2G30750.1  AT3G28670.1  AT4G38760.1  AT5G53750.1  AT1G20960.2 | Cytochrome P450, family 71, subfamily A, polypeptide 12  Oxidoreductase, zinc-binding dehydrogenase family protein  Protein of unknown function (DUF3414)  CBS domain-containing protein  U5 small nuclear ribonucleoprotein helicase, putative |
| miR418 | clementine0.9_008037m|PACid:19251659  clementine0.9_002640m|PACid:19266698  clementine0.9_000100m|PACid:19251836  clementine0.9_008146m|PACid:19251973  clementine0.9_023302m|PACid:19252000  clementine0.9_023921m|PACid:19251936  clementine0.9_021323m|PACid:19252562  clementine0.9_003255m|PACid:19253194  clementine0.9_007133m|PACid:19254615  clementine0.9_031876m|PACid:19254148  clementine0.9_028679m|PACid:19254943  clementine0.9_014058m|PACid:19256547  clementine0.9_021464m|PACid:19258474  clementine0.9_008959m|PACid:19258968  clementine0.9_009516m|PACid:19260063  clementine0.9_002178m|PACid:19260255  clementine0.9_004098m|PACid:19263022  clementine0.9_000049m|PACid:19265068  clementine0.9_004113m|PACid:19263798  clementine0.9_005596m|PACid:19264662  clementine0.9_016218m|PACid:19264161  clementine0.9_021394m|PACid:19266315  clementine0.9_027973m|PACid:19266263  clementine0.9_000709m|PACid:19267838  clementine0.9_001781m|PACid:19268083  clementine0.9_001929m|PACid:19266696  clementine0.9_030068m|PACid:19269676  clementine0.9_034791m|PACid:19269829  clementine0.9_001298m|PACid:19271480  clementine0.9_020130m|PACid:19271041  clementine0.9_012142m|PACid:19271701  clementine0.9_012320m|PACid:19271960  clementine0.9_009117m|PACid:19273741  clementine0.9_001160m|PACid:19273148  clementine0.9_005276m|PACid:19274763  clementine0.9_030287m|PACid:19278113  clementine0.9_018265m|PACid:19279843  clementine0.9_004553m|PACid:19279986  clementine0.9_017882m|PACid:19280118  clementine0.9_017328m|PACid:19280528  clementine0.9_011940m|PACid:19281153  clementine0.9_017146m|PACid:19283462  clementine0.9_034128m|PACid:19283601  clementine0.9_018111m|PACid:19284188  clementine0.9_006211m|PACid:19285663  clementine0.9_001705m|PACid:19285877  clementine0.9_004999m|PACid:19287024 | AT4G09570.1  AT3G11560.4  AT4G31160.1  AT4G31140.1  AT1G28280.1  AT5G10620.1  AT4G29160.1  AT1G08070.1  AT1G47240.1  AT3G52460.1  AT4G20800.1  AT2G44060.2  AT2G04690.1  AT5G43270.1  AT5G40270.1  AT3G04690.1  AT2G25180.1  AT2G46020.2  AT3G24660.1  AT4G00740.1  AT4G01370.1  AT5G64813.1  AT5G22355.1  AT4G27190.1  AT3G11540.1  AT3G11560.4  AT2G03760.1  AT3G26040.1  AT4G18240.1  AT5G44210.1  AT4G35310.1  AT4G34950.1  AT4G32770.1  AT4G08350.1  AT1G72970.1  AT5G58003.1  AT1G58210.1  AT5G07360.1  AT2G41530.1  AT3G58600.1  AT1G08750.1  AT5G13730.1  AT3G52080.1  AT2G40200.1  AT3G07740.1  AT4G20910.2  AT1G48480.1 | Calcium-dependent protein kinase 4  LETM1-like protein  DDB1-CUL4 associated factor 1  O-Glycosyl hydrolases family 17 protein  VQ motif-containing protein  methyltransferases  SNF7 family protein  Tetratricopeptide repeat (TPR)-like superfamily protein  NRAMP metal ion transporter 2  Hydroxyproline-rich glycoprotein family protein  FAD-binding Berberine family protein  Late embryogenesis abundant protein, group 2  Pyridoxamine 5\'-phosphate oxidase family protein  Squamosa promoter binding protein-like 2  HD domain-containing metal-dependent phosphohydrolase family protein  Malectin/receptor-like protein kinase family protein  Response regulator 12  Transcription regulatory protein SNF2, putative  Transmembrane kinase-like 1  S-adenosyl-L-methionine-dependent methyltransferases superfamily protein  MAP kinase 4  Ras-related small GTP-binding family protein  Cysteine/Histidine-rich C1 domain family protein  NB-ARC domain-containing disease resistance protein  Tetratricopeptide repeat (TPR)-like superfamily protein  LETM1-like protein  Sulphotransferase 12  HXXXD-type acyl-transferase family protein  Starch synthase 4  Erf domain protein 9  Calmodulin-domain protein kinase 5  Major facilitator superfamily protein  Tocopherol cyclase, chloroplast / vitamin E deficient 1 (VTE1) / sucrose export defective 1 (SXD1)  Global transcription factor group A2  Glucose-methanol-choline (GMC) oxidoreductase family protein  C-terminal domain phosphatase-like 4  Kinase interacting family protein  Amidase family protein  S-formylglutathione hydrolase  Adaptin ear-binding coat-associated protein 1 NECAP-1  Peptidase C13 family  Sigma factor 4  Cation/hydrogen exchanger 28  Basic helix-loop-helix (bHLH) DNA-binding superfamily protein  Homolog of yeast ADA2 2A  Double-stranded RNA binding protein-related / DsRBD protein-related  Receptor-like kinase 1 |
| miR4342 | clementine0.9_020265m|PACid:19271236 | AT5G47760.1 | 2-Phosphoglycolate phosphatase 2 |
| miR435 | clementine0.9_000965m|PACid:19254664  clementine0.9_016938m|PACid:19251518  clementine0.9_017500m|PACid:19251975  clementine0.9_022899m|PACid:19254902  clementine0.9_029569m|PACid:19256839  clementine0.9_000350m|PACid:19258637  clementine0.9_006453m|PACid:19258738  clementine0.9_004111m|PACid:19260760  clementine0.9_004884m|PACid:19261513  clementine0.9_026135m|PACid:19265360  clementine0.9_005344m|PACid:19266287  clementine0.9_008688m|PACid:19266982  clementine0.9_026557m|PACid:19268749  clementine0.9_005431m|PACid:19268938  clementine0.9_017616m|PACid:19273174  clementine0.9_034890m|PACid:19275598  clementine0.9_003990m|PACid:19276610  clementine0.9_008260m|PACid:19279134  clementine0.9_023362m|PACid:19283736  clementine0.9_003809m|PACid:19286529  clementine0.9_005911m|PACid:19286531 | AT3G03050.1  AT1G10380.1  AT5G10750.1  AT5G11900.1  AT3G02060.1  AT1G13980.2  AT2G13690.1  AT1G07630.1  AT2G28600.1  AT4G00755.1  AT4G36710.1  AT2G38120.1  AT5G16060.1  AT5G53370.1  AT5G11910.1  AT3G26040.1  AT5G41410.1  AT5G45110.1  AT2G37430.1  AT5G41120.1  AT3G02030.1 | Cellulose synthase-like D3  Putative membrane lipoprotein  Protein of unknown function (DUF1336)  Translation initiation factor SUI1 family protein  DEAD/DEAH box helicase, putative  Sec7 domain-containing protein  PRLI-interacting factor, putative  Pol-like 5  P-loop containing nucleoside triphosphate hydrolases superfamily protein  F-box family protein  GRAS family transcription factor  Transmembrane amino acid transporter family protein  Cytochrome c oxidase biogenesis protein Cmc1-like  Pectin methylesterase PCR fragment F  Alpha/beta-Hydrolases superfamily protein  HXXXD-type acyl-transferase family protein  POX (plant homeobox) family protein  NPR1-like protein 3  C2H2 and C2HC zinc fingers superfamily protein  Esterase/lipase/thioesterase family protein  Transferases, transferring acyl groups other than amino-acyl groups; acyltransferases |
| miR437 | clementine0.9_035054m|PACid:19260266  clementine0.9_017297m|PACid:19265407  clementine0.9_015953m|PACid:19267017  clementine0.9_000276m|PACid:19274182  clementine0.9_031711m|PACid:19286360 | AT1G48090.1  AT2G02570.4  AT2G38550.1  AT4G27190.1  AT3G54450.1 | Calcium-dependent lipid-binding family protein  Nucleic acid binding;RNA binding  Transmembrane proteins 14C  NB-ARC domain-containing disease resistance protein  Major facilitator superfamily protein |
| miR4406 | clementine0.9_018322m|PACid:19278066 | AT5G49290.1 | Receptor like protein 56 |
| miR4413 | clementine0.9_002923m|PACid:19261991  clementine0.9_017797m|PACid:19266350  clementine0.9_006027m|PACid:19271812  clementine0.9_028989m|PACid:19282160  clementine0.9_011458m|PACid:19283343 | AT5G47430.3  AT2G18465.1  AT2G15530.2  AT1G11330.1  AT3G08505.1 | DWNN domain, a CCHC-type zinc finger  Chaperone DnaJ-domain superfamily protein  RING/U-box superfamily protein  S-locus lectin protein kinase family protein  zinc finger (CCCH-type/C3HC4-type RING finger) family protein |
| miR472 | clementine0.9_000883m|PACid:19252485  clementine0.9_032351m|PACid:19253823  clementine0.9_002232m|PACid:19255072  clementine0.9_035618m|PACid:19255262  clementine0.9_030591m|PACid:19255601  clementine0.9_001280m|PACid:19266747 | AT4G12010.1  AT4G27190.1  AT1G61180.1  AT5G63020.1  AT4G27190.1  AT1G12210.1 | Disease resistance protein (TIR-NBS-LRR class) family  NB-ARC domain-containing disease resistance protein  LRR and NB-ARC domains-containing disease resistance protein  Disease resistance protein (CC-NBS-LRR class) family  NB-ARC domain-containing disease resistance protein  RPS5-like 1 |
| miR5014 | clementine0.9_011749m|PACid:19251829  clementine0.9_002669m|PACid:19255183  clementine0.9_006465m|PACid:19251938  clementine0.9_005008m|PACid:19256970  clementine0.9_006667m|PACid:19261340  clementine0.9_034756m|PACid:19262482  clementine0.9_009738m|PACid:19263973  clementine0.9_009426m|PACid:19268783  clementine0.9_003562m|PACid:19269530  clementine0.9_007324m|PACid:19278998  clementine0.9_008619m|PACid:19280629  clementine0.9_013329m|PACid:19281078  clementine0.9_001757m|PACid:19281421  clementine0.9_013695m|PACid:19282956  clementine0.9_008663m|PACid:19285121  clementine0.9_013897m|PACid:19285034  clementine0.9_010101m|PACid:19286873 | AT4G31770.1  AT3G16290.1  AT5G10650.2  AT4G32250.1  AT5G04780.1  AT3G59500.1  AT2G46670.1  AT5G38510.1  AT3G15930.1  AT1G08680.4  AT2G43850.1  AT1G24610.1  AT5G54200.1  AT2G44760.1  AT1G75220.1  AT4G36810.1  AT5G05140.1 | Debranching enzyme 1  AAA-type ATPase family protein  RING/U-box superfamily protein  Protein kinase superfamily protein  Pentatricopeptide repeat (PPR) superfamily protein  Integral membrane HRF1 family protein  CCT motif family protein  Rhomboid-related intramembrane serine protease family protein  Pentatricopeptide repeat (PPR) superfamily protein  ARF GAP-like zinc finger-containing protein ZIGA4  Integrin-linked protein kinase family  Rubisco methyltransferase family protein  Transducin/WD40 repeat-like superfamily protein  Domain of unknown function (DUF3598)  Major facilitator superfamily protein  Geranylgeranyl pyrophosphate synthase 1  Transcription elongation factor (TFIIS) family protein |
| miR5023 | clementine0.9_013684m|PACid:19283327  clementine0.9_002391m|PACid:19286425 | AT3G21640.1  AT5G45160.1 | FKBP-type peptidyl-prolyl cis-trans isomerase family protein  Root hair defective 3 GTP-binding protein (RHD3) |
| miR5028 | clementine0.9_013687m|PACid:19276737 | AT5G17990.1 | Tryptophan biosynthesis 1 |
| miR5029 | clementine0.9_000349m|PACid:19255227  clementine0.9_020910m|PACid:19258467  clementine0.9_030240m|PACid:19258611  clementine0.9_011450m|PACid:19259843  clementine0.9_022931m|PACid:19260550  clementine0.9_000777m|PACid:19267694  clementine0.9_004370m|PACid:19267802  clementine0.9_014266m|PACid:19277080  clementine0.9_002815m|PACid:19278892  clementine0.9_013264m|PACid:19279830  clementine0.9_015257m|PACid:19280579  clementine0.9_028130m|PACid:19282077  clementine0.9_009607m|PACid:19283073  clementine0.9_009940m|PACid:19284499  clementine0.9_012828m|PACid:19286551 | AT1G15520.1  AT3G01170.1  AT1G80170.1  AT1G67850.2  AT4G15630.1  AT1G45616.1  AT5G01720.1  AT1G64150.1  AT5G49890.1  AT1G10830.1  AT3G15030.1  AT5G22860.1  AT1G56400.1  AT3G19184.1  AT1G28050.1 | pleiotropic drug resistance 12  Ribosomal protein L34e superfamily protein  Pectin lyase-like superfamily protein  Protein of unknown function (DUF707)  Uncharacterised protein family (UPF0497)  Receptor like protein 6  RNI-like superfamily protein  Uncharacterized protein family (UPF0016)  Chloride channel C  15-cis-zeta-carotene isomerase  TCP family transcription factor 4  Serine carboxypeptidase S28 family protein  F-box family protein  AP2/B3-like transcriptional factor family protein  B-box type zinc finger protein with CCT domain |
| miR5056 | clementine0.9_007143m|PACid:19251539  clementine0.9_031819m|PACid:19251941  clementine0.9_013003m|PACid:19252055  clementine0.9_007313m|PACid:19253402  clementine0.9_029178m|PACid:19254509  clementine0.9_031988m|PACid:19253446  clementine0.9_012836m|PACid:19254819  clementine0.9_002247m|PACid:19255046  clementine0.9_002908m|PACid:19255094  clementine0.9_007767m|PACid:19255272  clementine0.9_013475m|PACid:19256291  clementine0.9_009380m|PACid:19256336  clementine0.9_009660m|PACid:19256690  clementine0.9_013604m|PACid:19258145  clementine0.9_010701m|PACid:19258629  clementine0.9_002092m|PACid:19260132  clementine0.9_007988m|PACid:19260688  clementine0.9_009837m|PACid:19261062  clementine0.9_033410m|PACid:19261045  clementine0.9_000005m|PACid:19262607  clementine0.9_001048m|PACid:19262248  clementine0.9_008276m|PACid:19262486  clementine0.9_032583m|PACid:19261869  clementine0.9_000075m|PACid:19263927  clementine0.9_000291m|PACid:19264534  clementine0.9_001378m|PACid:19264360  clementine0.9_001991m|PACid:19265043  clementine0.9_014730m|PACid:19265137  clementine0.9_023806m|PACid:19264739  clementine0.9_032172m|PACid:19265307  clementine0.9_012225m|PACid:19265849  clementine0.9_000059m|PACid:19266852  clementine0.9_004185m|PACid:19267870  clementine0.9_006263m|PACid:19266981  clementine0.9_011812m|PACid:19267253  clementine0.9_014659m|PACid:19267419  clementine0.9_000442m|PACid:19270908  clementine0.9_032833m|PACid:19271455  clementine0.9_008922m|PACid:19272147  clementine0.9_012365m|PACid:19273532  clementine0.9_019287m|PACid:19273004  clementine0.9_021730m|PACid:19273056  clementine0.9_003390m|PACid:19274578  clementine0.9_000745m|PACid:19275021  clementine0.9_008239m|PACid:19274975  clementine0.9_009485m|PACid:19275505  clementine0.9_028834m|PACid:19276331  clementine0.9_000231m|PACid:19277260  clementine0.9_001659m|PACid:19277153  clementine0.9_017407m|PACid:19276406  clementine0.9_001085m|PACid:19277494  clementine0.9_032090m|PACid:19279425  clementine0.9_000740m|PACid:19281821  clementine0.9_002013m|PACid:19281689  clementine0.9_012109m|PACid:19282250  clementine0.9_012518m|PACid:19281939  clementine0.9_009637m|PACid:19283570  clementine0.9_009682m|PACid:19284104  clementine0.9_034322m|PACid:19284384  clementine0.9_016377m|PACid:19285087  clementine0.9_005086m|PACid:19285564  clementine0.9_006214m|PACid:19285565 | AT2G30390.1  AT4G31480.1  AT4G28390.1  AT1G08960.1  AT2G33590.1  AT4G08290.2  AT1G43770.2  AT1G79930.1  AT3G14240.1  AT4G13960.1  AT5G12330.4  AT2G01720.1  AT1G09230.1  AT3G08900.1  AT3G28960.1  AT1G67420.2  AT4G16144.1  AT3G16850.1  AT2G20790.1  AT1G55860.2  AT3G42170.1  AT2G13100.1  AT2G24370.1  AT3G24870.1  AT2G47800.1  AT1G01220.1  AT4G03080.1  AT3G63220.2  AT1G02816.1  AT3G50700.1  AT3G20820.1  AT4G17330.1  AT1G06820.1  AT2G38470.1  AT4G22590.1  AT5G03610.1  AT3G13300.2  AT1G78520.1  AT2G15490.1  AT2G26600.1  AT1G43130.1  AT1G43890.2  AT1G72990.1  AT4G09980.1  AT1G51340.2  AT1G68470.1  AT4G17080.1  AT3G48050.2  AT1G64060.1  AT1G19000.1  AT5G17680.1  AT3G12770.1  AT1G07910.1  AT2G45910.1  AT2G30020.1  AT5G58600.1  AT3G08040.1  AT5G04530.1  AT2G13620.1  AT5G66380.1  AT2G05790.1  AT1G09460.1 | Ferrochelatase 2  Coatomer, beta subunit  ADP/ATP carrier 3  Cation exchanger 11  NAD(P)-binding Rossmann-fold superfamily protein  Nodulin MtN21 /EamA-like transporter family protein  RING/FYVE/PHD zinc finger superfamily protein  Heat shock protein 91  Subtilase family protein  F-box/RNI-like superfamily protein  Lateral root primordium (LRP) protein-related  Ribophorin I  RNA-binding (RRM/RBD/RNP motifs) family protein  reversibly glycosylated polypeptide 3  Transmembrane amino acid transporter family protein  Zn-dependent exopeptidases superfamily protein  associated molecule with the SH3 domain of STAM 3  Pectin lyase-like superfamily protein  Clathrin adaptor complexes medium subunit family protein  Ubiquitin-protein ligase 1  BED zinc finger ;hAT family dimerisation domain  Major facilitator superfamily protein  Protein kinase protein with adenine nucleotide alpha hydrolases-like domain  Helicase/SANT-associated, DNA binding protein  multidrug resistance-associated protein 4  L-fucokinase/GDP-L-fucose pyrophosphorylase  BRI1 suppressor 1 (BSU1)-like 1  Galactose oxidase/kelch repeat superfamily protein  Protein of unknown function, DUF538  Indeterminate(ID)-domain 2  Leucine-rich repeat (LRR) family protein  G2484-1 protein  Carotenoid isomerase  WRKY DNA-binding protein 33  Haloacid dehalogenase-like hydrolase (HAD) superfamily protein  GDSL-like Lipase/Acylhydrolase superfamily protein  Transducin/WD40 repeat-like superfamily protein  Carbohydrate-binding X8 domain superfamily protein  UDP-glycosyltransferase 73B4  Glycosyl hydrolase superfamily protein  Like COV 2  RAB GTPASE HOMOLOG B18  Beta-galactosidase 17  Methyltransferase MT-A70 family protein  MATE efflux family protein  Exostosin family protein  Histone H3 K4-specific methyltransferase SET7/9 family protein  BAH domain ;TFIIS helical bundle-like domain  Respiratory burst oxidase protein F  Homeodomain-like superfamily protein  Disease resistance protein (TIR-NBS-LRR class), putative  mitochondrial editing factor 22  RNAligase  U-box domain-containing protein kinase family protein  Protein phosphatase 2C family protein  Plant protein of unknown function (DUF828)  MATE efflux family protein  3-Ketoacyl-CoA synthase 19  Cation/hydrogen exchanger 15  Folate transporter 1  O-Glycosyl hydrolases family 17 protein  Carbohydrate-binding X8 domain superfamily protein |
| miR5137 | clementine0.9_005724m|PACid:19280800  clementine0.9_003428m|PACid:19276495 | AT4G34740.1  AT5G50210.1 | GLN phosphoribosyl pyrophosphate amidotransferase 2  Quinolinate synthase |
| miR5142 | clementine0.9_008621m|PACid:19255354  clementine0.9_024153m|PACid:19258096  clementine0.9_011586m|PACid:19262815  clementine0.9_000629m|PACid:19264001  clementine0.9_008957m|PACid:19264450  clementine0.9_015383m|PACid:19264452  clementine0.9_029124m|PACid:19268223  clementine0.9_008186m|PACid:19269132  clementine0.9_007728m|PACid:19270277  clementine0.9_007848m|PACid:19270161  clementine0.9_005756m|PACid:19270421  clementine0.9_035829m|PACid:19271420  clementine0.9_004187m|PACid:19272023 | AT4G36920.2  AT1G13950.1  AT3G13920.1  AT1G02890.1  AT3G63300.1  AT3G22810.1  AT1G73805.1  AT5G66680.1  AT1G76040.2  AT4G16580.1  AT1G61010.2  AT1G11710.1  AT1G49730.1 | Integrase-type DNA-binding superfamily protein  Eukaryotic elongation factor 5A-1  Eukaryotic translation initiation factor 4A1  AAA-type ATPase family protein  FORKED 1  Plant protein of unknown function (DUF828) with plant pleckstrin homology-like region  Calmodulin binding protein-like  Dolichyl-diphosphooligosaccharide-protein glycosyltransferase 48kDa subunit family protein  Calcium-dependent protein kinase 29  Protein phosphatase 2C family protein  Cleavage and polyadenylation specificity factor 73-I  Pentatricopeptide repeat (PPR) superfamily protein  Protein kinase superfamily protein |
| miR5157 | clementine0.9_000936m|PACid:19252376 | AT3G27325.2 | Hydrolases, acting on ester bonds |
| miR5161 | clementine0.9_030802m|PACid:19277027 | AT4G10850.1 | Nodulin MtN3 family protein |
| miR5176 | clementine0.9_003916m|PACid:19252091  clementine0.9_009043m|PACid:19252968  clementine0.9_031356m|PACid:19252756  clementine0.9_001975m|PACid:19253040  clementine0.9_002411m|PACid:19253715  clementine0.9_012343m|PACid:19253093  clementine0.9_012413m|PACid:19253676  clementine0.9_029955m|PACid:19254005  clementine0.9_007569m|PACid:19255395  clementine0.9_007893m|PACid:19256592  clementine0.9_001106m|PACid:19257071  clementine0.9_029430m|PACid:19257303  clementine0.9_000547m|PACid:19257728  clementine0.9_030953m|PACid:19258920  clementine0.9_002776m|PACid:19259576  clementine0.9_003094m|PACid:19260206  clementine0.9_022825m|PACid:19261173  clementine0.9_027498m|PACid:19262237  clementine0.9_000075m|PACid:19263927  clementine0.9_002649m|PACid:19265262  clementine0.9_004426m|PACid:19263851  clementine0.9_010793m|PACid:19263435  clementine0.9_010731m|PACid:19264971  clementine0.9_017738m|PACid:19265220  clementine0.9_000278m|PACid:19261813  clementine0.9_012633m|PACid:19268034  clementine0.9_021601m|PACid:19267805  clementine0.9_028559m|PACid:19267885  clementine0.9_002341m|PACid:19268931  clementine0.9_029022m|PACid:19269503  clementine0.9_002519m|PACid:19269784  clementine0.9_006221m|PACid:19269903  clementine0.9_009143m|PACid:19270100  clementine0.9_020574m|PACid:19271361  clementine0.9_033119m|PACid:19270685  clementine0.9_006705m|PACid:19271694  clementine0.9_009390m|PACid:19272068  clementine0.9_035854m|PACid:19272263  clementine0.9_015074m|PACid:19273531  clementine0.9_028966m|PACid:19273547  clementine0.9_007003m|PACid:19273065  clementine0.9_010145m|PACid:19272806  clementine0.9_010999m|PACid:19274106  clementine0.9_014797m|PACid:19274236  clementine0.9_003438m|PACid:19275006  clementine0.9_017091m|PACid:19275394  clementine0.9_027709m|PACid:19275520  clementine0.9_028834m|PACid:19276331  clementine0.9_007361m|PACid:19277357  clementine0.9_013141m|PACid:19277371  clementine0.9_034005m|PACid:19279442  clementine0.9_000623m|PACid:19279659  clementine0.9_014363m|PACid:19281558  clementine0.9_009638m|PACid:19282470  clementine0.9_001787m|PACid:19283147  clementine0.9_003487m|PACid:19283680  clementine0.9_001148m|PACid:19284776  clementine0.9_007080m|PACid:19284825  clementine0.9_006470m|PACid:19286303  clementine0.9_007240m|PACid:19286112  clementine0.9_029339m|PACid:19286698  clementine0.9_030919m|PACid:19286595  clementine0.9_029253m|PACid:19286467  clementine0.9_007277m|PACid:19286822 | AT1G03530.1  AT1G31070.2  AT5G44800.1  AT1G77860.1  AT1G22260.1  AT4G27190.1  AT1G31500.1  AT1G63350.1  AT5G02970.1  AT4G02600.2  AT5G20280.1  AT2G17030.1  AT5G43310.2  AT5G63020.1  AT2G04030.1  AT1G78950.1  AT3G16990.1  AT1G19250.1  AT3G24870.1  AT4G02280.1  AT3G14730.1  AT1G01920.2  AT3G62770.1  AT5G27690.1  AT3G14460.1  AT3G11945.1  AT5G01750.2  AT1G04110.1  AT2G42010.1  AT5G64530.1  AT4G24190.1  AT5G42740.1  AT5G57030.1  AT4G05180.1  AT4G05200.1  AT1G76160.1  AT3G19680.1  AT2G17410.1  AT1G15950.1  AT2G25680.1  AT1G21660.1  AT1G21200.1  AT4G13840.1  AT5G24230.1  AT2G41900.1  AT3G12800.1  AT5G51780.1  AT4G17080.1  AT5G14540.1  AT5G41970.1  AT2G32350.1  AT1G08620.1  AT1G65410.1  AT3G09690.1  AT5G54730.1  AT5G51600.1  AT4G35560.1  AT4G36860.1  AT1G11580.1  AT2G02170.1  AT2G24030.2  AT2G19380.1  AT2G21480.1  AT3G52990.1 | Nuclear assembly factor 1  N-acetylglucosamine-1-phosphate uridylyltransferase 1  Chromatin remodeling 4  Myosin heavy chain-related protein  NB-ARC domain-containing disease resistance protein  Rhomboid-related intramembrane serine protease family protein  DNAse I-like superfamily protein  Disease resistance protein (CC-NBS-LRR class) family  Alpha/beta-Hydrolases superfamily protein  Seven transmembrane MLO family protein  Sucrose phosphate synthase 1F  F-box family protein with a domain of unknown function (DUF295)  COP1-interacting protein-related  Disease resistance protein (CC-NBS-LRR class) family  Chaperone protein htpG family protein  Terpenoid cyclases family protein  Haem oxygenase-like, multi-helical  Flavin-dependent monooxygenase 1  Helicase/SANT-associated, DNA binding protein  Sucrose synthase 3  Pentatricopeptide repeat (PPR) superfamily protein  SET domain-containing protein  Transducin/WD40 repeat-like superfamily protein  Heavy metal transport/detoxification superfamily protein  LRR and NB-ARC domains-containing disease resistance protein  Homogentisate prenyltransferase  Protein of unknown function (DUF567)  Subtilase family protein  Phospholipase D beta 1  Xylem NAC domain 1  Chaperone protein htpG family protein  Sugar isomerase (SIS) family protein  Lycopene beta/epsilon cyclase protein  Photosystem II subunit Q-2  Cysteine-rich RLK (RECEPTOR-like protein kinase) 25  SKU5 similar 5  Protein of unknown function (DUF1005)  ARID/BRIGHT DNA-binding domain-containing protein  Cinnamoyl coa reductase 1  Molybdate transporter 1  Chaperone DnaJ-domain superfamily protein  Sequence-specific DNA binding transcription factors  HXXXD-type acyl-transferase family protein  Lipase class 3-related protein  CCCH-type zinc finger protein with ARM repeat domain  Short-chain dehydrogenase-reductase B  Basic helix-loop-helix (bHLH) DNA-binding superfamily protein  Histone H3 K4-specific methyltransferase SET7/9 family protein  Protein of unknown function (DUF1421)  Metal-dependent protein hydrolase  Ubiquitin-like superfamily protein  Transcription factor jumonji (jmj) family protein / zinc finger (C5HC2 type) family protein  Non-intrinsic ABC protein 11  Alpha/beta-Hydrolases superfamily protein  Homolog of yeast autophagy 18 (ATG18) F  Microtubule associated protein (MAP65/ASE1) family protein  Transducin/WD40 repeat-like superfamily protein  LIM domain-containing protein  Methylesterase PCR A  Remorin family protein  zinc ion binding;nucleic acid binding  RNA recognition motif (RRM)-containing protein  Malectin/receptor-like protein kinase family protein  Pyruvate kinase family protein |
| miR5181 | clementine0.9_012247m|PACid:19259861  clementine0.9_004893m|PACid:19259474  clementine0.9_019300m|PACid:19263403  clementine0.9_005212m|PACid:19270225  clementine0.9_006918m|PACid:19269751  clementine0.9_004372m|PACid:19272309  clementine0.9_022715m|PACid:19272671  clementine0.9_028767m|PACid:19274534  clementine0.9_014386m|PACid:19279846  clementine0.9_004287m|PACid:19280565  clementine0.9_018047m|PACid:19284183  clementine0.9_009895m|PACid:19287111 | AT4G23660.1  AT1G25570.1  AT5G15270.2  AT1G76400.1  AT1G19880.1  AT3G18230.1  AT4G09510.1  AT4G33360.1  AT5G54160.1  AT2G31060.2  AT2G16050.1  AT1G28110.2 | Polyprenyltransferase 1  Di-glucose binding protein with Leucine-rich repeat domain  RNA-binding KH domain-containing protein  Ribophorin I  Regulator of chromosome condensation (RCC1) family protein  Octicosapeptide/Phox/Bem1p family protein  Cytosolic invertase 2  NAD(P)-binding Rossmann-fold superfamily protein  O-methyltransferase 1  Elongation factor family protein  Cysteine/Histidine-rich C1 domain family protein  Serine carboxypeptidase-like 45 |
| miR5204 | clementine0.9_030283m|PACid:19256019  clementine0.9_006859m|PACid:19252078  clementine0.9_034535m|PACid:19258602  clementine0.9_005260m|PACid:19259641  clementine0.9_016310m|PACid:19259666  clementine0.9_036083m|PACid:19265035  clementine0.9_001029m|PACid:19265978  clementine0.9_008940m|PACid:19268058  clementine0.9_014716m|PACid:19271512  clementine0.9_029911m|PACid:19272222  clementine0.9_005578m|PACid:19279409 | AT4G08850.1  AT3G01710.2  AT3G61460.1  AT1G70170.1  AT1G26460.1  AT2G02880.1  AT2G20050.1  AT1G62620.1  AT1G30040.1  AT3G19220.1  AT1G06560.1 | Leucine-rich repeat receptor-like protein kinase family protein  TPX2 (targeting protein for Xklp2) protein family  Matrix metalloproteinase  Tetratricopeptide repeat (TPR)-like superfamily protein  Mucin-related  Brassinosteroid-responsive RING-H2  protein serine/threonine phosphatases;protein kinases;catalytics;cAMP-dependent protein kinase regulators;ATP binding;protein serine/threonine phosphatases  Flavin-binding monooxygenase family protein  Gibberellin 2-oxidase  Protein disulfide isomerases  NOL1/NOP2/sun family protein |
| miR5207 | clementine0.9_005103m|PACid:19268818  clementine0.9_004807m|PACid:19284096 | AT5G16000.1  AT5G58300.1 | NSP-interacting kinase 1  Leucine-rich repeat protein kinase family protein |
| miR5209 | clementine0.9_012330m|PACid:19254644  clementine0.9_013933m|PACid:19254425  clementine0.9_032125m|PACid:19253513  clementine0.9_006051m|PACid:19256080  clementine0.9_014922m|PACid:19256349  clementine0.9_010434m|PACid:19257866  clementine0.9_000193m|PACid:19258770  clementine0.9_011475m|PACid:19259454  clementine0.9_028839m|PACid:19259059  clementine0.9_001899m|PACid:19261036  clementine0.9_020567m|PACid:19260962  clementine0.9_007220m|PACid:19262531  clementine0.9_027970m|PACid:19262877  clementine0.9_000089m|PACid:19264721  clementine0.9_000187m|PACid:19264206  clementine0.9_000933m|PACid:19265014  clementine0.9_004945m|PACid:19264665  clementine0.9_007229m|PACid:19263861  clementine0.9_035706m|PACid:19263887  clementine0.9_030671m|PACid:19266518  clementine0.9_000512m|PACid:19267919  clementine0.9_004998m|PACid:19270828  clementine0.9_004835m|PACid:19273814  clementine0.9_004053m|PACid:19276575  clementine0.9_001064m|PACid:19278221  clementine0.9_005826m|PACid:19278132  clementine0.9_019514m|PACid:19280913  clementine0.9_029769m|PACid:19283053  clementine0.9_002903m|PACid:19286002  clementine0.9_008304m|PACid:19287087  clementine0.9_010590m|PACid:19286793 | AT3G05210.1  AT3G62770.1  AT1G66920.1  AT3G46730.1  AT4G32890.1  AT5G08130.3  AT2G13680.1  AT2G03620.2  AT3G48460.1  AT2G37670.1  AT5G18650.1  AT5G20680.3  AT5G57620.1  AT1G01040.1  AT2G47410.1  AT5G19820.1  AT2G31270.1  AT3G06720.1  AT4G13180.1  AT1G44224.1  AT3G08850.1  AT5G57580.1  AT5G25630.1  AT5G50310.1  AT4G34830.1  AT2G21140.1  AT5G53110.1  AT5G35450.1  AT2G27460.1  AT5G17300.1  AT1G36730.1 | nucleotide repair protein, putative  Transducin/WD40 repeat-like superfamily protein  Protein kinase superfamily protein  NB-ARC domain-containing disease resistance protein  GATA transcription factor 9  Basic helix-loop-helix (bHLH) DNA-binding superfamily protein  Callose synthase 5  Magnesium transporter 3  GDSL-like Lipase/Acylhydrolase superfamily protein  Transducin/WD40 repeat-like superfamily protein  CHY-type/CTCHY-type/RING-type Zinc finger protein  TRICHOME BIREFRINGENCE-LIKE 16  Myb domain protein 36  Dicer-like 1  WD40/YVTN repeat-like-containing domain;Bromodomain  ARM repeat superfamily protein  Homolog of yeast CDT1 A  Importin alpha isoform 1  NAD(P)-binding Rossmann-fold superfamily protein  ECA1 gametogenesis related family protein  HEAT repeat ;WD domain, G-beta repeat protein protein  Calmodulin-binding protein  Tetratricopeptide repeat (TPR)-like superfamily protein  Galactose oxidase/kelch repeat superfamily protein  Pentatricopeptide repeat (PPR) superfamily protein  Proline-rich protein 2  RING/U-box superfamily protein  Disease resistance protein (CC-NBS-LRR class) family  Sec23/sec24 transport family protein  Homeodomain-like superfamily protein  Translation initiation factor IF2/IF5 |
| miR5211 | clementine0.9_008584m|PACid:19259523  clementine0.9_002020m|PACid:19265883 | AT1G60770.1  AT3G47570.1 | Tetratricopeptide repeat (TPR)-like superfamily protein  Leucine-rich repeat protein kinase family protein |
| miR5224 | clementine0.9_034152m|PACid:19251567  clementine0.9_002788m|PACid:19252885  clementine0.9_033465m|PACid:19253677  clementine0.9_002877m|PACid:19263370  clementine0.9_032921m|PACid:19267234  clementine0.9_006962m|PACid:19270381  clementine0.9_019284m|PACid:19272923  clementine0.9_003165m|PACid:19274390  clementine0.9_004968m|PACid:19277340  clementine0.9_009664m|PACid:19276962  clementine0.9_011999m|PACid:19277782  clementine0.9_011886m|PACid:19279010  clementine0.9_019768m|PACid:19279047  clementine0.9_034478m|PACid:19279273  clementine0.9_000042m|PACid:19283676 | AT1G69640.1  AT2G35060.1  AT1G68210.1  AT5G17930.1  AT3G11960.1  AT4G20820.1  AT1G14290.1  AT3G13990.2  AT1G33170.1  AT1G64650.1  AT1G07160.1  AT4G19660.1  AT3G10260.3  AT5G45110.1  AT1G16800.1 | Sphingoid base hydroxylase 1  K+ uptake permease 11  Pseudo-response regulator 6  MIF4G domain-containing protein / MA3 domain-containing protein  Cleavage and polyadenylation specificity factor (CPSF) A subunit protein  FAD-binding Berberine family protein  Sphingoid base hydroxylase 2  Kinase-related protein of unknown function (DUF1296)  S-adenosyl-L-methionine-dependent methyltransferases superfamily protein  Major facilitator superfamily protein  Protein phosphatase 2C family protein  NPR1-like protein 4  Reticulon family protein  NPR1-like protein 3  P-loop containing nucleoside triphosphate hydrolases superfamily protein |
| miR5245 | clementine0.9_016723m|PACid:19264729  clementine0.9_005234m|PACid:19275864  clementine0.9_004256m|PACid:19287105 | AT4G28530.1  AT5G53130.1  AT2G36570.1 | NAC domain containing protein 74  Cyclic nucleotide gated channel 1  Leucine-rich repeat protein kinase family protein |
| miR5266 | clementine0.9_001451m|PACid:19252008  clementine0.9_012893m|PACid:19253629  clementine0.9_001501m|PACid:19256069  clementine0.9_008179m|PACid:19257317  clementine0.9_005589m|PACid:19262393  clementine0.9_021305m|PACid:19262028  clementine0.9_028439m|PACid:19263670  clementine0.9_031803m|PACid:19264063  clementine0.9_004507m|PACid:19269095  clementine0.9_004372m|PACid:19272309  clementine0.9_005242m|PACid:19273262  clementine0.9_005335m|PACid:19273263  clementine0.9_003626m|PACid:19277462  clementine0.9_001448m|PACid:19280349  clementine0.9_033201m|PACid:19280965 | AT2G25140.1  AT4G08960.1  AT5G14580.1  AT4G13510.1  AT4G16850.1  AT2G06000.1  AT1G02550.1  AT3G06260.1  AT2G21710.1  AT3G18230.1  AT1G51580.1  AT4G25390.1  AT5G50280.1  AT2G41740.1  AT5G40940.1 | Casein lytic proteinase B4  Phosphotyrosyl phosphatase activator (PTPA) family protein  Polyribonucleotide nucleotidyltransferase, putative  Ammonium transporter 1;1  Pentatricopeptide repeat (PPR) superfamily protein  Uncharacterised protein family (UPF0172)  Plant invertase/pectin methylesterase inhibitor superfamily protein  Galacturonosyltransferase-like 4  Mitochondrial transcription termination factor family protein  Octicosapeptide/Phox/Bem1p family protein  RNA-binding KH domain-containing protein  Protein kinase superfamily protein  Pentatricopeptide repeat (PPR) superfamily protein  Villin 2  Putative fasciclin-like arabinogalactan protein 20 |
| miR5340 | clementine0.9_007657m|PACid:19254202  clementine0.9_011291m|PACid:19254652  clementine0.9_003259m|PACid:19263015  clementine0.9_016605m|PACid:19267386  clementine0.9_010578m|PACid:19268246  clementine0.9_027870m|PACid:19268824  clementine0.9_035721m|PACid:19273278  clementine0.9_010257m|PACid:19276484  clementine0.9_014886m|PACid:19279147  clementine0.9_004672m|PACid:19281864  clementine0.9_019675m|PACid:19286045 | AT4G31500.1  AT1G32240.1  AT4G30200.3  AT4G22810.1  AT4G23180.1  AT1G15000.1  AT5G56840.1  AT5G62810.1  AT4G18170.1  AT3G13060.2  AT2G27480.1 | Cytochrome P450, family 83, subfamily B, polypeptide 1  Homeodomain-like superfamily protein  Vernalization5/VIN3-like  Predicted AT-hook DNA-binding family protein  Cysteine-rich RLK (RECEPTOR-like protein kinase) 10  Serine carboxypeptidase-like 50  Myb-like transcription factor family protein  Peroxin 14  WRKY DNA-binding protein 28  Evolutionarily conserved C-terminal region 5  Calcium-binding EF-hand family protein |
| miR535 | clementine0.9_007376m|PACid:19284054  clementine0.9_029142m|PACid:19265294 | AT5G24910.1  AT4G00590.1 | Cytochrome P450, family 714, subfamily A, polypeptide 1  N-terminal nucleophile aminohydrolases (Ntn hydrolases) superfamily protein |
| miR5386 | clementine0.9_018473m|PACid:19278117 | AT2G21190.1 | ER lumen protein retaining receptor family protein |
| miR5473 | clementine0.9_023824m|PACid:19280041  clementine0.9_003600m|PACid:19254954  clementine0.9_005938m|PACid:19262762 | AT5G51010.1  AT1G69730.1  AT4G03500.1 | Rubredoxin-like superfamily protein  Wall-associated kinase family protein  Ankyrin repeat family protein |
| miR5485 | clementine0.9_001686m|PACid:19258853  clementine0.9_002266m|PACid:19252555  clementine0.9_022613m|PACid:19252536  clementine0.9_027657m|PACid:19253609  clementine0.9_025735m|PACid:19266930  clementine0.9_001026m|PACid:19272099  clementine0.9_011670m|PACid:19277726  clementine0.9_035652m|PACid:19279089  clementine0.9_009638m|PACid:19282470 | AT3G10690.1  AT4G39530.1  AT4G26240.1  AT5G53110.1  AT3G53980.2  AT1G49630.3  AT5G41670.2  AT4G21790.1  AT3G09690.1 | DNA GYRASE A  Tetratricopeptide repeat (TPR)-like superfamily protein  RING/U-box superfamily protein  Bifunctional inhibitor/lipid-transfer protein/seed storage 2S albumin superfamily protein  5\'-3\' exonuclease family protein  Presequence protease 2  6-Phosphogluconate dehydrogenase family protein  Tobamovirus multiplication 1  Alpha/beta-Hydrolases superfamily protein |
| miR5491 | clementine0.9_031498m|PACid:19251322  clementine0.9_002377m|PACid:19252519  clementine0.9_032358m|PACid:19252489  clementine0.9_000405m|PACid:19252472  clementine0.9_000695m|PACid:19254043  clementine0.9_000810m|PACid:19255464  clementine0.9_012138m|PACid:19255526  clementine0.9_003038m|PACid:19256116  clementine0.9_015320m|PACid:19256165  clementine0.9_012645m|PACid:19256727  clementine0.9_031443m|PACid:19256559  clementine0.9_010445m|PACid:19257173  clementine0.9_035604m|PACid:19257098  clementine0.9_022065m|PACid:19257785  clementine0.9_034209m|PACid:19257677  clementine0.9_002707m|PACid:19259544  clementine0.9_003250m|PACid:19259365  clementine0.9_010604m|PACid:19260672  clementine0.9_029364m|PACid:19260665  clementine0.9_000228m|PACid:19263080  clementine0.9_031995m|PACid:19262777  clementine0.9_000075m|PACid:19263927  clementine0.9_010206m|PACid:19264834  clementine0.9_016372m|PACid:19264618  clementine0.9_033227m|PACid:19263846  clementine0.9_034955m|PACid:19265227  clementine0.9_000925m|PACid:19265716  clementine0.9_009243m|PACid:19265635  clementine0.9_033871m|PACid:19265612  clementine0.9_003954m|PACid:19267442  clementine0.9_004261m|PACid:19266709  clementine0.9_006126m|PACid:19267933  clementine0.9_013976m|PACid:19267339  clementine0.9_027747m|PACid:19267385  clementine0.9_032301m|PACid:19267194  clementine0.9_018467m|PACid:19268773  clementine0.9_036060m|PACid:19268747  clementine0.9_009096m|PACid:19270209  clementine0.9_004892m|PACid:19270990  clementine0.9_005726m|PACid:19271016  clementine0.9_011962m|PACid:19271534  clementine0.9_004187m|PACid:19272023  clementine0.9_010406m|PACid:19272153  clementine0.9_022439m|PACid:19272087  clementine0.9_005466m|PACid:19274402  clementine0.9_007308m|PACid:19274756  clementine0.9_010745m|PACid:19274734  clementine0.9_034995m|PACid:19274396  clementine0.9_000544m|PACid:19275427  clementine0.9_030823m|PACid:19275489  clementine0.9_010460m|PACid:19276062  clementine0.9_000826m|PACid:19276690  clementine0.9_008867m|PACid:19276501  clementine0.9_022517m|PACid:19277068  clementine0.9_035143m|PACid:19277710  clementine0.9_013935m|PACid:19278099  clementine0.9_030444m|PACid:19278167  clementine0.9_001683m|PACid:19278541  clementine0.9_003260m|PACid:19282634  clementine0.9_028618m|PACid:19282199  clementine0.9_022025m|PACid:19282981  clementine0.9_028125m|PACid:19282910  clementine0.9_013020m|PACid:19283453  clementine0.9_020572m|PACid:19283371  clementine0.9_020257m|PACid:19283490  clementine0.9_018111m|PACid:19284188  clementine0.9_000004m|PACid:19285010  clementine0.9_019140m|PACid:19284986  clementine0.9_001384m|PACid:19285949  clementine0.9_019978m|PACid:19286199  clementine0.9_004581m|PACid:19286589  clementine0.9_005015m|PACid:19287027 | AT1G47530.1  AT3G18370.1  AT3G47890.1  AT4G27190.1  AT1G12220.1  AT4G27190.1  AT4G12830.1  AT1G08520.1  AT4G31530.1  AT5G18270.2  AT4G21390.1  AT4G32330.2  AT4G32551.2  AT2G31160.1  AT4G14440.1  AT1G13170.1  AT1G05460.1  AT1G03220.1  AT4G16280.4  AT3G43920.2  AT4G10310.1  AT3G24870.1  AT4G01680.1  AT2G38830.1  AT4G28520.1  AT2G16760.1  AT5G19820.1  AT3G13620.1  AT2G03090.1  AT2G35940.3  AT3G09070.1  AT1G09810.1  AT4G13880.1  AT1G53350.1  AT2G36090.1  AT1G73830.1  AT3G51550.1  AT5G66730.1  AT4G23180.1  AT5G54960.1  AT2G34560.2  AT1G49730.1  AT3G06240.1  AT1G10200.1  AT1G72790.1  AT1G47670.1  AT1G47490.1  AT5G45480.1  AT4G20850.1  AT1G30870.1  AT5G58330.2  AT3G48195.1  AT4G20360.1  AT1G74730.1  AT5G17680.1  AT2G16630.1  AT1G68930.1  AT5G35980.1  AT2G32910.1  AT5G22890.1  AT2G44740.1  AT5G27740.1  AT3G48800.1  AT3G60910.1  AT5G13790.1  AT2G40200.1  AT2G17930.1  AT4G36130.1  AT1G22860.1  AT5G44560.1  AT5G41120.1  AT1G70530.1 | MATE efflux family protein  C2 domain-containing protein  Ubiquitin carboxyl-terminal hydrolase-related protein  NB-ARC domain-containing disease resistance protein  Disease resistance protein (CC-NBS-LRR class) family  Homeodomain-like transcriptional regulator  Alpha/beta-Hydrolases superfamily protein  ALBINA 1  NAD(P)-binding Rossmann-fold superfamily protein  Arabidopsis NAC domain containing protein 87  S-locus lectin protein kinase family protein  TPX2 (targeting protein for Xklp2) protein family  LisH dimerisation motif;WD40/YVTN repeat-like-containing domain  Protein of unknown function (DUF640)  3-Hydroxyacyl-CoA dehydratase 1  OSBP(oxysterol binding protein)-related protein 1D  P-loop containing nucleoside triphosphate hydrolases superfamily protein  Eukaryotic aspartyl protease family protein  RNA binding;abscisic acid binding  Dicer-like 3  High-affinity K+ transporter 1  Helicase/SANT-associated, DNA binding protein  Myb domain protein 55  Ubiquitin-conjugating enzyme/RWD-like protein  Cruciferin 3  Calcium-dependent phosphotriesterase superfamily protein  ARM repeat superfamily protein  Amino acid permease family protein  Expansin A15  BEL1-like homeodomain 1  Protein of unknown function (DUF740)  Evolutionarily conserved C-terminal region 11  Receptor like protein 48  Disease resistance protein (CC-NBS-LRR class) family  F-box family protein  BR enhanced expression 3  Malectin/receptor-like protein kinase family protein  C2H2-like zinc finger protein  Cysteine-rich RLK (RECEPTOR-like protein kinase) 10  pyruvate decarboxylase-2  P-loop containing nucleoside triphosphate hydrolases superfamily protein  Protein kinase superfamily protein  F-box family protein  GATA type zinc finger transcription factor family protein  Hydroxyproline-rich glycoprotein family protein  Transmembrane amino acid transporter family protein  RNA-binding protein 47C  Protein of unknown function (DUF594)  Tripeptidyl peptidase ii  Peroxidase superfamily protein  Lactate/malate dehydrogenase family protein  Phox (PX) domain-containing protein  RAB GTPase homolog E1B  Protein of unknown function (DUF1118)  Disease resistance protein (TIR-NBS-LRR class), putative  Pollen Ole e 1 allergen and extensin family protein  Pentatricopeptide (PPR) repeat-containing protein  Yeast YAK1-related gene 1  DCD (Development and Cell Death) domain protein  C2H2 and C2HC zinc fingers superfamily protein  Cyclin p4;1  ATPase family associated with various cellular activities (AAA)  Sterile alpha motif (SAM) domain-containing protein  S-adenosyl-L-methionine-dependent methyltransferases superfamily protein  AGAMOUS-like 15  Basic helix-loop-helix (bHLH) DNA-binding superfamily protein  Phosphatidylinositol 3- and 4-kinase family protein with FAT domain  Ribosomal protein L2 family  Vacuolar sorting protein 39  SNF7 family protein  Esterase/lipase/thioesterase family protein  Cysteine-rich RLK (RECEPTOR-like protein kinase) 3 |
| miR5492 | clementine0.9_011827m|PACid:19266423  clementine0.9_001828m|PACid:19255555  clementine0.9_029460m|PACid:19265771  clementine0.9_033175m|PACid:19269322  clementine0.9_013560m|PACid:19272778  clementine0.9_010613m|PACid:19274024  clementine0.9_022259m|PACid:19275326  clementine0.9_005617m|PACid:19276149  clementine0.9_022368m|PACid:19276618  clementine0.9_012346m|PACid:19280192  clementine0.9_035470m|PACid:19284441 | AT1G34300.1  AT4G12640.1  AT3G45140.1  AT4G33550.2  AT1G43650.1  AT1G78850.1  AT5G51545.1  AT2G28120.1  AT3G48100.1  AT3G10140.1  AT3G55950.1 | Lectin protein kinase family protein  RNA recognition motif (RRM)-containing protein  Lipoxygenase 2  Bifunctional inhibitor/lipid-transfer protein/seed storage 2S albumin superfamily protein  Nodulin MtN21 /EamA-like transporter family protein  D-mannose binding lectin protein with Apple-like carbohydrate-binding domain  Low psii accumulation2  Major facilitator superfamily protein  response regulator 5  RECA homolog 3  CRINKLY4 related 3 |
| miR5519 | clementine0.9_010672m|PACid:19253408  clementine0.9_031764m|PACid:19252041  clementine0.9_029473m|PACid:19254804  clementine0.9_009840m|PACid:19255498  clementine0.9_029248m|PACid:19256763  clementine0.9_007982m|PACid:19257605  clementine0.9_017385m|PACid:19257667  clementine0.9_019486m|PACid:19257584  clementine0.9_001282m|PACid:19257821  clementine0.9_010848m|PACid:19260068  clementine0.9_031761m|PACid:19259083  clementine0.9_016991m|PACid:19260742  clementine0.9_013104m|PACid:19260545  clementine0.9_027773m|PACid:19261417  clementine0.9_006987m|PACid:19262931  clementine0.9_007349m|PACid:19263301  clementine0.9_008414m|PACid:19262138  clementine0.9_002839m|PACid:19263980  clementine0.9_015583m|PACid:19264801  clementine0.9_030396m|PACid:19264928  clementine0.9_000087m|PACid:19265826  clementine0.9_027425m|PACid:19265760  clementine0.9_025770m|PACid:19265958  clementine0.9_036009m|PACid:19266605  clementine0.9_006754m|PACid:19268839  clementine0.9_007516m|PACid:19269545  clementine0.9_016601m|PACid:19270246  clementine0.9_030832m|PACid:19272307  clementine0.9_028986m|PACid:19274039  clementine0.9_004938m|PACid:19275470  clementine0.9_007741m|PACid:19276108  clementine0.9_000651m|PACid:19277037  clementine0.9_033496m|PACid:19277041  clementine0.9_034430m|PACid:19277560  clementine0.9_001807m|PACid:19279171  clementine0.9_000415m|PACid:19280133  clementine0.9_010379m|PACid:19281563  clementine0.9_033941m|PACid:19282612  clementine0.9_006992m|PACid:19284168  clementine0.9_013118m|PACid:19283905  clementine0.9_024865m|PACid:19283847  clementine0.9_000942m|PACid:19285674  clementine0.9_011361m|PACid:19287061 | AT1G77670.1  AT2G34930.1  AT3G18660.1  AT4G12740.1  AT3G28860.1  AT2G26640.1  AT1G04520.1  AT3G22490.1  AT1G69360.1  AT3G04120.1  AT1G23760.1  AT2G47900.3  AT2G37620.1  AT3G47570.1  AT5G35360.1  AT4G33220.1  AT4G28070.2  AT3G03770.2  AT4G02440.1  AT5G48890.1  AT1G55325.2  AT3G06880.2  AT1G60660.1  AT5G01550.1  AT1G67180.1  AT4G27500.1  AT1G75280.1  AT3G53140.1  AT1G78850.1  AT5G52860.1  AT1G80910.1  AT1G33390.1  AT1G70140.1  AT3G14460.1  AT5G17790.1  AT2G41520.1  AT3G04680.2  AT3G11340.1  AT5G05980.2  AT3G12110.1  AT2G41430.5  AT2G05120.1  AT1G09700.1 | Pyridoxal phosphate (PLP)-dependent transferases superfamily protein  Disease resistance family protein / LRR family protein  Plant glycogenin-like starch initiation protein 1  HhH-GPD base excision DNA repair family protein  ATP binding cassette subfamily B19  3-Ketoacyl-CoA synthase 11  Plasmodesmata-located protein 2  Seed maturation protein  Plant protein of unknown function (DUF863)  Glyceraldehyde-3-phosphate dehydrogenase C subunit 1  BURP domain-containing protein  Tubby like protein 3  Actin 1  Leucine-rich repeat protein kinase family protein  Acetyl Co-enzyme a carboxylase biotin carboxylase subunit  Pectin methylesterase 44  AFG1-like ATPase family protein  Leucine-rich repeat protein kinase family protein  F-box family protein  C2H2-like zinc finger protein  RNA polymerase II transcription mediators  Transducin/WD40 repeat-like superfamily protein  Cytochrome B5-like protein  Lectin receptor kinase a4.1  Zinc finger (C3HC4-type RING finger) family protein / BRCT domain-containing protein  Proton pump interactor 1  NmrA-like negative transcriptional regulator family protein  O-methyltransferase family protein  D-mannose binding lectin protein with Apple-like carbohydrate-binding domain  ABC-2 type transporter family protein  Protein of unknown function (DUF1712)  RNA helicase family protein  Formin 8  LRR and NB-ARC domains-containing disease resistance protein  Zinc finger (Ran-binding) family protein  Heat shock protein DnaJ with tetratricopeptide repeat  CLP-similar protein 3  UDP-Glycosyltransferase superfamily protein  DHFS-FPGS homolog B  Actin-11  Dehydration-induced protein (ERD15)  Nucleoporin, Nup133/Nup155-like  DsRNA-binding domain-like superfamily protein |
| miR5522 | clementine0.9_023821m|PACid:19256954  clementine0.9_033665m|PACid:19255405  clementine0.9_008925m|PACid:19258018  clementine0.9_015682m|PACid:19259451  clementine0.9_020085m|PACid:19259894  clementine0.9_011348m|PACid:19261469  clementine0.9_019445m|PACid:19262125  clementine0.9_000196m|PACid:19265021  clementine0.9_022837m|PACid:19265646  clementine0.9_007549m|PACid:19268873  clementine0.9_006151m|PACid:19276423  clementine0.9_002804m|PACid:19284503 | AT5G11260.1  AT4G08850.1  AT4G23060.1  AT1G68810.1  AT4G27670.1  AT1G72190.1  AT5G21920.1  AT3G62900.1  AT1G55260.1  AT5G15740.1  AT4G16835.1  AT1G75850.1 | Basic-leucine zipper (bZIP) transcription factor family protein  Leucine-rich repeat receptor-like protein kinase family protein  IQ-domain 22  Basic helix-loop-helix (bHLH) DNA-binding superfamily protein  Heat shock protein 21  D-isomer specific 2-hydroxyacid dehydrogenase family protein  YGGT family protein  CW-type Zinc Finger  Bifunctional inhibitor/lipid-transfer protein/seed storage 2S albumin superfamily protein  O-fucosyltransferase family protein  Tetratricopeptide repeat (TPR)-like superfamily protein  VPS35 homolog B |
| miR5534 | clementine0.9_034528m|PACid:19281322  clementine0.9_032533m|PACid:19281403 | AT3G50950.2  AT3G14470.1 | HOPZ-ACTIVATED RESISTANCE 1  NB-ARC domain-containing disease resistance protein |
| miR5554 | clementine0.9_004374m|PACid:19252800  clementine0.9_034432m|PACid:19251608  clementine0.9_002398m|PACid:19254056  clementine0.9_036073m|PACid:19256230  clementine0.9_004876m|PACid:19268200  clementine0.9_034398m|PACid:19268398  clementine0.9_029669m|PACid:19268962  clementine0.9_001232m|PACid:19269480  clementine0.9_010494m|PACid:19269618  clementine0.9_002853m|PACid:19270680  clementine0.9_001990m|PACid:19272183  clementine0.9_005411m|PACid:19271874  clementine0.9_016196m|PACid:19272962  clementine0.9_022715m|PACid:19272671  clementine0.9_025556m|PACid:19276032  clementine0.9_015658m|PACid:19279844  clementine0.9_005429m|PACid:19280832 | AT4G20090.1  AT3G07810.2  AT4G19440.1  AT5G40400.1  AT3G14630.1  AT1G12300.1  AT3G50930.1  AT3G12280.2  AT5G62030.1  AT5G59810.1  AT2G17140.1  AT1G12700.1  AT1G22700.1  AT4G09510.1  AT1G14760.2  AT2G43120.1  AT1G12775.1 | Pentatricopeptide repeat (PPR) superfamily protein  RNA-binding (RRM/RBD/RNP motifs) family protein  Tetratricopeptide repeat (TPR)-like superfamily protein  Pentatricopeptide repeat (PPR) superfamily protein  Cytochrome P450, family 72, subfamily A, polypeptide 9  Tetratricopeptide repeat (TPR)-like superfamily protein  Cytochrome BC1 synthesis  Retinoblastoma-related 1  Diphthamide synthesis DPH2 family protein  Subtilase family protein  Pentatricopeptide repeat (PPR) superfamily protein  ATP binding;nucleic acid binding;helicases  Tetratricopeptide repeat (TPR)-like superfamily protein  Cytosolic invertase 2  KNOX Arabidopsis thaliana meinox  RmlC-like cupins superfamily protein  Pentatricopeptide repeat (PPR) superfamily protein |
| miR5559 | clementine0.9_000009m|PACid:19254084  clementine0.9_010637m|PACid:19264428  clementine0.9_004670m|PACid:19266008  clementine0.9_000069m|PACid:19272713  clementine0.9_020601m|PACid:19276405  clementine0.9_000112m|PACid:19282946 | AT1G70320.1  AT3G03740.1  AT4G26300.1  AT1G77300.1  AT3G47670.1  AT3G60240.2 | Ubiquitin-protein ligase 2  BTB-POZ and MATH domain 4  Arginyl-tRNA synthetase, class Ic  Histone methyltransferases(H3-K4 specific);histone methyltransferases(H3-K36 specific)  Plant invertase/pectin methylesterase inhibitor superfamily protein  Eukaryotic translation initiation factor 4G |
| miR5562 | clementine0.9_017051m|PACid:19251737  clementine0.9_013285m|PACid:19251286  clementine0.9_023144m|PACid:19252212  clementine0.9_035845m|PACid:19252044  clementine0.9_007389m|PACid:19252679  clementine0.9_022650m|PACid:19253412  clementine0.9_023809m|PACid:19253372  clementine0.9_015320m|PACid:19256165  clementine0.9_028499m|PACid:19256064  clementine0.9_006376m|PACid:19258174  clementine0.9_010434m|PACid:19257866  clementine0.9_006063m|PACid:19258982  clementine0.9_020838m|PACid:19259150  clementine0.9_004958m|PACid:19260700  clementine0.9_035645m|PACid:19261125  clementine0.9_003140m|PACid:19263164  clementine0.9_011373m|PACid:19263102  clementine0.9_000161m|PACid:19263944  clementine0.9_000273m|PACid:19265153  clementine0.9_007574m|PACid:19263451  clementine0.9_034553m|PACid:19264939  clementine0.9_000067m|PACid:19266181  clementine0.9_016091m|PACid:19266594  clementine0.9_010964m|PACid:19269590  clementine0.9_011094m|PACid:19273678  clementine0.9_010420m|PACid:19274159  clementine0.9_006105m|PACid:19274419  clementine0.9_007139m|PACid:19275530  clementine0.9_008942m|PACid:19275606  clementine0.9_000111m|PACid:19276831  clementine0.9_009664m|PACid:19276962  clementine0.9_032361m|PACid:19276915  clementine0.9_032747m|PACid:19279908  clementine0.9_022438m|PACid:19280551  clementine0.9_022032m|PACid:19281751  clementine0.9_002868m|PACid:19283434  clementine0.9_001148m|PACid:19284776  clementine0.9_002321m|PACid:19284694 | AT2G22870.1  AT5G63640.1  AT3G27160.1  AT1G78490.1  AT4G31940.1  AT4G02080.1  AT3G16640.1  AT4G31530.1  AT1G69490.1  AT1G12770.1  AT5G08130.3  AT1G27070.1  AT1G69120.1  AT5G11240.1  AT3G12620.2  AT1G55740.1  AT1G05170.1  AT3G60860.1  AT1G04120.1  AT3G06350.1  AT2G38290.1  AT2G22125.1  AT1G61580.1  AT5G53860.2  AT5G15050.1  AT1G61190.1  AT5G42690.2  AT3G57530.1  AT3G57790.1  AT1G63490.1  AT1G64650.1  AT3G47800.1  AT2G31500.1  AT4G35020.3  AT1G07980.1  AT5G60450.1  AT4G35560.1  AT3G51070.1 | P-loop containing nucleoside triphosphate hydrolases superfamily protein  ENTH/VHS/GAT family protein  Ribosomal protein S21 family protein  Cytochrome P450, family 708, subfamily A, polypeptide 3  Cytochrome P450, family 82, subfamily C, polypeptide 4  Secretion-associated RAS super family 2  Translationally controlled tumor protein  NAD(P)-binding Rossmann-fold superfamily protein  NAC-like, activated by AP3/PI  P-loop containing nucleoside triphosphate hydrolases superfamily protein  basic helix-loop-helix (bHLH) DNA-binding superfamily protein  5\'-AMP-activated protein kinase-related  K-box region and MADS-box transcription factor family protein  Transducin family protein / WD-40 repeat family protein  Protein phosphatase 2C family protein  Seed imbibition 1  Galactosyltransferase family protein  SEC7-like guanine nucleotide exchange family protein  Multidrug resistance-associated protein 5  Dehydroquinate dehydratase, putative / shikimate dehydrogenase, putative  Ammonium transporter 2  Binding  R-protein L3 B  Embryo defective 2737  Core-2/I-branching beta-1,6-N-acetylglucosaminyltransferase family protein  LRR and NB-ARC domains-containing disease resistance protein  Protein of unknown function, DUF547  Calcium-dependent protein kinase 32  Pectin lyase-like superfamily protein  Transcription factor jumonji (jmjC) domain-containing protein  Major facilitator superfamily protein  Galactose mutarotase-like superfamily protein  Calcium-dependent protein kinase 24  RAC-like 3  Nuclear factor Y, subunit C10  Auxin response factor 4  Transducin/WD40 repeat-like superfamily protein  S-adenosyl-L-methionine-dependent methyltransferases superfamily protein |
| miR5568 | clementine0.9_000273m|PACid:19265153 | AT1G04120.1 | Multidrug resistance-associated protein 5 |
| miR5636 | clementine0.9_003795m|PACid:19252955  clementine0.9_004024m|PACid:19252048  clementine0.9_002250m|PACid:19255281  clementine0.9_032827m|PACid:19256130  clementine0.9_025195m|PACid:19256937  clementine0.9_003430m|PACid:19256988  clementine0.9_028220m|PACid:19258780  clementine0.9_022477m|PACid:19259217  clementine0.9_005901m|PACid:19260637  clementine0.9_000067m|PACid:19266181  clementine0.9_027451m|PACid:19266077  clementine0.9_002682m|PACid:19266466  clementine0.9_000088m|PACid:19267343  clementine0.9_006421m|PACid:19269259  clementine0.9_028519m|PACid:19274276  clementine0.9_000053m|PACid:19275015  clementine0.9_000654m|PACid:19275073  clementine0.9_003850m|PACid:19275238  clementine0.9_036014m|PACid:19276326  clementine0.9_013711m|PACid:19276565  clementine0.9_022615m|PACid:19276564  clementine0.9_030606m|PACid:19278667  clementine0.9_012771m|PACid:19278927  clementine0.9_022125m|PACid:19279850  clementine0.9_023685m|PACid:19279851  clementine0.9_016720m|PACid:19280007  clementine0.9_011543m|PACid:19280771  clementine0.9_003960m|PACid:19284582  clementine0.9_030973m|PACid:19284921  clementine0.9_001674m|PACid:19285684  clementine0.9_030997m|PACid:19286740 | AT2G01190.1  AT3G01750.1  AT1G80410.1  AT4G27410.2  AT5G53560.1  AT5G11350.1  AT3G04650.1  AT1G14345.1  AT5G60020.1  AT2G22125.1  AT1G19260.1  AT4G38180.1  AT2G36490.1  AT5G22090.1  AT1G47710.1  AT5G53460.1  AT5G46330.1  AT5G52640.1  AT4G26090.1  AT1G64500.1  AT3G48240.1  AT5G63020.1  AT4G10030.1  AT2G31570.1  AT4G31870.1  AT4G01580.1  AT4G34640.1  AT2G23450.2  AT3G04290.1  AT2G05170.1  AT2G27110.1 | Octicosapeptide/Phox/Bem1p family protein  Ankyrin repeat family protein  Tetratricopeptide repeat (TPR)-containing protein  NAC (No Apical Meristem) domain transcriptional regulator superfamily protein  Cytochrome B5 isoform E  DNAse I-like superfamily protein  FAD/NAD(P)-binding oxidoreductase family protein  NAD(P)-linked oxidoreductase superfamily protein  Laccase 17  Binding  TTF-type zinc finger protein with HAT dimerisation domain  FAR1-related sequence 5  Demeter-like 1  Protein of unknown function (DUF3049)  Serine protease inhibitor (SERPIN) family protein  NADH-dependent glutamate synthase 1  Leucine-rich receptor-like protein kinase family protein  Heat shock protein 90.1  NB-ARC domain-containing disease resistance protein  Glutaredoxin family protein  Octicosapeptide/Phox/Bem1p family protein  Disease resistance protein (CC-NBS-LRR class) family  Alpha/beta-Hydrolases superfamily protein  Glutathione peroxidase 2  Glutathione peroxidase 7  AP2/B3-like transcriptional factor family protein  Squalene synthase 1  Protein kinase superfamily protein  Li-tolerant lipase 1  Vacuolar protein sorting 11  FAR1-related sequence 3 |
| miR5661 | clementine0.9_023445m|PACid:19284697 | AT2G18250.1 | 4-Phosphopantetheine adenylyltransferase |
| miR5662 | clementine0.9_032335m|PACid:19268113  clementine0.9_001109m|PACid:19277954  clementine0.9_004810m|PACid:19281921  clementine0.9_007143m|PACid:19251539  clementine0.9_036075m|PACid:19251648  clementine0.9_012855m|PACid:19251976  clementine0.9_000974m|PACid:19252511  clementine0.9_023042m|PACid:19252794  clementine0.9_009724m|PACid:19254265  clementine0.9_015153m|PACid:19254187  clementine0.9_032680m|PACid:19256357  clementine0.9_035150m|PACid:19258081  clementine0.9_003250m|PACid:19259365  clementine0.9_015545m|PACid:19258980  clementine0.9_027225m|PACid:19260078 | AT5G49290.1  AT1G58190.2  AT3G18670.1  AT2G30390.1  AT4G37460.1  AT5G11000.1  AT1G10170.1  AT4G23630.1  AT1G54820.1  AT4G02590.1  AT3G60750.2  AT5G15630.1  AT1G05460.1  AT1G59960.1  AT1G61700.1 | Receptor like protein 56  Receptor like protein 9  Ankyrin repeat family protein  Ferrochelatase 2  Tetratricopeptide repeat (TPR)-like superfamily protein  Plant protein of unknown function (DUF868)  NF-X-like 1  VIRB2-interacting protein 1  Protein kinase superfamily protein  Basic helix-loop-helix (bHLH) DNA-binding superfamily protein  Transketolase  COBRA-like extracellular glycosyl-phosphatidyl inositol-anchored protein family  P-loop containing nucleoside triphosphate hydrolases superfamily protein  NAD(P)-linked oxidoreductase superfamily protein  RNA polymerases N / 8 kDa subunit |
| miR5667 | clementine0.9_003001m|PACid:19252244 | AT5G14210.1 | Leucine-rich repeat protein kinase family protein |
| miR780 | clementine0.9_005097m|PACid:19255386  clementine0.9_028378m|PACid:19253092  clementine0.9_014072m|PACid:19256699  clementine0.9_021090m|PACid:19256638  clementine0.9_004752m|PACid:19256811  clementine0.9_000314m|PACid:19259657  clementine0.9_014838m|PACid:19259702  clementine0.9_018427m|PACid:19260774  clementine0.9_002413m|PACid:19261817  clementine0.9_007538m|PACid:19262302  clementine0.9_015667m|PACid:19262934  clementine0.9_034943m|PACid:19262376  clementine0.9_019635m|PACid:19265110  clementine0.9_035426m|PACid:19263991  clementine0.9_027139m|PACid:19265435  clementine0.9_014769m|PACid:19268004  clementine0.9_025907m|PACid:19268018  clementine0.9_031839m|PACid:19268494  clementine0.9_015370m|PACid:19270345  clementine0.9_008108m|PACid:19270923  clementine0.9_004479m|PACid:19272053  clementine0.9_018684m|PACid:19273525  clementine0.9_032350m|PACid:19273738  clementine0.9_002626m|PACid:19273098  clementine0.9_016818m|PACid:19277055  clementine0.9_006177m|PACid:19279069  clementine0.9_028459m|PACid:19279948  clementine0.9_030600m|PACid:19280678  clementine0.9_001471m|PACid:19281353  clementine0.9_012283m|PACid:19285224  clementine0.9_013005m|PACid:19285553  clementine0.9_000862m|PACid:19286788  clementine0.9_010554m|PACid:19286898 | AT2G37080.1  AT5G27240.1  AT5G18260.1  AT5G39150.1  AT2G30990.1  AT1G10760.1  AT3G57030.1  AT1G49330.1  AT3G43220.1  AT5G58010.1  AT5G57230.1  AT4G02030.1  AT3G03760.1  AT5G26980.1  AT1G14450.1  AT2G38300.1  AT2G20142.1  AT1G17020.1  AT4G06599.1  AT2G44200.1  AT2G14680.3  AT4G12790.5  AT3G47890.1  AT2G34300.2  AT4G23730.1  AT4G15420.1  AT5G06850.1  AT1G19260.1  AT4G27220.1  AT4G37060.1  AT1G09390.1  AT3G03380.1  AT5G17230.2 | ROP interactive partner 3  Heat shock N-terminal domain-containing protein  RING/U-box superfamily protein  RmlC-like cupins superfamily protein  Protein of unknown function (DUF688)  Pyruvate phosphate dikinase, PEP/pyruvate binding domain  Calcium-dependent phosphotriesterase superfamily protein  Hydroxyproline-rich glycoprotein family protein  Phosphoinositide phosphatase family protein  LJRHL1-like 3  Thioredoxin superfamily protein  Vps51/Vps67 family (components of vesicular transport) protein  LOB domain-containing protein 20  Syntaxin of plants 41  NADH dehydrogenase (ubiquinone)s  Myb-like HTH transcriptional regulator family protein  Toll-Interleukin-Resistance (TIR) domain family protein  Senescence-related gene 1  Ubiquitin family protein  CBF1-interacting co-repressor CIR, N-terminal;Pre-mRNA splicing factor  Myosin heavy chain-related  P-loop containing nucleoside triphosphate hydrolases superfamily protein  Ubiquitin carboxyl-terminal hydrolase-related protein  S-adenosyl-L-methionine-dependent methyltransferases superfamily protein  Galactose mutarotase-like superfamily protein  Ubiquitin fusion degradation UFD1 family protein  C2 calcium/lipid-binding plant phosphoribosyltransferase family protein  TTF-type zinc finger protein with HAT dimerisation domain  NB-ARC domain-containing disease resistance protein  PATATIN-like protein 5  GDSL-like Lipase/Acylhydrolase superfamily protein  DegP protease 7  PHYTOENE SYNTHASE |
| miR782 | clementine0.9_012930m|PACid:19252541 | AT2G19810.1 | CCCH-type zinc finger family protein |
| miR783 | clementine0.9_033873m|PACid:19262573  clementine0.9_017903m|PACid:19252353  clementine0.9_012236m|PACid:19253331  clementine0.9_014862m|PACid:19266273  clementine0.9_023622m|PACid:19267191  clementine0.9_008635m|PACid:19275387  clementine0.9_002805m|PACid:19286713 | AT3G43190.1  AT3G27310.1  AT1G22440.1  AT5G20400.1  AT2G36410.2  AT4G25340.1  AT2G24030.1 | Sucrose synthase 4  Plant UBX domain-containing protein 1  Zinc-binding alcohol dehydrogenase family protein  2-Oxoglutarate (2OG) and Fe(II)-dependent oxygenase superfamily protein  Family of unknown function (DUF662)  FK506 BINDING PROTEIN 53  Zinc ion binding;nucleic acid binding |
| miR830 | clementine0.9_001654m|PACid:19262988  clementine0.9_006132m|PACid:19267778  clementine0.9_009426m|PACid:19268783  clementine0.9_029545m|PACid:19275269  clementine0.9_009532m|PACid:19277349  clementine0.9_010835m|PACid:19286168 | AT5G57990.1  AT5G03940.1  AT5G38510.1  AT3G49170.1  AT4G10790.1  AT2G38960.3 | Ubiquitin-specific protease 23  Chloroplast signal recognition particle 54 kDa subunit  Rhomboid-related intramembrane serine protease family protein  Tetratricopeptide repeat (TPR)-like superfamily protein  UBX domain-containing protein  Endoplasmic reticulum oxidoreductins 2 |
| miR843 | clementine0.9_001000m|PACid:19262594  clementine0.9_002356m|PACid:19251663  clementine0.9_035942m|PACid:19254789  clementine0.9_001207m|PACid:19256445  clementine0.9_028012m|PACid:19257732  clementine0.9_001048m|PACid:19262248  clementine0.9_016795m|PACid:19262945  clementine0.9_006762m|PACid:19270896  clementine0.9_022754m|PACid:19274962  clementine0.9_005522m|PACid:19282473  clementine0.9_009661m|PACid:19284701 | AT5G56040.2  AT2G22400.1  AT3G02650.1  AT1G76390.2  AT3G22600.1  AT3G42170.1  AT5G21950.1  AT4G04720.1  AT5G42900.1  AT1G16720.1  AT5G66500.1 | Leucine-rich receptor-like protein kinase family protein  S-adenosyl-L-methionine-dependent methyltransferases superfamily protein  Tetratricopeptide repeat (TPR)-like superfamily protein  ARM repeat superfamily protein  Bifunctional inhibitor/lipid-transfer protein/seed storage 2S albumin superfamily protein  BED zinc finger ;hAT family dimerisation domain  Alpha/beta-Hydrolases superfamily protein  Calcium-dependent protein kinase 21  Cold regulated gene 27  High chlorophyll fluorescence phenotype 173  Tetratricopeptide repeat (TPR)-like superfamily protein |
| miR858 | clementine0.9_036025m|PACid:19275048  clementine0.9_035953m|PACid:19261636  clementine0.9_000428m|PACid:19262527  clementine0.9_023603m|PACid:19273670  clementine0.9_006648m|PACid:19284927 | AT4G08850.1  AT3G14470.1  AT3G14460.1  AT4G32870.1  AT2G26170.1 | Leucine-rich repeat receptor-like protein kinase family protein  NB-ARC domain-containing disease resistance protein  LRR and NB-ARC domains-containing disease resistance protein  Polyketide cyclase/dehydrase and lipid transport superfamily protein  Cytochrome P450, family 711, subfamily A, polypeptide 1 |
| miR946 | clementine0.9_000146m|PACid:19276272  clementine0.9_035942m|PACid:19254789  clementine0.9_023083m|PACid:19276453  clementine0.9_019972m|PACid:19284058 | AT4G12010.1  AT3G02650.1  AT1G63800.1  AT3G07990.1 | Disease resistance protein (TIR-NBS-LRR class) family  Tetratricopeptide repeat (TPR)-like superfamily protein  Ubiquitin-conjugating enzyme 5  Serine carboxypeptidase-like 27 |
| miR948 | clementine0.9_016179m|PACid:19254450  clementine0.9_016531m|PACid:19251424  clementine0.9_015818m|PACid:19253521  clementine0.9_032853m|PACid:19255870  clementine0.9_034224m|PACid:19256612  clementine0.9_007646m|PACid:19257867  clementine0.9_027589m|PACid:19258433  clementine0.9_000583m|PACid:19259876  clementine0.9_006220m|PACid:19259379  clementine0.9_010867m|PACid:19260037  clementine0.9_003148m|PACid:19261431  clementine0.9_002644m|PACid:19264823  clementine0.9_001964m|PACid:19265674  clementine0.9_010708m|PACid:19267120  clementine0.9_025279m|PACid:19267830  clementine0.9_020794m|PACid:19269391  clementine0.9_010470m|PACid:19270943  clementine0.9_005199m|PACid:19273143  clementine0.9_018730m|PACid:19275292  clementine0.9_034110m|PACid:19275167  clementine0.9_002692m|PACid:19276163  clementine0.9_007547m|PACid:19276886  clementine0.9_006783m|PACid:19278773  clementine0.9_031453m|PACid:19280839  clementine0.9_008501m|PACid:19282818 | AT3G56850.1  AT5G67370.1  AT5G49700.1  AT1G31150.1  AT3G14470.1  AT5G07990.1  AT1G26355.1  AT5G13010.1  AT3G26310.1  AT1G24590.1  AT5G04670.1  AT5G43990.2  AT3G45140.1  AT3G22142.1  AT5G56940.1  AT4G27540.1  AT4G18760.1  AT1G34190.1  AT4G25130.1  AT5G07500.1  AT1G34300.1  AT1G32640.1  AT3G15620.1  AT1G19260.1  AT2G36750.1 | ABA-responsive element binding protein 3  Protein of unknown function (DUF1230)  Predicted AT-hook DNA-binding family protein  Domain of unknown function (DUF1985)  NB-ARC domain-containing disease resistance protein  Cytochrome P450 superfamily protein  SPIRAL1-like1  RNA helicase family protein  cytochrome P450, family 71, subfamily B, polypeptide 35  DORNROSCHEN-like  Enhancer of polycomb-like transcription factor protein  SET-domain containing protein lysine methyltransferase family protein  Lipoxygenase 2  Bifunctional inhibitor/lipid-transfer protein/seed storage 2S albumin superfamily protein  Ribosomal protein S16 family protein  Prenylated RAB acceptor 1.H  Receptor like protein 51  NAC domain containing protein 17  Peptide met sulfoxide reductase 4  Zinc finger C-x8-C-x5-C-x3-H type family protein  Lectin protein kinase family protein  Basic helix-loop-helix (bHLH) DNA-binding family protein  DNA photolyase family protein  TTF-type zinc finger protein with HAT dimerisation domain  UDP-glucosyl transferase 73C1 |
| miR1135 | clementine0.9_030066m|PACid:19271386 | AT4G15880.1 | Cysteine proteinases superfamily protein |
| miR1518 | clementine0.9_033004m|PACid:19271054 | AT4G18260.1 | Cytochrome b561/ferric reductase transmembrane protein family |
| miR3946 | clementine0.9_003112m|PACid:19251116 | AT5G27270.1 | Tetratricopeptide repeat (TPR)-like superfamily protein |
| miR4398 | clementine0.9_031483m|PACid:19263764 | AT2G46915.1 | Protein of unknown function (DUF3754) |
