# Supplementary material for: Identification of boron-deficiency-responsive microRNAs in Citrus sinensis roots by Illumina sequencing
Source: BMC Plant Biol. 2014 May 7;14:123. doi: 10.1186/1471-2229-14-123 (PMC4041134; doi:10.1186/1471-2229-14-123)
Supplement: Additional file 7 — List of target genes for parts of novel miRNAs in Citrus sinensis roots. [file 1471-2229-14-123-S7.doc]

**Additional file 7: List of target genes for parts of novel miRNAs in *Citrus sinensis*** roots

| *miRNA* | *Assession* | *Homology* | *Target genes* |
| --- | --- | --- | --- |
| novel_mir_13 | clementine0.9_011592m|PACid:19256995  clementine0.9_009423m|PACid:19274760  clementine0.9_002581m|PACid:19261769  clementine0.9_002478m|PACid:19263262  clementine0.9_005334m|PACid:19277764 | AT5G11320.1  AT1G47128.1  AT3G22910.1  AT2G18940.1  AT1G71350.1 | Flavin-binding monooxygenase family protein  Granulin repeat cysteine protease family protein  ATPase E1-E2 type family protein / haloacid dehalogenase-like hydrolase family protein  Tetratricopeptide repeat (TPR)-like superfamily protein  Eukaryotic translation initiation factor SUI1 family protein |
| novel_mir_135 | clementine0.9_003290m|PACid:19257361  clementine0.9_002827m|PACid:19258257  clementine0.9_028913m|PACid:19283071 | AT3G23410.1  AT2G15820.1  AT3G20580.1 | Fatty alcohol oxidase 3  Endonucleases  COBRA-like protein 10 precursor |
| novel_mir_207 | clementine0.9_011823m|PACid:19269777  clementine0.9_031745m|PACid:19275745  clementine0.9_033204m|PACid:19275934  clementine0.9_000031m|PACid:19286191  clementine0.9_033352m|PACid:19286644 | AT2G17030.1  AT1G78090.1  AT1G35910.1  AT1G65810.1  AT2G23945.1 | F-box family protein with a domain of unknown function (DUF295)  Trehalose-6-phosphate phosphatase  Haloacid dehalogenase-like hydrolase (HAD) superfamily protein  P-loop containing nucleoside triphosphate hydrolases superfamily protein  Eukaryotic aspartyl protease family protein |
| novel_mir_212 | clementine0.9_015773m|PACid:19252184  clementine0.9_001210m|PACid:19257602  clementine0.9_000023m|PACid:19258236  clementine0.9_017033m|PACid:19260579  clementine0.9_006359m|PACid:19262358  clementine0.9_001664m|PACid:19272032  clementine0.9_009423m|PACid:19274760  clementine0.9_018550m|PACid:19274415  clementine0.9_032931m|PACid:19276358  clementine0.9_005334m|PACid:19277764 | AT5G14130.1  AT3G22910.1  AT3G19050.1  AT3G06010.1  AT2G12550.1  AT4G35270.1  AT1G47128.1  AT1G47720.1  AT4G34138.1  AT1G71350.1 | Peroxidase superfamily protein  ATPase E1-E2 type family protein / haloacid dehalogenase-like hydrolase family protein  Phragmoplast orienting kinesin 2  Homeotic gene regulator  Ubiquitin-associated (UBA)/TS-N domain-containing protein  Plant regulator RWP-RK family protein  Granulin repeat cysteine protease family protein  Primosome PriB/single-strand DNA-binding  UDP-glucosyl transferase 73B1  Eukaryotic translation initiation factor SUI1 family protein |
| novel_mir_371 | clementine0.9_010675m|PACid:19263245  clementine0.9_032741m|PACid:19283533 | AT3G13050.1  AT1G69310.1 | Major facilitator superfamily protein  WRKY DNA-binding protein 57 |
| novel_mir_379 | clementine0.9_011140m|PACid:19254203  clementine0.9_003847m|PACid:19253405  clementine0.9_000953m|PACid:19255379  clementine0.9_028019m|PACid:19255979  clementine0.9_004249m|PACid:19256372  clementine0.9_035676m|PACid:19258534  clementine0.9_021105m|PACid:19263765  clementine0.9_036074m|PACid:19271269  clementine0.9_012120m|PACid:19273247  clementine0.9_002490m|PACid:19274662  clementine0.9_019959m|PACid:19285181  clementine0.9_000258m|PACid:19286118 | AT3G05530.1  AT4G24490.2  AT5G02950.1  AT5G19390.2  AT2G33730.1  AT3G28630.1  AT2G44840.1  AT4G13650.1  AT1G80880.1  AT5G42920.2  AT4G36910.1  AT2G34780.1 | Regulatory particle triple-A ATPase 5A  RAB geranylgeranyl transferase alpha subunit 1  Tudor/PWWP/MBT superfamily protein  Rho GTPase activation protein (RhoGAP) with PH domain  P-loop containing nucleoside triphosphate hydrolases superfamily protein  Protein of unknown function (DUF569)  Ethylene-responsive element binding factor 13  Pentatricopeptide repeat (PPR) superfamily protein  Tetratricopeptide repeat (TPR)-like superfamily protein  THO complex, subunit 5  Cystathionine beta-synthase (CBS) family protein  Maternal effect embryo arrest 22 |
| novel_mir_40 | clementine0.9_004028m|PACid:19266718  clementine0.9_000825m|PACid:19272879  clementine0.9_009713m|PACid:19272748  clementine0.9_011340m|PACid:19272749 | AT1G12240.1  AT1G21730.1  AT1G77480.1  AT1G44130.1 | Glycosyl hydrolases family 32 protein  P-loop containing nucleoside triphosphate hydrolases superfamily protein  Eukaryotic aspartyl protease family protein  Eukaryotic aspartyl protease family protein |
| novel_mir_42 | clementine0.9_001923m|PACid:19255789  clementine0.9_000636m|PACid:19254755  clementine0.9_002202m|PACid:19255082  clementine0.9_014584m|PACid:19273291 | AT3G46530.1  AT4G27220.1  AT5G63020.1  AT5G11790.1 | NB-ARC domain-containing disease resistance protein  NB-ARC domain-containing disease resistance protein  Disease resistance protein (CC-NBS-LRR class) family  N-MYC downregulated-like 2 |
| novel_mir_464 | clementine0.9_029104m|PACid:19276547  clementine0.9_012798m|PACid:19254482  clementine0.9_002307m|PACid:19283422 | AT3G26300.1  AT1G31410.1  AT1G20780.1 | Cytochrome P450, family 71, subfamily B, polypeptide 34  Putrescine-binding periplasmic protein-related  Senescence-associated E3 ubiquitin ligase 1 |
| novel_mir_47 | clementine0.9_002625m|PACid:19251244  clementine0.9_021452m|PACid:19251291  clementine0.9_015773m|PACid:19252184  clementine0.9_001985m|PACid:19255605  clementine0.9_005561m|PACid:19256865  clementine0.9_001210m|PACid:19257602  clementine0.9_028664m|PACid:19257455  clementine0.9_008090m|PACid:19261245  clementine0.9_017033m|PACid:19260579  clementine0.9_002581m|PACid:19261769  clementine0.9_006359m|PACid:19262358  clementine0.9_005634m|PACid:19265579  clementine0.9_029302m|PACid:19265741  clementine0.9_000337m|PACid:19267688  clementine0.9_018413m|PACid:19267988  clementine0.9_027791m|PACid:19268639  clementine0.9_027496m|PACid:19270328  clementine0.9_008622m|PACid:19270661  clementine0.9_010107m|PACid:19271396  clementine0.9_001750m|PACid:19273339  clementine0.9_027627m|PACid:19273274  clementine0.9_018550m|PACid:19274415  clementine0.9_027859m|PACid:19274852  clementine0.9_032931m|PACid:19276358  clementine0.9_001002m|PACid:19277171  clementine0.9_005334m|PACid:19277764  clementine0.9_016025m|PACid:19279346  clementine0.9_035560m|PACid:19280442  clementine0.9_001170m|PACid:19281105  clementine0.9_000518m|PACid:19281756  clementine0.9_010183m|PACid:19282538  clementine0.9_015416m|PACid:19284416  clementine0.9_013331m|PACid:19284482 | AT4G37270.1  AT2G22540.1  AT5G14130.1  AT2G04270.2  AT3G59040.1  AT3G22910.1  AT3G63380.1  AT3G16760.1  AT3G06010.1  AT3G22910.1  AT2G12550.1  AT1G73730.1  AT5G60900.1  AT2G36380.1  AT5G06410.1  AT2G01210.1  AT3G18080.1  AT1G47128.1  AT5G45820.1  AT1G12280.1  AT4G27190.1  AT1G47720.1  AT3G14470.1  AT4G34138.1  AT4G11110.1  AT1G71350.1  AT1G05010.1  AT5G26600.2  AT1G70750.1  AT3G46960.1  AT5G59580.1  AT2G40620.1  AT5G43060.1 | Heavy metal atpase 1  K-box region and MADS-box transcription factor family protein  Peroxidase superfamily protein  RNAse E/G-like  Tetratricopeptide repeat (TPR)-like superfamily protein  ATPase E1-E2 type family protein / haloacid dehalogenase-like hydrolase family protein  ATPase E1-E2 type family protein / haloacid dehalogenase-like hydrolase family protein  Tetratricopeptide repeat (TPR)-like superfamily protein  Homeotic gene regulator  ATPase E1-E2 type family protein / haloacid dehalogenase-like hydrolase family protein  Ubiquitin-associated (UBA)/TS-N domain-containing protein  ETHYLENE-INSENSITIVE3-like 3  Receptor-like protein kinase 1  Pleiotropic drug resistance 6  DNAJ heat shock N-terminal domain-containing protein  Leucine-rich repeat protein kinase family protein  B-S glucosidase 44  Granulin repeat cysteine protease family protein  CBL-interacting protein kinase 20  LRR and NB-ARC domains-containing disease resistance protein  NB-ARC domain-containing disease resistance protein  Primosome PriB/single-strand DNA-binding  NB-ARC domain-containing disease resistance protein  UDP-glucosyl transferase 73B1  SPA1-related 2  Eukaryotic translation initiation factor SUI1 family protein  Ethylene-forming enzyme  Pyridoxal phosphate (PLP)-dependent transferases superfamily protein  Protein of unknown function, DUF593  RNA helicase, ATP-dependent, SK12/DOB1 protein  UDP-glucosyl transferase 76E1  Basic-leucine zipper (bZIP) transcription factor family protein  Granulin repeat cysteine protease family protein |
| novel_mir_470 | clementine0.9_002420m|PACid:19255038  clementine0.9_009794m|PACid:19260556  clementine0.9_002189m|PACid:19281775 | AT1G52150.1  AT3G06330.1  AT5G02390.1 | Homeobox-leucine zipper family protein / lipid-binding START domain-containing protein  RING/U-box superfamily protein  Protein of unknown function (DUF3741) |
| novel_mir_471 | clementine0.9_002420m|PACid:19255038  clementine0.9_002604m|PACid:19254835  clementine0.9_000962m|PACid:19270198  clementine0.9_002189m|PACid:19281775  clementine0.9_002294m|PACid:19282243  clementine0.9_002251m|PACid:19286208 | AT1G52150.1  AT4G29750.1  AT2G21300.2  AT5G02390.1  AT5G60690.1  AT2G34710.1 | Homeobox-leucine zipper family protein / lipid-binding START domain-containing protein  CRS1 / YhbY (CRM) domain-containing protein  ATP binding microtubule motor family protein  Protein of unknown function (DUF3741)  Homeobox-leucine zipper family protein / lipid-binding START domain-containing protein  Homeobox-leucine zipper family protein / lipid-binding START domain-containing protein |
| novel_mir_52 | clementine0.9_023574m|PACid:19252334  clementine0.9_008011m|PACid:19259369  clementine0.9_008959m|PACid:19258968  clementine0.9_016441m|PACid:19260022  clementine0.9_012563m|PACid:19264657 | AT1G53160.1  AT1G69170.1  AT5G43270.1  AT5G50570.1  AT2G42200.1 | Squamosa promoter binding protein-like 4  Squamosa promoter-binding protein-like (SBP domain) transcription factor family protein  Squamosa promoter binding protein-like 2  Squamosa promoter-binding protein-like (SBP domain) transcription factor family protein  Squamosa promoter binding protein-like 9 |
| novel_mir_79 | clementine0.9_000163m|PACid:19256212  clementine0.9_012443m|PACid:19262305 | AT2G13370.1  AT3G20480.1 | Chromatin remodeling 5  Tetraacyldisaccharide 4\'-kinase family protein |
| novel_mir_95 | clementine0.9_026957m|PACid:19262146  clementine0.9_006981m|PACid:19264804  clementine0.9_003545m|PACid:19273795  clementine0.9_009002m|PACid:19273696 | AT5G57815.1  AT2G47310.1  AT2G25930.1  AT1G80870.1 | Cytochrome c oxidase, subunit Vib family protein  Flowering time control protein-related / FCA gamma-related  Hydroxyproline-rich glycoprotein family protein  Protein kinase superfamily protein |
| novel_mir_98 | clementine0.9_034815m|PACid:19259904  clementine0.9_003955m|PACid:19269403 | AT3G26610.1  AT3G14470.1 | Pectin lyase-like superfamily protein  NB-ARC domain-containing disease resistance protein |
