# Supplementary material for: Identification of boron-deficiency-responsive microRNAs in Citrus sinensis roots by Illumina sequencing
Source: BMC Plant Biol. 2014 May 7;14:123. doi: 10.1186/1471-2229-14-123 (PMC4041134; doi:10.1186/1471-2229-14-123)
Supplement: Additional file 8 — Primer sequences for qRT-PCR expression analysis of miRNAs. [file 1471-2229-14-123-S8.doc]

**Additional file 8: Primer sequences for qRT-PCR expression analysis of miRNAs. Uni-miR qPCR p**rimer was added as the common reverse primer.

| *miRNAs* | *miRNA sequences* | *Forward primers (5´→3´)* |
| --- | --- | --- |
| miR1077 | TTAAGTGTTCGGATCGCGGC | GTCGGTTAAGTGTTCGGATCGCG |
| miR1523 | ATGGGATAAAGTAGGTAA | CGGCGATGGGATAAAGTAGGT |
| miR159 | TTTGGATTGAAGGGAGCTCTA | CGGGTTTGGATTGAAGGGAGC |
| miR2079 | AGAGTTATGTTGATCGACGTA | GAGCGAGAGTTATGTTGATCGAC |
| miR3440 | TGGATTGGACAAGAAAGGT | GGCGGTGGATTGGACAAGAAAG |
| miR3462 | GTTCGGGTTCGGGCGACG | GTCTCGTTCGGGTTCGGGCG |
| miR5137 | AGCGAAGAGAGAAAGATGGGCT | GCGTAGCGAAGAGAGAAAGATGG |
| miR5142 | AGTAATGATTGATAGGGAT | GGCGGAGTAATGATTGATAGGG |
| miR535 | TGACAATGAGAGAGAGCACAC | CCGATGACAATGAGAGAGAGCAC |
| miR5559 | TCTGGTGAAGTGTTCGGATC | GGTCTGGTGAAGTGTTCGGATC |
| miR5568 | CAGACGACTTAAATTCGCGA | GCGGCGCAGACGACTTAAATTC |
| miR1446 | CGAACTCTCTCCCTCAAC | GACTCCGAACTCTCTCCCTCAAC |
| miR1439 | TTTTGGAAGGGATGTATT | CGGCGTTTTGGAAGGGATGTAT |
| miR1039 | GGGGGACGGGCTGGGATC | CATTATGGGGGACGGGCTGGGA |
| miR1875 | AAATGTGAGTGGAGTGGACAGAA | GTGCAAATGTGAGTGGAGTGGAC |
| miR2592 | GGAAAACAGAATGTGCGGGATC | CGAAGGAAAACAGAATGTGCGGG |
| miR407 | TTTAATCAAATACTTTCTTGT | GCTGGCTTTAATCAAATACTTTCTTG |
| miR2948 | TATGGGAGAGTAGGGAAGAT | CGGAGTATGGGAGAGTAGGGAAG |
| miR3454 | ATTCCCATCGAGCTGGGTA | GCCACATTCCCATCGAGCTG |
| miR3456 | TTTAGAACGTCGTGAGTCA | GCGCTTTAGAACGTCGTGAGTC |
| miR3932 | AAGTTTTGATGACAAGAA | GCGGAAGTTTTGATGACAAGAA |
| miR3946 | ATTGTAGAGAATTAGAAGAACAC | CGCGCATTGTAGAGAATTAGAAGAAC |
| miR5037 | AGTGAGAACTTTGAAGGCCGA | ACCGTAGTGAGAACTTTGAAGGCC |
| miR5259 | CAAGGGGTATTTGGATGGACA | CGCACAAGGGGTATTTGGATGGAC |
| miR5667 | AAACAGATCAAAGATGGCATTTCC | GCGCAAACAGATCAAAGATGGCATTTC |
| miR1160 | AAGGACCCTCGGAAGGGG | GGAACAAGGACCCTCGGAAG |
|  |  |  |
|  | Reverse primers (5´→3´) | Forward primers (5´→3´) |
| *Actin* | GCTTGGAGCAAGTGCTGTGATT | AGAACTATGAACTGCCTGATGGC |
