# Supplementary material for: Identification of boron-deficiency-responsive microRNAs in Citrus sinensis roots by Illumina sequencing
Source: BMC Plant Biol. 2014 May 7;14:123. doi: 10.1186/1471-2229-14-123 (PMC4041134; doi:10.1186/1471-2229-14-123)
Supplement: Additional file 9 — Specific primer pairs used for qRT-PCR expression analysis of selected miRNA target genes. [file 1471-2229-14-123-S9.doc]

**Additional file 9: Specific primer pairs used for qRT-PCR expression analysis of selected miRNA target genes.**

| **miRNA** | **Accession No.** | **Target gene** | **Forward primer (5´→3´)** | **Reverse primer (5´→3´)** |
| --- | --- | --- | --- | --- |
| **miR157** | **At1g27370** | ***SPL10*** | **ATTCCAATGGGCTCTTGCTATC** | **AGACTTGTACTTGTGGGGTGCTG** |
|  | **At3g57920** | ***SPL15*** | **CTGGGAACATTGTGCCGAAAA** | **CCCAAAACAAACAGCAGCCAT** |
|  | **At5g43270** | ***SPL2*** | **CGTGATGATTCCAATGGGCTCT** | **GACTTGTACTCGTGGGGTGCTG** |
|  | **AT2G33810.1** | ***SPL3*** | **GCCAAGGCTCCTGTTGTTCG** | **CGGCAACTCCTTTTGGTGTCA** |
|  | **At2g42200** | ***SPL9*** | **CATTGTTGCTCGGCACCTCA** | **GCCCCAGTTATGCTGCTCAGTT** |
|  | **At1g69170** | ***SPL6*** | **CCTGATGCCGGTCATGCTT** | **TCCTCTGTTGCTCCACCCTTT** |
|  | **At5g50570** | ***SPL13, SPL13A*** | **CCTGTTTGGAGTGGCATAGTCA** | **GAACTGCTTCCCTTCTTTGTCG** |
|  | **At1g53160** | ***SPL4*** | **TGGCTGGACATAATGAACGAAG** | **TTCCCGCAGACCAAATCCT** |
|  | **AT5G45650.1** | ***Subtilase family protein*** | **AGCCACGGATTCTCATTTACCA** | **TCGGATTTGCCTTGACGGA** |
| **miR158** | **AT2G03210** | ***Fucosyltransferase 2*** | **AGTGGTTGCTGTTTATCAGGCTAG** | **CCTCAAATATGGAACAAGAGTCGC** |
|  | **AT3G07400** | ***Lipase class 3 family protein*** | **CAAGCATCTCCTAGCACCACTG** | **CTCCCATCTGAATCTCCACCC** |
| **miR165** | **At5g60690** | ***IFL1/REV*** | **TCATCTGTTCAGAGGGTGGCC** | **GAGTTGTCTCCAGCATGTCAAGG** |
|  | **At4g32880** | ***ATHB-8*** | **AATGGCTAGGCAGTATGTCCGT** | **CAGCACAAAACCGCATCAGA** |
|  | **At1g52150** | ***ATHB-15*** | **GGTGGCATTAGCCCTATCTCC** | **CAGTGCAACCAAGGTCGTCTC** |
| **miR2118** | **AT3G14460.1** | ***LRR and NB-ARC domains-containing disease resistance protein*** | **TCCAGGCATTGTATTCTTTCCG** | **CGCAGCATCTGAGCATCCATTA** |
|  | **AT5G17680.1** | ***TIR-NBS-LRR domain protein*** | **CCGCAACTTCCACCCAACA** | **GGACATGAAAGACGCAGCAGAC** |
| **miR472** | **AT4G27190.1** | ***Disease resistance protein (TIR-NBS-LRR class) family*** | **ATTGCGTTCCTGTGGCTGTC** | **CTCTTGAATCTGGGGCGTGA** |
|  | **AT5G63020.1** | ***LRR and NB-ARC domains-containing disease resistance protein*** | **CCGCAACCTTCGTCATCTATCT** | **TTTCTTAAACCTGGGCAGTCGT** |
|  | **AT1G12210.1** | ***Disease resistance protein (CC-NBS-LRR class) family*** | **AACCATGCCGTTCCCTTCTC** | **TCCTCCCACTGTAACTGCTCCC** |
| **miR782** | **AT2G19810.1** | ***CCCC-type zinc finger family protein*** | **GACTTGCGGGCGAAAATGT** | **AGCTCAGTGATCCAACCCAGAT** |
| **miR830** | **At1g52380** | ***RanBP1 domain*** | **GAGCGTGGGAAGGGAGAACT** | **TATCTTTATGAGCGGCAACAGC** |
|  | **At3g45850** | ***Kinesin motor-related*** | **TGTTAGAGGAGGAATGGAAGCC** | **TTCTCGGTGTTGAACAAGATGG** |
| **miR843** | **TC375153** | ***Leucine rich repeat protein*** | **TGGGGACTGCTCCAGCTTATC** | **TCACTTGGGCCATGCTTACC** |
|  | **At5g13550** | ***Sulphate transporter*** | **TGACTTTGTCCAAATCGGGTGT** | **CCAACTTAGGGTCCGTGTTCTT** |
| **miR5023** | **AT3G21640.1** | ***FKBP-type peptidyl-prolyl cis-trans isomerase family protein*** | **AATGCAGCCTTGTATTGGGAGA** | **CATGTTCAGCGAGCAAACGAA** |
|  | **AT5G45160.1** | ***Root hair defective 3 GTP-binding protein (RHD3)*** | **ACATTGCGGCGGAGTTTCG** | **GCTGGTGGCGGTGTTTGATA** |
| **miR5266** | **AT4G13510.1** | ***Ammonium transporter 1;1*** | **GTTGTTGCGGGTTGGGTTAG** | **AGATGGGGTTGTAGCAGTGGG** |
| **miR5562** | **AT2G38290.1** | ***Ammonium transporter 2*** | **TCTTCTTGCCCGTCACAAACTC** | **TTCTCGCCATCACCCCACA** |
| **miR3465** | **AT3G57330.1** | ***Autoinhibited Ca^2+^-ATPase 11*** | **AGCTGGCATCAATGGCTTCTA** | **TGGACCGCTAGGGATTTCTTC** |
|  | **AEK97331.1** | ***Actin*** | **AGAACTATGAACTGCCTGATGGC** | **GCTTGGAGCAAGTGCTGTGATT** |
